# Attention Deficit Hyperactivity Disorder (ADHD) and the gut microbiome: An ecological perspective

**DOI:** 10.1371/journal.pone.0273890

**Published:** 2023-08-18

**Authors:** Trevor Cickovski, Kalai Mathee, Gloria Aguirre, Gorakh Tatke, Alejandro Hermida, Giri Narasimhan, Melanie Stollstorff

**Affiliations:** 1 Bioinformatics Research Group (BioRG), Knight Foundation School of Computing and Information Sciences, Florida International University, Miami, FL, United States of America; 2 Department of Human and Molecular Genetics, Herbert Wertheim College of Medicine, Florida International University, Miami, FL United States of America; 3 Biomolecular Sciences Institute, Florida International University, Miami, FL, United States of America; 4 Department of Biological Sciences, College of Arts, Sciences and Education, Florida International University, Miami, FL, United States of America; 5 Cognitive Neuroscience Laboratory, Department of Psychology, Florida International University, Miami, FL, United States of America; Institute for Biological Research, University of Belgrade, SERBIA

## Abstract

Attention Deficit Hyperactivity Disorder (ADHD) is an increasingly prevalent neuropsychiatric disorder characterized by hyperactivity, inattention, and impulsivity. Symptoms emerge from underlying deficiencies in neurocircuitry, and recent research has suggested a role played by the gut microbiome. The gut microbiome is an ecosystem of interdependent taxa involved in an exponentially complex web of interactions, plus host gene and reaction pathways, some of which involve neurotransmitters with roles in ADHD neurocircuitry. Studies have analyzed the ADHD gut microbiome using macroscale metrics such as diversity and differential abundance, and have proposed several taxa as elevated or reduced in ADHD compared to Control. Few studies have delved into the complex underlying dynamics ultimately responsible for the emergence of such metrics, leaving a largely incomplete, sometimes contradictory, and ultimately inconclusive picture. We aim to help complete this picture by venturing beyond taxa *abundances* and into taxa *relationships* (i.e. cooperation and competition), using a publicly available gut microbiome dataset (targeted 16S, v3-4 region, qPCR) from an observational, case-control study of 30 Control (15 female, 15 male) and 28 ADHD (15 female, 13 male) undergraduate students. We first perform the same macroscale analyses prevalent in ADHD gut microbiome literature (diversity, differential abundance, and composition) to observe the degree of correspondence, or any new trends. We then estimate two-way ecological relationships by producing Control and ADHD Microbial Co-occurrence Networks (MCNs), using SparCC correlations (*p* ≤ 0.01). We perform community detection to find clusters of taxa estimated to mutually cooperate along with their centroids, and centrality calculations to estimate taxa most vital to overall gut ecology. We finally summarize our results, providing conjectures on how they can guide future experiments, some methods for improving our experiments, and general implications for the field.

## Introduction

Attention Deficit Hyperactivity Disorder (ADHD) is a significant mental health problem with a current 3.4% prevalence worldwide [1]. In the United States, ADHD affects one in 10 children (a 43% increase over the last 15 years) [2], and 3–16% of adults [3] with that percentage increasing over the past 20 years. Individuals with ADHD face many practical challenges, including risk for low academic achievement, lower employment status, and incarceration [4]. Symptoms of hyperactivity, impulsivity, and inattention characterize ADHD [5]. Underlying ADHD behavioral symptoms are deficits in the neurocognitive mechanisms of both executive function (EF) and emotional regulation (ER) [6]. EF refers to a set of cognitive control processes, including attention (specifically, one’s ability to focus on relevant information while suppressing irrelevant distractors), memory, and motor skills [7]. ER generally ascribes to one’s ability to effectively cope with emotionally charged circumstances (both negative and positive). These deficits include and extend beyond prefrontal-striatal networks [8]. As just one example, during attention-based tasks, reduced activation in the right dorsal attention network (including the right dorsolateral prefrontal cortex, the basal ganglia and thalamus, parietal lobe, and precuneus) has been reported [9]. Many medications have been developed to combat the disorder by influencing the underlying neurocircuitry [10].

The pathogenesis of ADHD is thought to be multifactorial, with heritability estimates at roughly 70–90% [11]. These genetic connections suggest some dependency on underlying metabolic reactions, directly or indirectly involving gene products. In the meantime, the new and exciting field of microbiome research has made its way into the mental health domain. Our gut is home to a plethora of bacteria, fungi, and other microbial organisms, whose collective genomes comprise our gut *microbiome*. Studies estimate that the average number of bacterial cells in humans matches or exceeds that of host cells [12, 13]. Each bacterium has unique genetic material that produces different sets of metabolites, which interact with each other and host metabolites downstream [14], creating a complex host-microbiome web of interactions. It has become increasingly important to pay attention to the symbiotic relationship between the gut microbiome and brain development and function, often referred to as the *gut-brain-microbiome axis* [15]. This axis is a bidirectional communication network, providing gut microbiota and metabolites an avenue for influencing brain development and function [16–20].

The fact that individuals with ADHD suffer from gastrointestinal (GI) dysfunction, including childhood digestive difficulties and low-grade inflammation [21] as well as constipation [22, 23], suggests a potential role of the gut microbiome in this disorder. For example, the plasma levels of the cytokine TNF-α were found to be significantly lower in ADHD children compared to healthy controls, and these levels were also found to be negatively correlated with gut microbiome diversity in these same samples [24]. Plasma short-chain fatty acid (SCFA) levels were also found as deficient in ADHD patients (both children and adults, [25]). This is particularly interesting because SCFAs are produced during bacterial fermentation and have been hypothesized to improve neuro-immunoendicrine functionality [26], and speculated to have a mediational role in microbiota-gut-brain crosstalk [27].

It has also been proposed that gut microbiota may affect our neurobiology by directly or indirectly altering the levels of *neurotransmitters*, including dopamine and serotonin (5-HT) [28], which fuel brain regions that mediate cognition and emotion. Although serotonin is also produced in the brain, up to 90% of serotonin is synthesized in the gut [29]. Connections between the gut microbiome and neurotransmitters, EF/ER, and *neuropsychiatric disorders (NPDs)* are already well-established. In rodents, anxiety and social behavior have been linked to the gut microbiome that can be attributed to altered neurotransmission in the hippocampus and amygdala [30]. In humans, associations between microbiome composition and ER have been shown [20]. It has also been established that the gut microbiome can release dopamine and 5-HT, impacting ER [31, 32]. Connections on the cognitive axis related to EF are less well-established in humans, though some theories are beginning to emerge [33]. In humans, dopamine influences EF [34]. In rodents, the gut microbiome is linked to dopamine [35], and EF-like behavior [36]. The Autism Spectrum Disorder (ASD) [37], which is associated with impaired EF [38], has been linked to the gut microbiome [39]. In animal studies, the gut microbiome has been associated with anxiety-related disorders such as depression [40–45]. People with stress-related diseases have responded positively to probiotics [46, 47]. Connections between the gut microbiome and another neuropsychiatric disorder (NPD) characterized by EF/ER dysfunction such as ADHD would further support the impact of the gut microbiome on EF/ER. It could also help to explain the large amount of symptomatic overlap that exists between ADHD with other NPDs, particularly ASD [48–50], and could even provide differentiating factors [51] to help address the current diagnosis challenges due to this overlap [52], and new potential options for treatment [53].

There are limited studies that implicate the gut microbiome on clinically diagnosed ADHD, and recent efforts have been made to survey and summarize their results [54–57]. Two in particular [55, 56] contained findings from multiple published studies involving ADHD and the gut microbiome. Based on this, we make the following observations about the current state of ADHD and gut microbiome research:

### 1. Diversity results are contradictory and inconclusive

Table 1 shows that even closely age-matched gut microbiome studies using the same Shannon index [58] to measure alpha-diversity produce contradictory results when comparing Control and ADHD cohorts. With respect to Beta-Diversity in these same studies, some report a difference between Control and ADHD samples while others do not.

**Table 1:** Closely age-matched alpha- and beta-diversity results.

Age	Alpha-Diversity (Shannon Index [58])	Beta-Diversity
Mean: 11.9 [59]	Higher in Control	Difference
Mean: 9.3 [60]	Higher in ADHD	No Difference
6–10 [61]	No Change	No Difference
10 and 15 [62]	Higher in ADHD for 15, No Change for 10	No Difference
20.2 [63]	No Change	Difference (depends on metric)

Alpha- and beta-diversity results from ADHD gut microbiome studies of similar age groups.

### 2. Results that estimate overall degree of differentiation between ADHD microbiome datasets compared to Control are inconclusive

One ADHD gut microbiome study [59] attempted the unsupervised method non-parametric multi-dimensional scaling (NMDS, [64]), but could not differentiate the two groups. Limited studies have further decomposed ADHD samples by subscale but these focus on diversity and distinguishing taxa, noting Inattention (elevated genus *Dialister* and reduced genus *Phascolarctobacterium* [63]) and Hyperactivity (lower alpha-diversity and elevated genus *Parabacteroides* [59]) properties.

### 3. Many microbial taxa have been proposed as elevated or reduced in ADHD compared to healthy controls, some contradictory, others mixed depending on taxonomic level, and others inconclusive

Table 2 summarizes these results. Taxa proposed as elevated in Control are colored orange, those proposed as elevated in ADHD samples are colored purple, and those with contradictory results are colored grey with bold text. Each includes corresponding literature citations. Note taxa have been grouped by the next highest taxonomic level (for example, genera *Ruminococcus*, *Coprococcus*, and *Lachnoclostridium* are all of the *Lachnospiraceae* family). Taxa in bold and italics had conflicting results over member taxa, in just italics (i.e., with family *Veillonellaceae*, member genus *Dialister* was reported elevated in Control, member species *V*. *parvula* was reported elevated in ADHD).

**Table 2:** Taxa proposed as elevated/reduced in ADHD.

Phylum	Class	Order	Family	Genus	Species
Actinobacteria	Actinobacteria	Bifidobacteriales	Bifidobacteriaceae	**Bifidobacterium**[65], [66]	
	Coriobacteria	Coriobacteriales	Coriobacteriaceae	Eggerthella [66]	
Fusobacteria	Fusobacteria	Fusobacteriales	Fusobacteriaceae	Fusobacterium [60]	
Firmicutes	Clostridia	**Clostridiales** [66, 67]	Lachnospiraceae [68]	Ruminococcus	R. gnavus [68]
				Coprococcus [63]	C. eutactus [66]
				Lachnoclostridium [61]	
			Ruminococcaceae [68]	Faecalibacterium [61, 68]	F. prausnitzii [68]
			Clostridiaceae	Intestinibacter [63]	
			Catabacteriaceae [59]		
			***Veillonellaceae*** [68]	Veillonella	*V*. *parvula* [68]
				*Dialister* [61]	
			Peptostreptococcaceae [61]		
			Peptococcaceae [61]		
	Bacilli	Lactobacillales	Lactobacillaceae	Lactobacillus [60]	
			Enterococcaceae [68]	Enterococcus [68]	
Bacteroidetes	Bacteroidia	Bacteroidales	**Porphyromonadaceae** [59, 66]		
			***Prevotellaceae*** [59]	*Prevotella* [59, 63]	
				Paraprevotella	*P*. *xylaniphila* [68]
			Odoribacteriaceae [68]	Odoribacter [68]	O. splanchicus [68]
			Rikenellaceae [66]		
			***Bacteroidaceae*** [59]	**Bacteroides** [59, 65]	*B*. *uniformis* [60]
					*B*. *ovatus* [60]
					*B*. *caccae* [68]
					*B*. *coprocola* [60]
				Parabacteroides [59]	
Proteobacteria	Betaproteobacteria	Neisseriales	Neisseriaceae [59]	Neisseria [59]	
		Burkholderiales	Sutterellaceae	Sutterella	S. stercoricanis [60]
			Alcaligenaceae [61]		
	Deltaproteobacteria	Desulfovibriales	Desulfovibrionaceae	Desulfovibrio [67]	
	Gammaproteobacteria	Pseudomondales	Moraxellaceae [61]		
		Xanthomonadales	Xanthomonadaceae [61]		

Taxa that have been proposed as elevated in Control (orange) and ADHD (purple). Taxa labelled grey with bold text had conflicting results over multiple studies. Taxa in bold and italics had mixed results over member sub-classifications (in normal italics).

Table 2 shows several taxa families with mixed results over member genera and species, including *Bacteroidaceae*, *Prevotellaceae*, and *Veillonellaceae* (these mixed results were actually within the same study). Conflicting results were reported for the following taxa:

#### Order clostridiales (Phylum: Firmicutes)

This order was reported as reduced in ADHD samples compared to Controls in a study with average ages of 19.5 and 27.1 in the respective sample sets [66], but a later Genome-Wide Association (GWAS, [69]) study found Clostridiales as elevated in ADHD samples compared to Controls [67].

#### Family *Porphyromonadaceae* (Phylum: Bacteroidetes)

The same study (with average ages of 19.5 and 27.1 [66]) reported elevated *Porphyromonadaceae* in ADHD samples compared to Controls, but a second gender-matched study [59] with similar mean respective ages (11.9 and 13.7) reported taxa of this family as reduced in ADHD samples compared to Controls.

#### Family *Bacteroides* (Phylum: Bacteroidetes)

The same study (mean ages 11.9 and 13.7, [59]) found members of this genus as elevated in ADHD samples compared to Controls. Another involving 18 to 24 month olds [65] found members of this genus as lower in ADHD samples compared to Controls (though it should be noted, these children did undergo probiotic intervention).

#### Genus *Bifidobacterium* (Phylum: Actinobacteria)

Perhaps no greater mystery currently exists than the role of genus *Bifidobacterium*. One Dutch study found a nominal increase in *Bifidobacterium* in ADHD with average ADHD and Control subject ages of 19.5 and 27.1 years, respectively [66]. A longitudinal study (3 months, six months and 13 years) made a somewhat contradictory observation of reduced *Bifidobacterium* in ADHD samples during infancy, but not at age 13 [65]. A third study [70] reported reduced *Bifidobacterium* (specifically species *B*. *longum* and *B*. *adolescentis*) in ADHD children (mean age: 9.3) that actually reversed after micro-nutrient treatment, where elevated *Bifidobacterium* was observed at high ADHD-Rating Scale IV (ADHD-RS-IV, [71]) scores.

The current picture of the role played by the gut microbiome in ADHD is therefore still unclear. Most of the effort to connect ADHD to the gut microbiome has involved (1) macroscale population metrics such as diversity, and/or (2) taxa abundances. These properties are in reality emergent from a complex and interdependent interaction web involving taxa, their gene products, and those of the host [72]. Diversity and abundance therefore ignore many underlying details behind their measurements, helping to explain the current incomplete picture. Venturing deeper into this web is critical to completing more of this picture. Two studies have attempted this task, both using multi-omics. One [68] reported differences ADHD neurotransmitter pathways. A second [66] uncovered a connection between *Bifidobacterium* and cyclohexadienyl dehydratase (CDT) abundances.

We have thus far only scratched the surface of this large and exponentially complex interaction web, and every completed piece has value. Multi-omics will continue to be critical, bridging an important gap between taxa, products, and metabolic reactions. We aim to complete another piece, that involves *ecological relationships* between taxa. Microbial taxa have been shown to demonstrate a wide variety of ecological relationships, including cooperation [73, 74] and competition [75], that ultimately impact collective functionality of the ecosystem and macroscale properties [72]. We estimate these relationships for Control and ADHD datasets and report results; including relationships, communities, driver taxa (or ‘centroids’) of these communities, and taxa central to overall gut ecology. Results can offer guidance on potential taxa to target for further multi-omics or laboratory experiments. The ultimate goal is to increase depth of knowledge about connections between the influence of the gut microbiome on an NPD that impacts millions of individuals worldwide.

This work involves two parts, conducted on a publicly available, gender-matched dataset of 16S gut microbiome sequences. The first involves performing the same macroscale analyses currently prevalent in ADHD gut microbiome literature, to note how this dataset compares, as well as any new and interesting trends. Metrics will include alpha- and beta-diversity, Sparse Partial Least Squares Discriminant Analysis (sPLS-DA, [76]) to estimate Control and ADHD differentiation degree, various methods for differential abundance analysis [77–80] and Qiime [81] normalized abundance compositional profiles.

In the second part we estimate ecological relationships [82] within Control and ADHD gut microbiomes. We first use Microbial Co-occurrence Networks (MCNs, [83]) to estimate these relationships [84], and then perform cluster analysis using the Affinity Propagation (AP, [85]) algorithm to discover communities of mutually supporting taxa, as well as driver or ‘centroid’ nodes of these communities. Finally we perform centrality analysis using the Ablatio Triadum (ATria, [86]) algorithm, to estimate taxa most significant to the overall ecosystem.

## Materials and methods

We provide more details on the methods we use for analysis. Our entire downstream analysis pipeline has been built using Plugin-Based Microbiome Analysis (PluMA, [87]) version 2.0 and is available for download within its publicly available pipeline pool, (pipeline ADHD, available open-source at http://biorg.cs.fiu.edu/pluma/pipelines.html), along with processed sequence data. Source code for PluMA is available open source under the MIT software License at http://biorg.cs.fiu.edu/pluma. Supplementary material, including all alpha- and beta-diversity output, taxa plots, differential abundance output, networks, clusters, and central nodes are available in computer-readable TXT and CSV formats at http://biorg.cs.fiu.edu/pluma/ADHD.html.

### Cohort

Our study starts from a publicly available dataset (Accession Number: PRJNA656791) of raw gut microbiome samples (observational, case-control) from an undergraduate student population. Members of this population completed the Adult ADHD Self Report Scale (ASRS) and were classified as “Control” or “ADHD” following the published practice [88] of scoring at least 17 on one of the two subscales (inattention or hyperactivity). A subset of patients were then randomly selected from each group (32 Control, 29 ADHD) for gut microbiome sampling. Full sequencing details are provided in the BioProject description; 16S rRNA (V3-V4 region) sequencing was used, following steps corresponding to standard Illumina protocols [89]. We include details of the experiment that we were able to gather from both the BioProject and its referenced protocols [90–92] using STORMS [93] format in S1 Table. Each deidentified sample provides gender (NCIT:C17357, self-reported) and ADHD assessment (EFO:0007860) based on ASRS score (Control, ADHD Combined, ADHD Inattentive, or ADHD Hyperactivity) in its title. The project released 61 samples, but recommended excluding 3 based on outlier analysis (Mahalanobis distance, confidence level = 97.5%) leaving 58 samples: 30 Control and 28 ADHD, with 15 females in both groups. We summarize statistics in Table 3. Tetachoric correlation [94] revealed little to no association (-0.05) between gender and group classification (Control or ADHD).

**Table 3:** Cohort analysis.

Groups	Total No	Gender	Total No	Inattentiveness	Hyperactivity	Combined
				ASRS Scores ± SD		
Control	30	Female	15	11.2 ± 4.3	9.6 ± 4.9	20.8 ± 7.2
		Male	15	9.8 ± 3.4	9.9 ± 4.4	19.7 ± 6.8
ADHD	28	Female	15	22.6 ± 6.8	19.5 ± 5.3	42.1 ± 10.1
		Male	13	18.2 ± 3.8	17.3 ± 4.7	35.5 ± 6.1

Cohort analysis. Samples are from an undergraduate student population.

The study used ASRS as its method for case-control classification, and much of our analyses apply protocols which assume discrete classification schemes for samples. It is important to note however, that while the ASRS can be a useful screening tool for ADHD [95] and has been shown to achieve accuracy rates above 90% [96], it in the end does not offer an official diagnosis. Therefore, an immediate next step must involve a transition to continuous analysis protocols, operating on the ASRS scores (and possibly subscales as well) as purely continuous data. We present these results as preliminary work, with the expectation of an immediate segue into this continuous analysis as future work. We elaborate on these points in the discussion.

Sequences were trimmed using Trimmomatic-0.33 [97] then assembled into a complete amplicon sequence using SeqPrep [98]. Chimeric amplicon sequences were removed using USearch 6.1 [99] in reference mode against a curated RDP database [100], along with all sequences with length under 320bp. Finally for each sample, a set of 50000 sequences were randomly sampled to reduce uneven sampling bias. To establish an initial set of Operational Taxonomic Units (OTUs) we took these sequences and compiled, clustered, and analyzed them using Qiime 1.9.1 [81] open reference clustering (UClust, [99]) with a similarity threshold of 97% (GreenGenes version 13.8 reference database [101]). Each of these packages are available as PluMA plugins. Finally, we removed all singletons. **S2 Table** shows the average number of sequences retained in each sample after each preprocessing step.

### Ethics Statement

As mentioned, we commenced this study from a publicly available dataset that was released in fully deidentified form and no master key was provided, blocking any possible route for tracing a sample back to an individual. IRB consent was therefore verbally waived for our study, by the FIU Office of Research Integrity.

### Part I. Traditional macroscale analyses

We first perform macroscale analyses on this ADHD dataset that have been performed on other ADHD datasets, compare and contrast our results with those in the literature, and take note of any new and interesting observations.

#### Diversity analysis

Alpha- and beta-diversity plots were constructed using Qiime (version 1.9.1). Input was provided in the form of raw abundances, sampled at a depth of 15000. For alpha-diversity, we used default metrics: unique taxa count, Chao1 [102], and PD_whole_tree (phylogenetic diversity), with default parameters. We confirm results using a non-parametric *t*-test (999 Monte Carlo permutations and Bonferroni [103] correction for p-values). For beta-diversity, we plot unweighted and weighted Unifrac distance [104], confirming results through an ADONIS [105] statistical test on their respective distance matrices (999 permutations).

#### Differential abundance analysis

We conduct this using two methods, that produce different types of classification. The first uses Sparse Partial Least Squares Discriminant Analysis (sPLS-DA, [76]), a sparse version of the Partial Least Squares (PLS, [106]) method (confidence = 95%), as a supervised dimension reduction method for determining differentiation degree between Control and ADHD samples with respect to taxa abundance [107]. We use centered log-ratio (CLR, [108]) transformed abundances to map to an unbound space and reduce potential compositional effects. The second attempts to uncover specific microbial taxa that are elevated/reduced in Control and ADHD samples. For this, we take a consensus approach [109] using four methods: (1) Linear discriminant analysis Effect Size (LEfSe,[77]) (*p* ≤ 0.05, LDA effect size > 2) with Bonferroni [103] correction, (2) DESeq2 [78] with Benjamini-Hochberg [110] False Discovery Rate (FDR) correction (*p* ≤ 0.05), ANOVA-like Differential Gene Expression Analysis of Single-Organism and Meta-RNA-Seq (ALDEx2, [79]) (*p* ≤ 0.05, also with Benjamini-Hochberg correction), and Analysis of Compositions of Microbiomes with Bias Correction (ANCOM-BC, [80]) (*p* ≤ 0.05, Holm-Bonferroni [111] correction). Taxa were classified at the lowest possible taxonomic level.

#### Taxa plots

We use Qiime 1.9.1 [81] to generate taxa bar graphs, producing one bar per sample broken down by taxa percentages (this directly corresponds to our normalized data). We produce taxa plots at all levels of the taxonomic tree (from phylum to genus), plus one at the lowest possible level of classification.

### Part II: Ecological relationships

#### Co-occurrence network analysis

We computed correlations based on log-ratio transformed taxa absolute abundances using SparCC [112] (*p* ≤ 0.01) using 100 permutations and 5 iterations, and built Microbial Co-occurrence Networks (MCNs, [83]) using taxa as nodes and correlations as edges. MCNs were visualized using Cytoscape (version 3.9.1, [113]) with layout produced by Fruchterman-Reingold [114].

#### Clustering

MCNs were clustered using Affinity Propagation (AP, [85]) with a damping coefficient of 0.5 and 200 convergence iterations. AP has been shown to operate efficiently and successfully on signed and weighted biological networks without requiring an initial cluster count estimate, and additionally computes the most representative or *centroid* node for each cluster.

#### Centrality analysis

We use Ablatio Triadium (ATria, [86]) for evaluating the importance, or *centrality*, of taxa in our MCNs. ATria computes centrality for signed and weighted networks through a modified economic payment model [115] that calculates the influence of a node on all other nodes. ATria provides an alternative perspective by considering relationships (not relative abundance) when computing centrality, and unlike LEfSe does not compare sample sets. ATria produces a ranked list of important taxa and runs iteratively; once a taxon is found as central, ATria removes this taxon and its dependencies using social network theory [116]. Then it runs again to produce the next most important taxon, repeating until no edges are left. Taxa not found as important are simply not ranked.

We analyze these ecological relationships at all taxonomic levels starting from phylum. We first observe the upper three levels (phylum, class, and order) for an overview of relationships between consistently abundant taxa and an observation of general trends and behaviors. We then move to the lower three levels (family, genus and lowest possible classification level) which provide a finer level of granularity and enough taxa to perform meaningful community analyses, facilitating a vision of the key players within these dynamics. The lowest possible classification level will attempt to classify at a species level, but because of the lack of variability in the 16S gene at the species level only a limited number of taxa will be classifiable at an appropriate level of confidence. When species-level classification is not possible, the next highest level (genus or family) will be used. To be clear, these networks are based on correlations, which does not imply causation. Results from our analyses should therefore be interpreted as providing guidance and potential targets for future downstream (i.e. pathway or multi-omics) analysis, that ultimately will require lab experimentation for full verification.

#### Prevalence filtering

Once we venture past macroscale community metrics (alpha- and beta-diversity), we apply a prevalence filter to remove scarce taxa and restrict our analysis to core microbiome members of both groups (Control and ADHD) by adopting a prevalence threshold of 50% [117, 118]. By restricting our analysis to taxa that are not scarce, our sample size (30 and 28, 1.07 control to case ratio) achieves a power size of 88% given a two-sided confidence interval of 95% when comparing specific taxa between Control and ADHD sets (computed using EpiInfo version 7.2.5, STATCALC package), critical especially for differential abundance analysis. Additionally for co-occurrence networks, a prevalence threshold is important for avoiding spurious clusters of correlated scarce taxa that are simply not appearing across most samples, and 50% is estimated to be a moderate to conservative threshold [119]. A survey across multiple correlation algorithms (including SparCC) also found a performance decline (defined as the ratio of true to false positives) once sparsity (the percentage of zeroes in correlated taxa) exceeded 50% [120]. **S2 Table** shows that this prevalence filter still maintains 91% of sequence counts on average per sample.

With this prevalence filter the final set of taxa will differ slightly for Control and ADHD, as some taxa will meet the prevalence threshold in only one of the two groups. This will create bias in differential abundance calculations, as these taxa will have an artificial zero abundance in one group but not the other. To avoid this bias, we keep taxa that meet the prevalence threshold in one of the two groups in both groups for all differential abundance calculations (SPLS-DA, LEfSe, DESeq, ALDEx2 and ANCOM-BC). For all other methods that do not perform differential abundance calculations between the two groups (taxa plots and co-occurrence networks), we use the prevalence filter as defined above.

## Results

### Part I. Traditional macroscale analyses

#### Diversity

**Fig 1:**
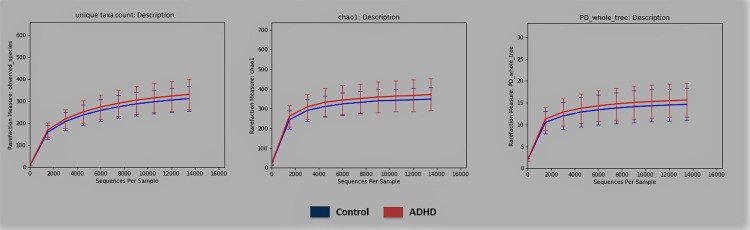
Alpha-diversity. Alpha-diversity of Control (blue) and ADHD (red) samples using (in order) the count of unique taxa, Chao1 richness [102], and phylogenetic diversity, with error bars.

**Fig 2:**
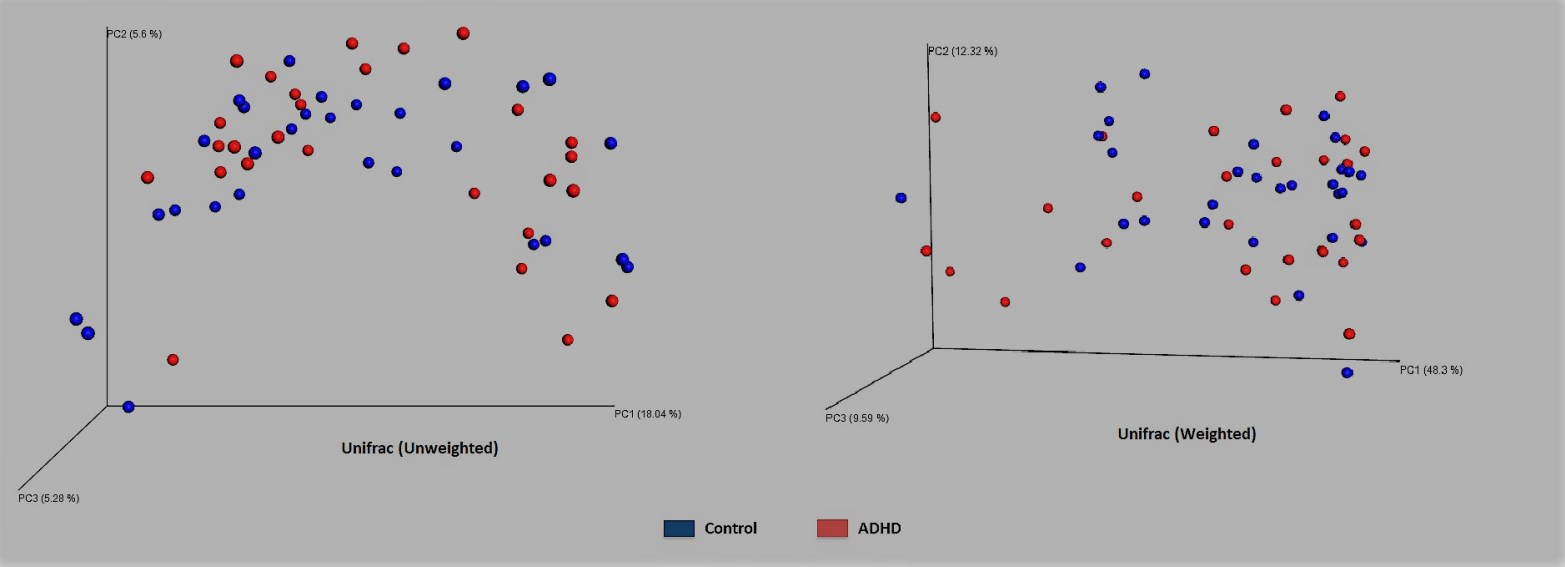
Beta-diversity. Beta-diversity of Control (blue) and ADHD (red) samples computed using unweighted and weighted Unifrac [104] distance.

Qiime [81] alpha- and beta-diversity results produced no conclusive differences between ADHD and Control. Fig 1 shows no Alpha diversity difference within error bars for ADHD using all three metrics: unique taxa count, Chao1 [102], and PD_whole_tree (phylogenetic diversity), which was later confirmed by a non-parameteric *t*-test (respective *t* = -1.14, -1.17, -0.967; *p*-values: 0.2664, 0.246, 0.309). Beta-diversity with unweighted and weighted Unifrac [104] distance also shows no separation (Fig 2), confirmed by *p*-values computed from an ADONIS [105] statistical test (respective *F* = 0.847, 0.581; *p*-values = 0.707, 0.671 respectively). This lack of alpha- and beta- diversity differences matches several results from other datasets [61, 62, 66].

#### Differential abundance

**Fig 3:**
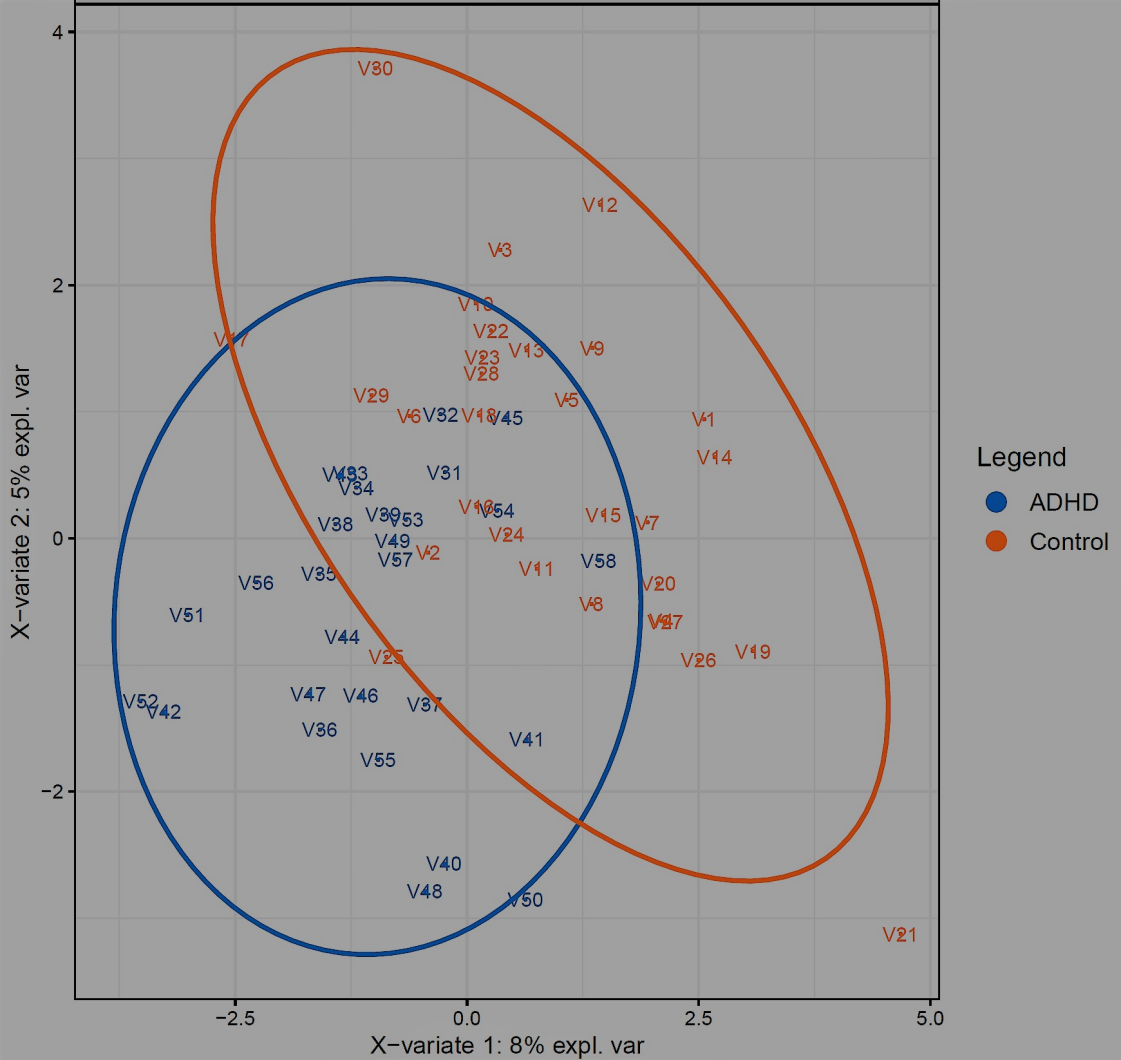
Differential analysis. Results of running sPLS-DA [76] on microbiome abundance data (ellipse confidence level 95%), comparing Control (orange) and ADHD (blue) groups.

One differential abundance technique involves dimensionality reduction, attempting to determine differentiation degree between datasets, by accounting for all variables in each set [121] but displaying in a reduced dimensional space using principal component analysis. Unsupervised and supervised approaches can be used, with supervised having prior sample classification knowledge (i.e., Control or ADHD). When applying a CLR transform to our data in Fig 3 (ellipse confidence level = 95%), we see that even our supervised method Sparse Partial Least Squares Discriminant Analysis (sPLS-DA, [76]) is unable to convincingly differentiate Control vs. ADHD. Table 4 confirms this, as classification error rates of the two principal components for general Control vs. ADHD range from 40 to 50%. This is even with a supervised method, which needs to be taken with a grain of salt anyway, as supervised methods have *a priori* sample category knowledge and have sometimes been shown to differentiate completely random data [107].

**Table 4:** Principal component error rates.

Principal Component	Largest Predicted Score	Distance to Centroid (Euclidean)	Distance to Centroid (Mahalanobis)
1	0.476	0.481	0.481
2	0.453	0.457	0.452

Classification error rates of the top two principal components in Fig 3.

Techniques for dimensionality reduction like sPLS-DA estimate the degree of differentiation between two sets (e.g., Control and ADHD). For more targetted future lab studies, it may also be useful to estimate specific taxa that are elevated in one set of samples (e.g., Control) compared to another set of samples (e.g., ADHD). As differential abundance techniques have been shown to vary widely in their results [109], we use four techniques (LEfSe [77], DESeq2 [78], ALDEx2 [79] and ANCOM-BC [80]) with a range of different correction mechanisms for their *p*-values (Bonferroni [103], Benjamini-Hochberg [110], and Holm-Bonferroni [111]) though keep the same *p*-value threshold of 0.05.

**Fig 4:**
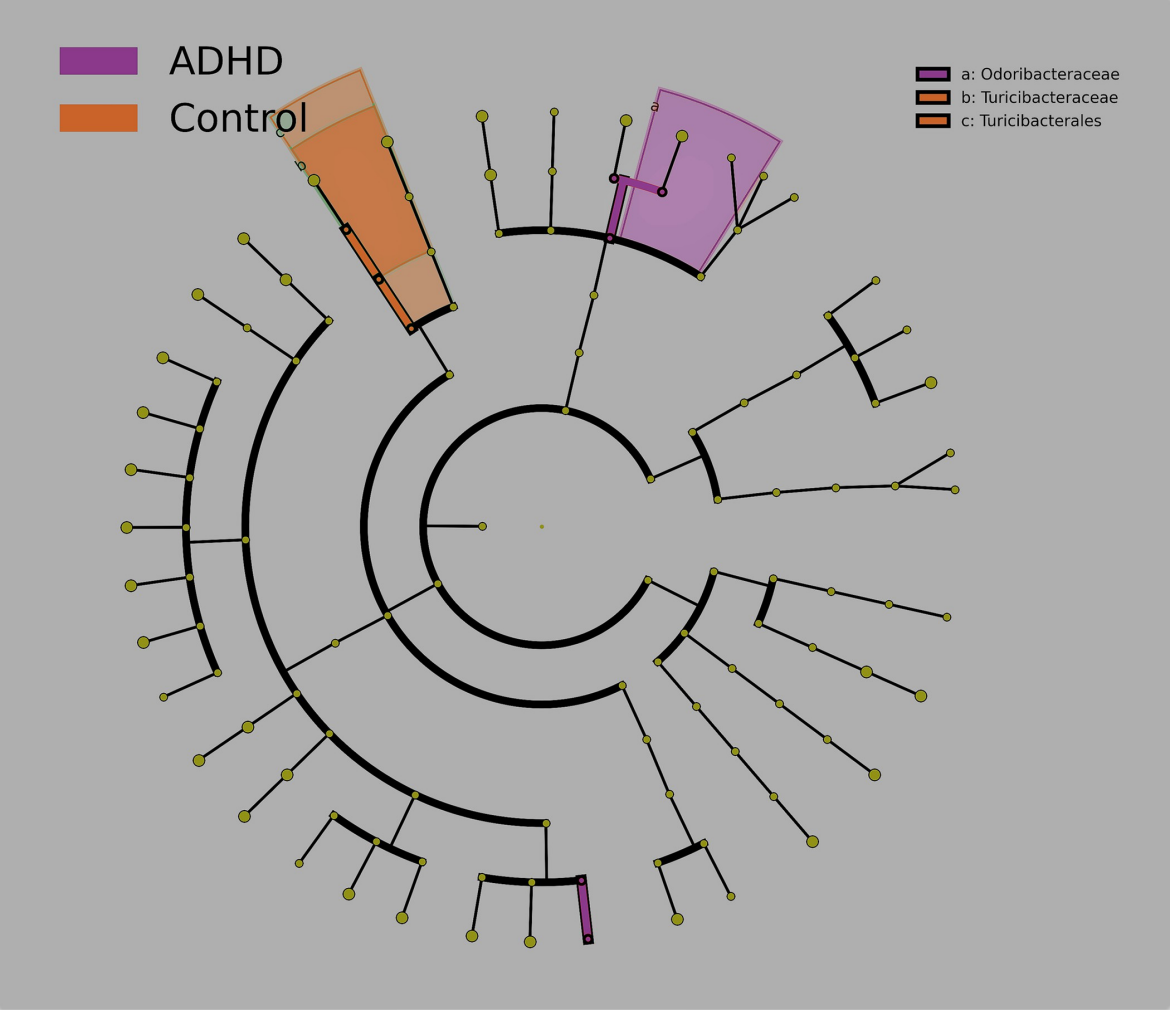
Differential abundance (Cladogram). Taxa reported by LEfSe as elevated for Control (orange) and ADHD (purple) groups, produced by LEfSe [77] (*p ≤* 0.05, Bonferroni correction). LEfSe-reported taxa are plotted on a cladogram, with each concentric circle representing a phylogenetic classification level (innermost = phylum). Shared areas represent distinctive regions of the phylogenetic tree.

**Fig 5:**
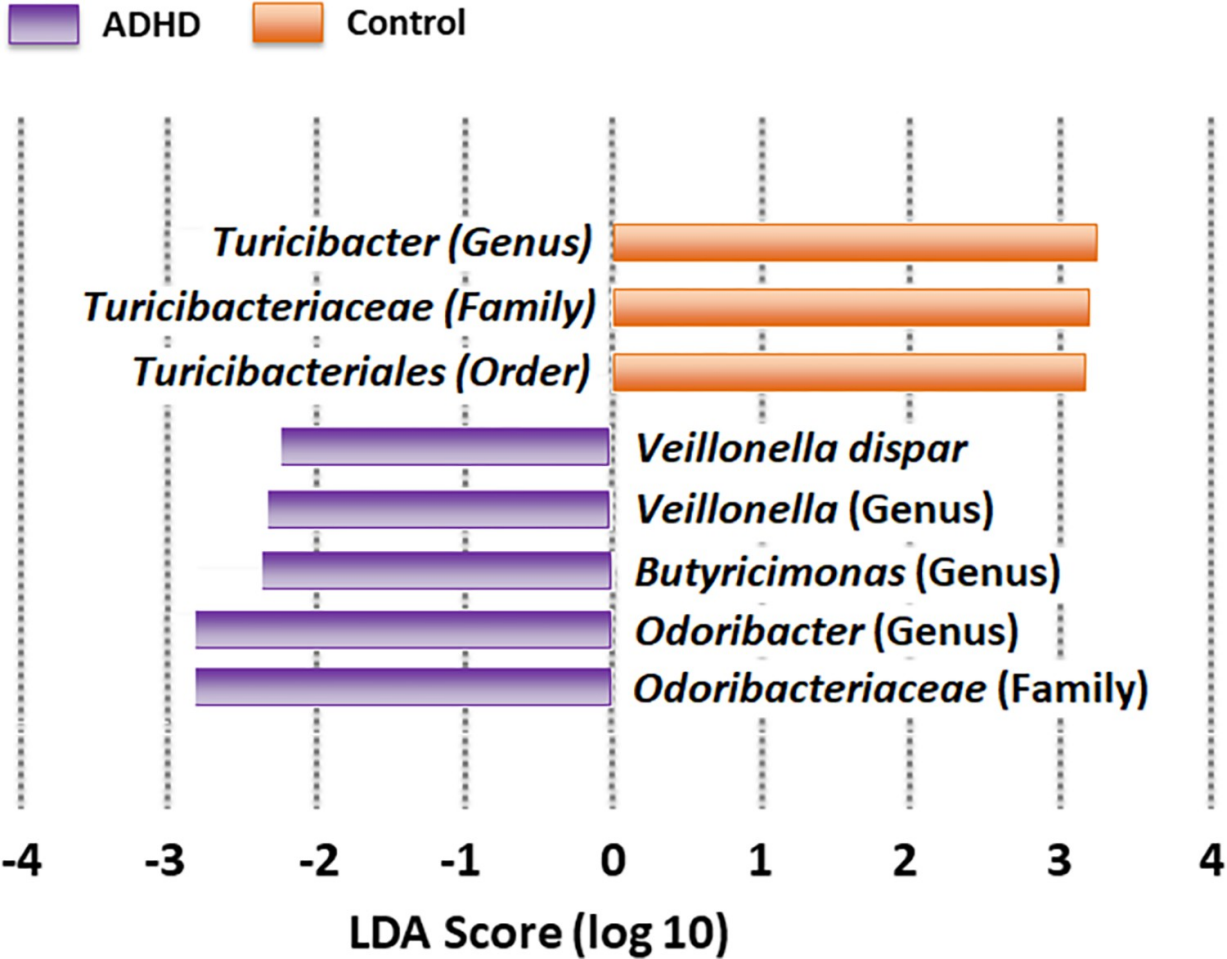
Differential abundance (Bar graph). LEfSe-reported taxa ordered by Linear Discriminant Analysis (LDA, [122]). A higher magnitude indicates more reliable differentiation.

We first show results for LEfSe (*p ≤* 0.05, Bonferroni correction) both as a cladogram (Fig 4) and a bar graph (Fig 5). LEfSe has identified orange taxa as elevated for Control, and purple taxa as elevated for ADHD. Taxa produced by LEfSe will be classified at the lowest taxonomic level possible.

The cladogram (Fig 4) shows these taxa on the phylogenetic tree, highlighting those closely related. Fig 4 shows one Bacteroidetes family (*Odoribacteriaceae*) distinguishing ADHD, while Firmicutes (family *Turicibacteriaceae* and its order Turicibacterales) distinguishing Control. The bar graph (Fig 5) uses Linear Discriminant Analysis (LDA, [122]) to order by differentiation degree, expanding to include genera and species. ADHD continues to be predominated by Bacteroidetes taxa, again including *Odoribacteriaceae* and its genera *Odoribacter* and *Butyricimonas*, supporting earlier claims of *Odoribacteriaceae* as ADHD-elevated [68]. *Veillonella* and member *V*. *dispar* were also reported as elevated in ADHD. Control continues to be predominated by Firmicutes taxa (now including genus *Turicibacter*).

We also run three other methods: DESeq2 [78] and ALDEx2 [79] (both with Benjamini-Hochberg [110] corrected *p* ≤ 0.05, and ANCOM-BC [80] with a Holm-Bonferroni corrected *p* ≤ 0.05. Table 5 shows our results. In addition to *p*-values we include the applicable effect size–Linear Discriminant Analysis (*LDA*) for LEfSe, Log-Fold Change (LFC) for DESeq2, and Effect Size (EFF) for ALDEx2, computed as the ratio between the group difference and the larger of the two internal group variations. Note ANCOM-BC did not report any differentially abundant taxa. *Turicibacter* and *Butyricimonas* (in **bold**) are the only genera reported more than one of these methods, and *Turicibacter* was actually repoted by three (LEfSe, DESeq2 and ALDEx2).

**Table 5:** Taxa reported by various differential abundance methods throughout our analysis.

Taxonomic Level	Taxon	Algorithm	Control/ADHD	Effect Size and *p*-value
*Family*	*Clostridiaceae 1*	ALDEx2	Control	*EFF =* 7.90, *p =* 0.016
*Genus*	*Coprobacillus*	DESeq2	ADHD	*LFC* = 2.00, *p* = 0.044
*Genus*	*Odoribacter*	LEfSe	ADHD	*LDA* = 2.68, *p* = 0.027
***Genus***	***Turicibacter***	**LEfSe**	**Control**	***LDA* = 3.04, *p* = 0.028**
		**DESeq2**		***LFC* = 2.12, *p* = 0.007**
		**ALDEx2**		***EFF* = 1.75, *p* = 0.033**
*Genus*	*Haemophilus parainfluenzae*	DESeq2	Control	*LFC* = 0.572, *p* = 0.042
***Genus***	***Butyricimonas***	**LEfSe**	**ADHD**	***LDA* = 2.37, *p* = 0.019**
		**ALDEx2**		***EFF* = 3.15, *p* = 0.032**
*Lowest Possible*	*V*. *dispar*	LEfSe	ADHD	*LDA* = 2.31, *p* = 0.039

Taxa reported as elevated by various differential abundance methods, when comparing Control vs ADHD, and further broken down by subscale. *LDA* = Linear Discriminant Analysis effect size (for LEfSe), *LFC* = Log Fold Change (for DESeq2), EFF = Effect Size (for ALDEx2).

Genus *Odoribacter* has also been previously reported as ADHD-elevated [68]. Although species *H*. *parainfluenzae* has not been observed as Control-elevated in any ADHD gut microbiome studies, its genus (*Haemophilus*) has [63]. This same study [68] found family *Veillonellaceae* as Control-elevated and member species *V*. *parvula* as differentially abundant in ADHD. Our LEfSe analysis found *Veillonellaceae* members *Veillonella* and *V*. *dispar* as differentially abundant in ADHD samples. Although *Turicibacter* has never previously reported as a elevated or reduced in any ADHD gut microbiome study, it has been reported in one involving depression in mice [42]. Metabolically in mice, *Turicibacter* signals the gut to produce serotonin (5-HT) [123], which influences ER [124]. Both ADHD and depression are characterized by ER neurocircuitry deficiencies. LEfSe did not report any EF-associated taxa. This may be largely because EF is more strongly regulated by dopamine [124], for which the gut only produces roughly 50% [125], compared to 90% of 5-HT [29].

#### Taxa plots

**Fig 6:**
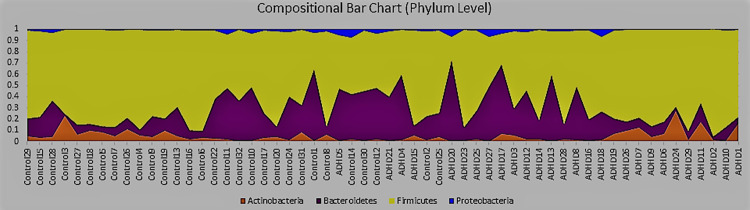
Taxa plots, phylum level. Plots of taxa relative abundance for each subject, generated using Qiime [81], conducted at the phylum level. Subjects are ordered by increasing Adult ADHD Self Report Scale (ASRS) score, with the y-axis representing relative abundance.

Taxa bar plots visualize taxa relative abundances [126]. We generated these at all phylogenetic tree levels beginning with phylum (Fig 6). Samples on the *x*-axis are ordered by increasing ASRS score, and the *y*-axis represents relative abundance.

A typical gut microbiome profile [127] is observed, dominated by Firmicutes and Bacteroidetes phyla, followed by Actinobacteria and Proteobacteria. Control has slightly elevated Firmicutes (70–66%), mirroring an earlier study [66] that importantly [128] also sequenced the same 16S V3-V4 region. Slightly contrary to this same study, which reported this difference to be largely occupied by an ADHD Actinobacteria increase, ours was mostly occupied by an ADHD Bacteroidetes increase (from 22% to 25%). Yet Actinobacteria remains mysterious in Fig 6, elevated at very high ASRS scores, but also at very low scores. Bacteroidetes and Proteobacteria also appear reduced at these same extremes. These seemingly contradictory results create challenges in drawing meaningful conclusions with respect to role(s) played by these phyla. Yet they capture our interest, especially given the earlier reported anomalous behavior of an Actinobacteria genus, *Bifidobacterium*, at high and low ASRS-IV scores [70].

**Fig 7:**
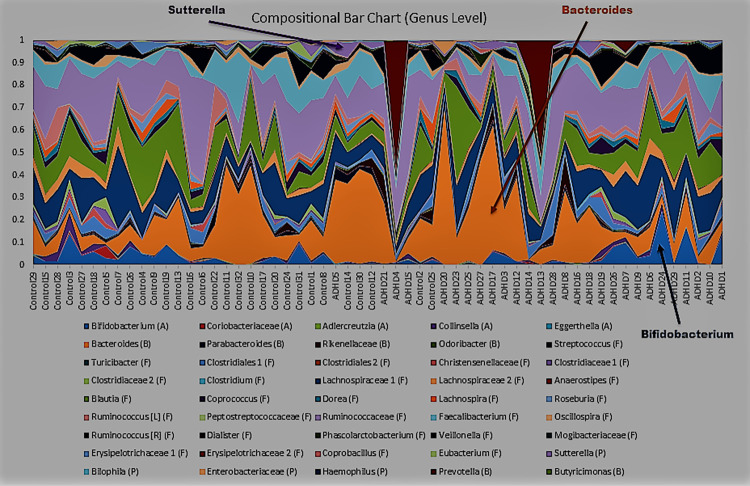
Taxa plots, genus level. Plots of taxa relative abundance for each subject, generated using Qiime [81], conducted at the genus level. Subjects are ordered by increasing Adult ADHD Self Report Scale (ASRS) score, with the y-axis representing relative abundance.

Class and order levels produced bar charts similar to Fig 6; we include these as S1 and S2 Figs. Levels below order often had too many taxa to clearly view dynamics. We include the genus level (Fig 7), family and species as S3 and S4 Figs), as the genus level includes *Bifidobacterium*. And indeed, it turns out, *Bifidobacterium* (blue, bottom) has elevated abundances high and low ASRS scores, appearing most responsible for this same behavior in its phylum Actinobacteria (Fig 6). *Bacteroides* (orange, middle) is a highly abundant genus that also mirrors the behavior of its phylum (Bacteroidetes, Fig 6), increasing in the middle and decreasing at extremes. Proteobacteria is more difficult to observe given its low relative abundance (1–2%), though genus *Sutterella* (lilac, top) also appears to follow this trend. These three observations are verified in S5–S7 Figs.

This is not the first time these taxa have generated interest. Many Actinobacteria, and especially *Bifidobacterium*, have been used as probiotics and are considered elements of a healthy gut [129–134]. As discussed, genus *Bacteroides* and its family *Bacteroidaceae*, as well as several member species, have been reported differentially abundant in ADHD [59, 60, 65, 68]; some elevated, others reduced. Some have argued *Bacteroides* to be the most important "window" to understanding the human gut [135]. Species *Sutterella stercoricanis* was also reported as ADHD elevated [60]. These same taxa make multiple appearances in studies involving other NPDs as well. *Bacteoridaceae* was reported as the most differentially abundant LEfSe Major Depressive Disorder (MDD) taxon in one study [45]. Another reported elevated *Bacteroides* and reduced *Bifidobacterium* in anxiety [136]. *Sutterella* is elevated in Autism Spectrum Disorder (ASD) [137], a condition with so much symptomatic overlap with ADHD that an ASD+ADHD phenotype has been established [138].

#### Discussion

These analyses produced a few interesting preliminary observations, but once again their birds-eye view limited the depth we could pursue. Our taxa plots showed a perfect example: even though there was a visible trend between ASRS score and *Bifidobacterium*, *Bacteroides*, and *Sutterella* abundances, no definitive conclusions could be observed. Fundamentally macroscale behaviors emerge from microscale interactions. We attempt to unlock some of these mysteries by now exploring *ecological relationships*.

Microbial ecological relationships take many forms. They can be positive or negative, mutual (cooperation [73, 74] or competition [75]) or one-way (commensalism [139] or amensalism [140]). In particular, two-way relationships (cooperation and competition) can be approximated using correlations [84]. We use SparCC [112] to compute correlations, which has advantages in reducing compositional effects within relative abundances. We build Microbial Co-occurrence Networks (MCNs, [83]) using taxa as nodes and SparCC correlations as edges, and perform community detection on these networks using the clustering algorithm Affinity Propagation (AP, [85]). Finally, we use Ablatio Triadium (ATria, [86]) as a centrality algorithm to produce a ranked list of important taxa in each MCN. ATria is specifically designed for signed and weighted networks, incorporating both social network [117] and economic theory [116] in its calculations. It is also iterative, removing dependencies of a central node before computing the next most central.

When computing correlations, we use a *p*-value threshold of 0.01, the generally recommended value for disease studies [141], to increase the confidence of our results, as the historically accepted threshold of 0.05 has come under recent question [142, 143]. Because our study is exploratory and meant to guide future experimentation, we chose to err on the side of false positives as opposed to false negatives, and increase the strength of the threshold as opposed to correcting the *p*-value. Our p-value threshold of 0.01 produced correlations with magnitude moderate (0.4, [144]) or stronger more than 95% of the time (279 out of 293 total over all MCNs). We therefore report all of these candidate relationships in our MCNs, each of which can be subsequently experimentally verified in future studies.

During our analyses we sometimes use “cooperation” to refer to a positive SparCC correlation and “competition” when referring to a negative. We emphasize, however, that correlations are an *estimate* of ecological relationships, that ultimately require further downstream analysis (multi-omics and experimental verification) before establishing official conclusions. With the underlying web of interactions being exponentially complex and large-scale laboratory experiments potentially costly, our results can provide guidance regarding target taxa and avenues to pursue.

### Part II. Ecological relationships

#### Upper levels: Phylum, class, and order

**Fig 8:**
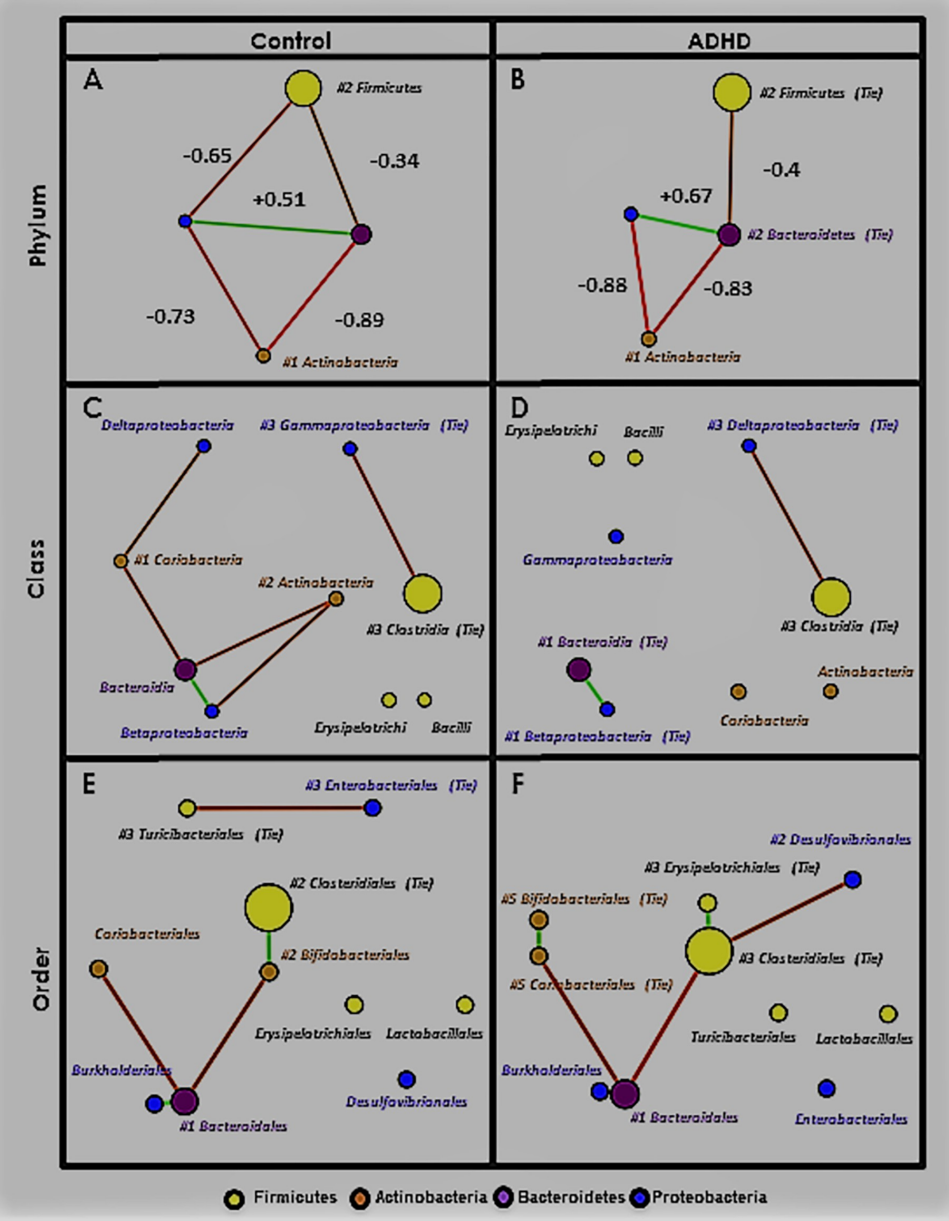
Upper-level Microbial Co-occurrence Networks (MCNs). MCNs at the phylum (A), class (B), and order (C) taxonomic levels, visualized using Cytoscape [57], and oriented by Fruchterman-Reingold [58]. Nodes represent taxa, colored by phylum (yellow = Firmicutes, purple = Bacteroidetes, brown = Actinobacteria, blue = Proteobacteria) with size directionally proportional to abundance. The co-occurrences are distinguished by those that co-habit (green edges) and co-avoid (red edges). SparCC [112] correlation (*p*≤0.01) was used as edge weight and also the parameter for Fruchterman-Reingold when determining edge length (larger = closer). SparCC correlations are shown at the phylum level. All taxa found as important by ATria are denoted by a pound sign (#) followed by its rank (ties indicated).

Fig 8 shows MCNs at the phylum (Fig 8(A) and 8(B)), class (Fig 8(C) and 8(D)), and order (Fig 8(E) and 8(F)) levels. Taxa (nodes) in all MCNs are colored by phylum (legend at the bottom of Fig 8). Node size is proportional to relative abundance (larger = higher). Correlation (edge) color represents sign; green indicates positive (est. cooperation) and red indicates negative (est. competition). Edge thickness is proportional to correlation magnitude (thicker = stronger). Networks are visualized using the Fruchterman-Reingold algorithm [114], which spatially orients nodes based on edge weight (closer = more positive). Nodes are labeled with their taxon and provided with ATria centrality ranking if found important (format: *#rank*, T = Tie). At the phylum level only (Fig 8(A) and 8(B)), we label each edge with its correlation value. Phylum-level MCNs (Fig 8(A) and 8(B)) show SparCC appears to handle compositional effects well, as despite collectively encompassing about 95% of both populations, Firmicutes and Bacteroidetes are only weakly negatively correlated.

Table 6 shows every correlation in all three MCNs, and its sign, + (green) or–(red). Correlations that appear only in Control are highlighted orange, only in ADHD highlighted purple, and in both highlighted grey. Correlations at each taxonomic level are grouped by their next highest level classification; for example in row 1: phyla Actinobacteria and Bacteroidetes were negatively correlated in both phylum-level MCNs (Fig 8(A) and 8(B)), member classes Actinobacteria and Bacteroidia were negatively correlated only in Control (Fig 8(C)), as were member orders Bifidobacteriales and Bacteroidales (Fig 8(E)). White, italicized correlations were not present in either MCN, but a correlation among descendants was; for example in row 3: phyla Actinobacteria and Firmicutes were not correlated in either MCN, nor were member classes Actinobacteria and Clostridia, but member orders Bifidobacteriales and Clostridiales were positively correlated in Control (Fig 8(E)).

**Table 6:** Upper-level taxa correlations.

Phylum		Class		Order	
Actinobacteria-Bacteroidetes	-	Actinobacteria-Bacteroidia	-	Bifdobacteriales-Bacteroidales	-
		Coriobacteria-Bacteroidia	-	Coriobacteriales-Bacteroidales	-
Actinobacteria-Proteobacteria	-	Actinobacteria-Betaproteobacteria	-		
		Coriobacteria-Deltaproteobacteria	-		
*Actinobacteria-Firmicutes*		*Actinobacteria-Clostridia*		Bifidobacteriales-Clostridales	+
Proteobacteria-Firmicutes	-	*Gammaproteobacteria-Bacilli*		Enterobacteriales-Turicibacteriales	-
		Gammaproteobacteria-Clostridia	-		
		Deltaproteobacteria-Clostridia	-	Desulfovibrionales-Clostridiales	-
Proteobacteria-Bacteroidetes	+	Betaproteobacteria-Bacteroidia	+	Burkholderiales-Bacteroidales	+
Bacteroidetes-Firmicutes	-	*Bacteroida-Clostridia*		Bacteroidales-Clostridiales	-

Upper level taxa correlations (*p* ≤ 0.01), grouped by taxonomic classification. Orange = only found in Control, purple = only found in ADHD, grey = found in both. +(green) = positive correlation, -(red) = negative correlation.

Table 7 shows collective ATria results, similarly grouped. At each level, taxa found equally important in both MCNs are highlighted grey; taxa found more important in Control light orange, and only important in Control dark orange (analogous case for ADHD and purple). Taxa ranked as first or tied for first in either MCN are **bold**.

**Table 7:** Upper-level ATria rankings.

Phylum	Class	Order
**Actinobacteria (#1/#1)**	Actinobacteria (#2/NR)	Bifidobacteriales (#T2/T5)
	**Coriobacteria (#1/NR)**	Coriobacteriales (NR/#T5)
Bacteroidetes (NR/#T2)	**Bacteroidia (NR/#T1)**	**Bacteroidales (#1/#1)**
Firmicutes (#2/#T2)	*Bacilli (NR/NR)*	Turicibacteriales (#T3/NR)
	Clostridia (#T3/#T3)	Clostridiales (#T2/#T3)
	*Erysipelotrichia (NR/NR)*	Erysipelotrichiales (NR/#T3)
*Proteobacteria (NR/NR)*	**Betaproteobacteria (NR/#T1)**	
	Deltaproteobacteria (NR/#T3)	Desulfovibrionales (NR/#2)
	Gammaproteobacteria (#T3/NR)	Enterobacteriales (#T3/NR)

Upper-level ATria results, grouped by taxonomic classification. Dark orange = only ranked in Control, dark purple = only ranked in ADHD, light orange = higher ranked in Control, light purple = higher ranked in ADHD, grey = evenly ranked. **Bold** taxa are ranked #1.

Taxa bar plots are mirrored here: ADHD showed elevated Bacteroidetes at the expense of Firmicutes, and these taxa are negatively correlated in both MCNs (Fig 8(A) and 8(B)). But while Firmicutes and Bacteroidetes dominate both populations (largest nodes, Fig 8(A) and 8(B)) as is typical in the gut microbiome [127], SparCC and ATria estimate a far less abundant phylum, Actinobacteria (roughly 4% of both populations), as most important to their overall gut ecology. In both MCNs (Fig 8(A) and 8(B)), phylum Actinobacteria has the strongest negative correlations and ATria ranks it first (Table 7).

We make three more observations at these upper taxonomic levels, that we keep in mind when moving to the lower:

*(A) A core Proteobacteria-Bacteroidetes positive correlation (est*. *cooperation) forms*. Table 6 shows this, with Proteobacteria and Bacteroidetes (the only positive correlation in either phylum-level MCN), member classes Betaproteobacteria and Bacteroidia, and member orders Burkholderiales and Bacteroidiales.

*(B) In Control*, *taxa in (A) have more negative edges with Actinobacteria (est*. *competition)*, *especially order Bifidobacteriales*. The highest magnitude negative edges in both phylum-level MCNs (Fig 8(A) and 8(B)) involve Actinobacteria with Proteobacteria and Bacteroidetes. Yet while the two consistently dependent Actinobacteria classes (Actinobacteria and Coriobacteria) continue this same dynamic with Bacteroidia (Bacteroidetes) and Betaproteobacteria (Proteobacteria) in Control (Fig 8(C) and Table 6), they are completely disconnected in ADHD (Fig 8(D)). Worth noting, this is despite their average relative abundance being nearly the same in Control/ADHD: Coriobacteria 1.5/1.1%, and Actinobacteria 3.2/3.7%. Further, ATria ranks Actinobacteria and Coriobacteria as the top two Control taxa (Table 7). In ADHD, Bacteroidia and Betaproteobacteria are the top two (Table 7), and the MCN shows no negative edges (est. competition) at all involving these taxa (Fig 8(D)).

The order level reveals Bifidobacteriales (Actinobacteria) may be more responsible for this difference than Coriobacteriales (Coriobacteria). While Bifidobacteriales and Coriobacteriales both continue their negative correlations with Bacteroidales (Bacteroidia) in Control, only Coriobacteriales does in ADHD. Table 6 actually shows all edges involving Bifidobacteriales to be exclusive to Control, now including a positive correlation with Clostridiales (the most abundant Firmicute). An increased participation of order Bifidobacteriales thus emerges as a distinguishing feature of Control, which is further supported by ATria (Table 7), which ranks Bifidobacteriales higher (tied for second) in Control, and Coriobacteriales only in ADHD.

*(C) A shift in Firmicutes-Proteobacteria dynamics*. This begins immediately at the phylum level (Fig 8(A)) with Control having a negative correlation (-0.65) that is absent in ADHD (Fig 8(B)). The most abundant Firmicute class (Clostridia) is negatively correlated with different Proteobacteria classes; Gammaproteobacteria in Control (Fig 8(C)), Deltaproteobacteria in ADHD (Fig 8(D)), and the latter continues at the order level (Fig 8(F)) with Clostridiales (Clostridia) and Desulfovibrionales (Deltaproteobacteria). In Control (Fig 8(E)), a negative correlation emerges between order Enterobacteriales (class Gammaproteobacteria) and LEfSe-reported order Turicibacteriales (class Bacilli).

#### Summary

Upper-level analysis revealed increased Actinobacteria participation in Control gut ecology, especially order Bifidobacteriales. Much of this involved negative correlations with a core of positively correlated Bacteroidetes (order Bacteroidales) and Proteobacteria (order Burkholderiales). Recalling our taxa plots and anomalous behavior involving genera *Bifidobacterium* (Bifidobacteriales), *Bacteroides* (Bacteroidales), and *Sutterella* (Burkholderiales), we are now interested in exploring these dynamics at lower taxonomic levels. We will continue to observe Firmicutes-Proteobacteria dynamics, as despite a still unclear picture, a clear distinction is shown between Control and ADHD.

#### Lower levels: Family, genus, and lowest possible

**Fig 9:**
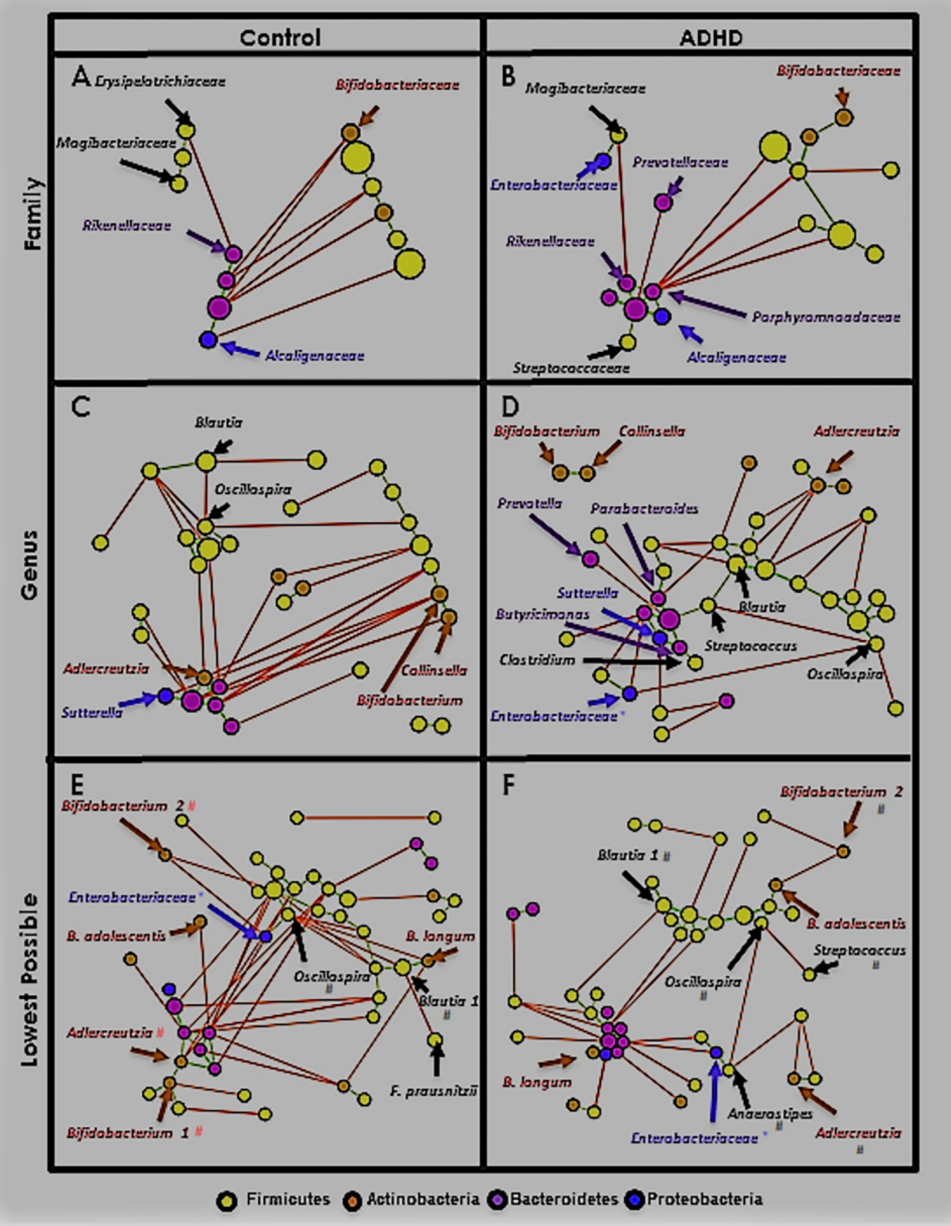
Lower-level MCNs. MCNs at the family (A), genus (B), and species (C) taxonomic levels. Network visual properties, including node and edge size, color, and orientation, are the same as Fig 8. Taxa noted throughout our analyses are labeled (* = family-level taxon, # = genus-level taxon).

Fig 9 shows Control and ADHD MCNs at family (Fig 9(A) and 9(B)), genus (Fig 9(C) and 9(D)), and lowest possible taxonomic classification levels (Fig 9(E) and 9(F)). In this latter MCN each taxon is classified at the species level if possible (rare with 16S), otherwise more commonly the genus level is used. Schemes regarding color, node size, and edge thickness are the same as Fig 8. Since the MCNs are now larger we do not label every node, only those that we reference in our analyses. We also extend Table 6 to include correlations from every taxonomic level, but as this is also very large we include it as S3 Table and extract only relevant portions to our discussion. We perform a similar task with ATria, and S4 Table.

**Fig 10:**
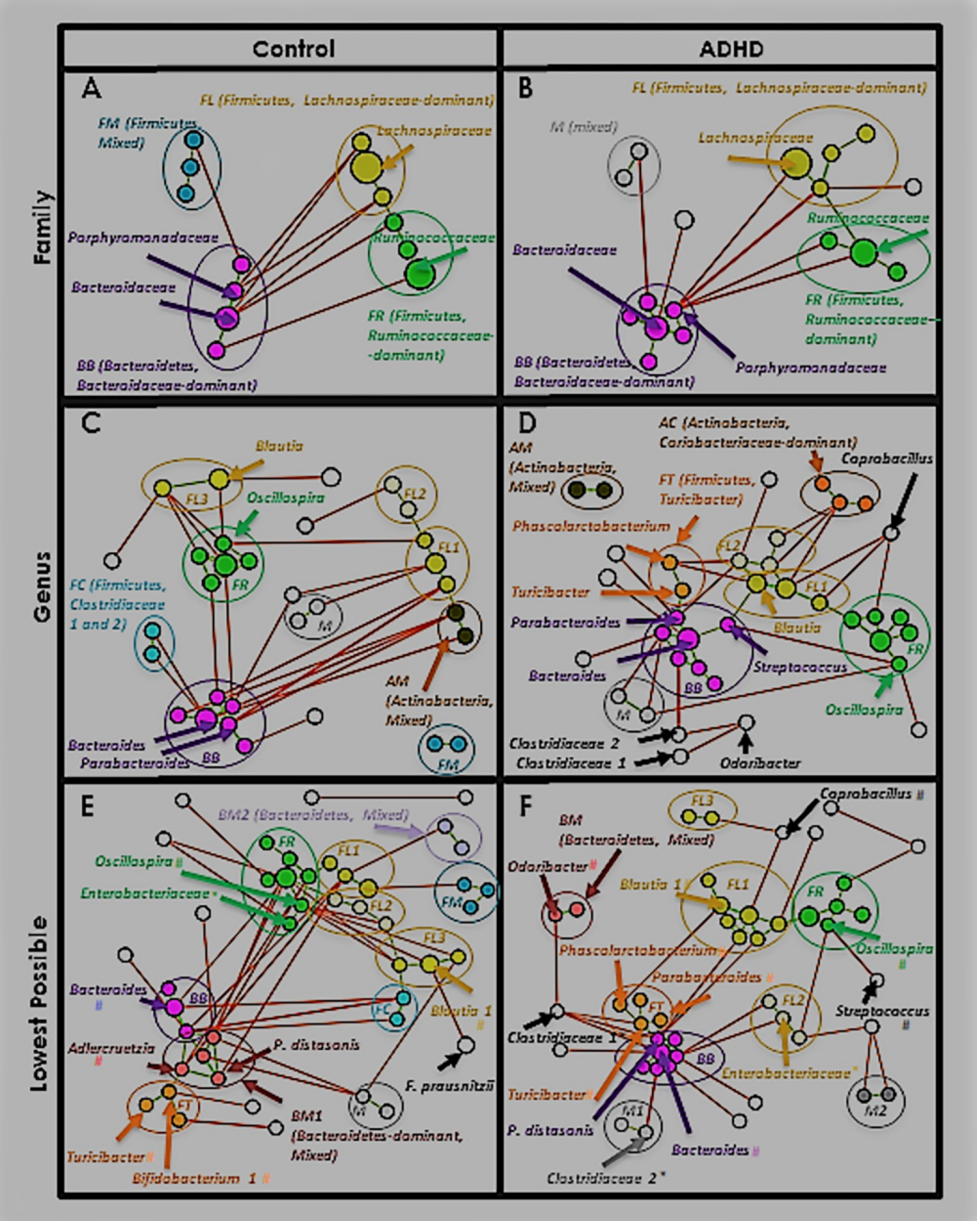
Clusters. Same MCNs as Fig 9, after clustering with the affinity propagation (AP) algorithm [85]. Family-level clusters are each given a unique color, and labeled with their dominant phylum and member family. New clusters that form at each lower taxonomic level are labeled, colored with shades corresponding to their dominant phylum/family when applicable—i.e. at the genus level *FL1*-*FL3* are different shades of gold (family-level *FL*). Taxa noted throughout our analyses are labeled (* = family-level taxon, # = genus-level taxon).

Fig 9 shows taxa separating into a group of primarily Bacteroidetes (dark purple, lower left), and another of primarily Firmicutes (yellow, upper right). Enough taxa are also now present to perform meaningful community analysis. Fig 10 shows the same MCNs as Fig 9, after running Affinity Propagation (AP, [112]) and coloring by cluster. At the family level (Fig 10(A) and 10(B)) four clusters form. One is dominated by Bacteroidetes, family *Bacteroidaceae* (*BB*, magenta). Two are dominated by Firmicutes, one family *Lachnospiraceae* (*FL*, gold), and the other family *Ruminococcaceae* (*FR*, green). In Control (Fig 10(A)) the fourth cluster consists of three mixed-family Firmicutes (*FM*, dark teal). In ADHD (Fig 10(B)) two of these are absent and the Proteobacteria family *Enterobacteriaceae* is present, leaving it no longer Firmicutes-dominant (*M*, grey).

Clusters *BB* and *FR* remain at the genus level (Fig 10(C) and 10(D)). Several Firmicutes, family *Lachnospiraceae*-dominant clusters emerge, referred to as *FL1*, *FL2*, etc. (gold shades). A mixed-family Actinobacteria cluster of genera *Bifidobacterium* and *Collinsella* forms in both MCNs (*AM*, brown), and an Actinobacteria, family *Coriobacteriaceae*-dominated cluster forms in ADHD (Fig 10(D), *AC*, burnt sienna). A small group of two *Clostridiaceae* family composes cluster *FC* in Control (Fig 10(C), aqua). In ADHD (Fig 10(D)), a cluster (orange) emerges as the only Firmicutes-dominant cluster with positive correlations to cluster *BB*. This eventually becomes present in both lowest-level MCNs (Fig 10(E) and 10(F)) with core member Control-elevated genus *Turicibacter*, so we call this cluster *FT*.

At the lowest level we kept cluster names as consistent as possible with genus-level membership (for example, a cluster mostly comprised of *FL2* genus-level taxa would also be named *FL2* at the lowest level). Both MCNs (Fig 10(E) and 10(F)) now include a mixed-family, Bacteroidetes-dominant cluster *BM1* (pink), and Control includes a second (*BM2*, orchid). S5–S7 Tables list all clusters, members, and centroids at all levels. As with earlier tables, we will extract portions relevant to our discussion.

**Fig 11:**
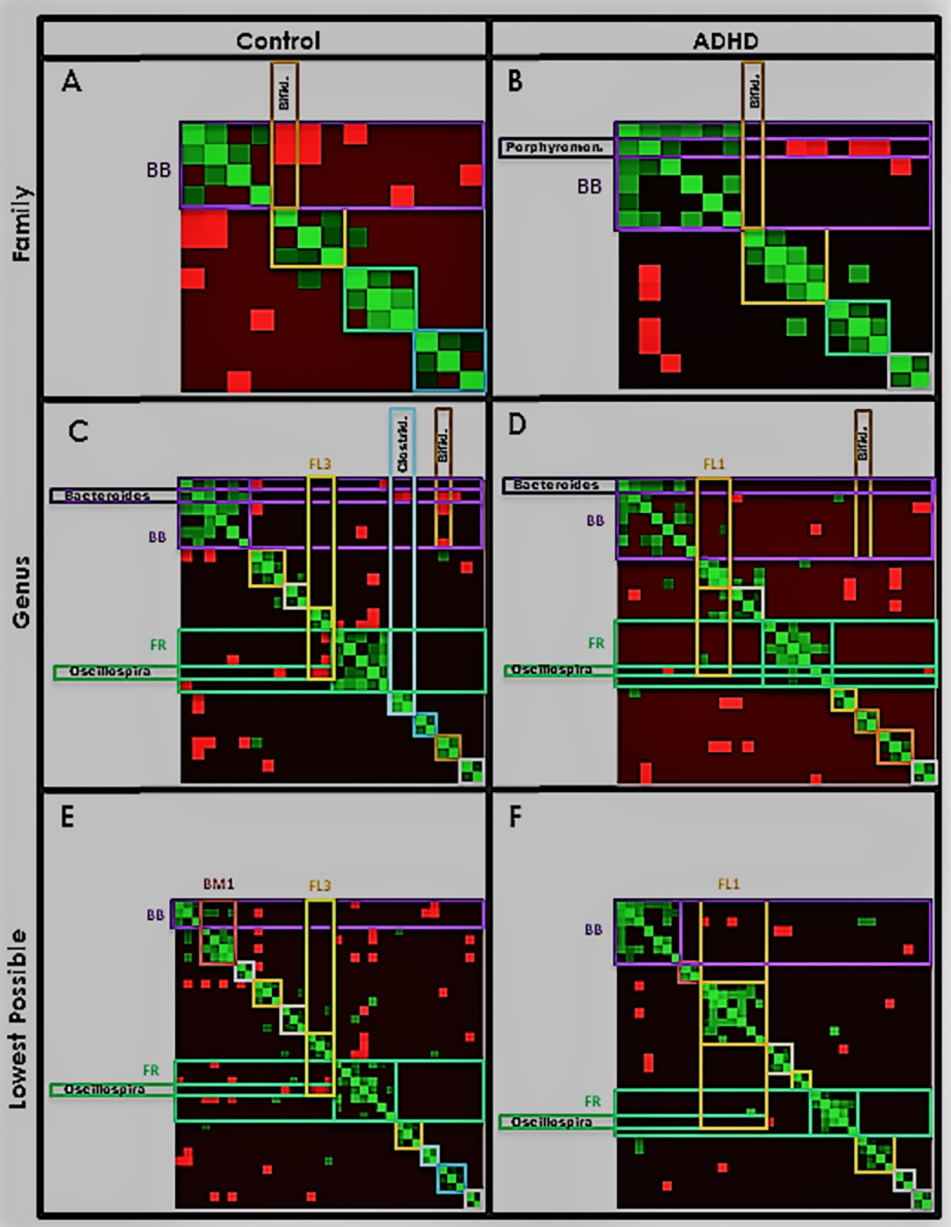
Heatmaps. Heatmap representation of taxa correlations (green = positive, red = negative), with taxa organized on each axis by cluster (symmetric matrix). The area corresponding to the intersection of each cluster with itself is outlined with a box using the corresponding cluster color in Fig 10. Taxa and clusters noted throughout our analyses are labeled on the axes.

Finally, to measure cluster size, tightness, and interactions, we produce a heatmap of taxa correlations (Fig 11) with taxa ordered on the *x*- and *y*-axes by Fig 10 cluster. Green/red intensity at each point (*x*, *y*) denotes the degree of positive/negative correlation between taxa *x* and *y* (symmetric, by definition). Clusters appear as rough squares of positive (green) correlations on the diagonal. We outline each box with the same color as its corresponding Fig 10 cluster.

We first continue to pursue observations (A)-(C) from the upper taxonomic levels. Afterwards, we discuss any new and interesting trends.

*(A) A core Proteobacteria-Bacteroidetes positive correlation (est*. *cooperation) forms*. Recall the orders involved in this correlation were Burkholderiales (Proteobacteria) and Bacteroidales (Bacteroidetes). **This corresponds to cluster *BB*, with genus *Sutterella* and multiple *Bacteroidales* taxa. In ADHD this cluster is larger and includes more *Bacteroidales* plus some Firmicutes, and nearly all members are positively correlated with its centroid *Bacteroides*. Additionally it has fewer negative correlations (est. competition) with other clusters.**

Cluster *BB* is the only cluster with order Burkholderiales and Bacteroidales descendants. Table 8 shows all correlations involving Burkholderiales and Bacteroidales lineages, organized and shaded using the same scheme as Table 6. One core positive correlation survives all six taxonomic levels in Control and ADHD (12 MCNs total, the only correlation in our entire dataset with this property). This occurs between genera *Sutterella* and *Bacteroides*. Several others involving *Sutterella* and its family *Alcaligenaceae* with cluster *BB* members are present only in ADHD–support for a larger cluster *BB* in ADHD. *Alcaligenaceae/Sutterella* are immediately visible in Fig 9, as the only Proteobacteria (royal blue) among a slew of Bacteroidetes (dark purple).

**Table 8:** Burkholderiales-Bacteroidales correlations.

Phylum		Class		Order		Family		Genus		Species	
Proteobacteria-Bacteroidetes	+	Betaproteobacteria-Bacteroidia	+	Burkholderiales-Bacteroidales	+	Alcaligenaceae-Bacteroidaceae	+	Sutterella-Bacteroides	+	Sutterella-Bacteroides	+
										Sutterella- B.uniformis	+
						Alcaligenaceae-Porphyromonadaceae	+	*Sutterella-Parabacteroides*		Sutterella-P. distasonis	+
						Alcaligenaceae-Odoribacteriaceae	+	Sutterella-Butyricimonas	+	Sutterella-Butyricimonas	+
						*Alcaligenaceae-Rikenellaceae*		Sutterella-Rikenellaceae	+		

Correlations between order Burkholderiales-Bacteroidales lineages, shaded using the same scheme as Table 6 (grey present in both MCNs, purple only ADHD).

Fig 10 also illustrates the increase in ADHD cluster *BB* size, as do the heatmaps (Fig 11, magenta square). Table 9 quantifies differences in node and edge count.

**Table 9:** Cluster BB size.

Taxonomic Level	Family		Genus		Lowest	
**MCN**	**Control**	**ADHD**	**Control**	**ADHD**	**Control**	**ADHD**
Cluster BB size: Taxa (+ Edges)	4 (3)	6 (7)	6 (7)	7 (9)	3 (2)	7 (11)

Control and ADHD cluster *BB* size. Notation: *Taxa (edges)*.

Table 9 shows cluster *BB* size to mysteriously drop in Control from the genus to the lowest level, from six taxa down to three. A closer look at Fig 10(C) and 10(E) shows several genus-level *BB* members may be joining a mixed-family, Bacteroidetes-dominant cluster (*BM1*, pink) at the lowest level. Table 10, which shows *BB* and Control *BM1* members, confirms this. Core *BB* members are shown in bold, while italicized members are unique to Control or ADHD. Taxa of genus-level Control cluster *BB* members *Odoribacter*, *Adlercruetzia*, *Parabacteroides* (*P*. *distasonis*) and *Bacteroides* (*B*. *ovatus*) compose Control cluster *BM1* at the lowest level.

**Table 10:** *Bacteroidaceae*/*Bacteroides* dominant clusters.

			Control		ADHD	
Level	Community	Cluster Type	Taxon	Phylum	Taxon	Phylum
**Family**	**Bacteroidetes-dominant (B)**	**Bacteroidaceae-dominant (BB)**	**Bacteroidaceae**		Bacteroidaceae*	
			Porphyromonadaceae*		**Porphyromonadaceae**	
			**Alcaligenaceae**		**Alcaligenaceae**	
			**Rikenellaceae**		**Rikenellaceae**	
					*Odoribacteraceae*	
					*Streptococcaceae*	
**Level**	**Community**	**Cluster Type**	**Taxon**	**Phylum**	**Taxon**	**Phylum**
**Genus**	**Bacteroidetes-dominant (B)**	**Bacteroidaceae-dominant (BB)**	**Bacteroides**		Bacteroides*	
			Parabacteroides*		**Parabacteroides**	
			**Sutterella**		**Sutterella**	
			**Rikenellaceae**		**Rikenellaceae**	
			*Odoribacter*		*Butyricimonas*	
			*Adlercruetzia*		*Streptococcus*	
					*Clostridium*	
**Level**	**Community**	**Cluster Type**	**Taxon**	**Phylum**	**Taxon**	**Phylum**
**Species**	**Bacteroidetes-dominant (B)**	**Bacteroidaceae-dominant (BB)**	Bacteroides*		Bacteroides*	
			**Bacteroides uniformis**		**Bacteroides uniformis**	
			**Sutterella**		**Sutterella**	
					*Parabacteroides distasonis*	
					*Rikenellaceae*	
					*Butyricimonas*	
					*Bifidobacterium longum*	
		**Bacteroidetes, Mixed (BM1)**	Odoribacter			
			Bacteroides ovatus			
			Parabacteroides distasonis*			
			Adlercruetzia			

*Bacteroides*, *Bacteroidaceae* dominant clusters (*BB*) and Bacteroidetes, Mixed family (*BM1*) cluster in Control. Core taxa are bold, taxa exclusive to one MCN (Control or ADHD) are italicized, and centroids are marked with an asterisk (*). Phylum: purple = Bacteroidetes, yellow = Firmicutes, brown-Actinobacteria, blue = Proteobacteria.

Table 11 supports weakened connections between *BB* and *BM1* taxa in Control, showing higher intra-correlation values (0.61 and 0.62) relative to inter-correlation (0.44).

**Table 11:** *Bacteroidaceae*-dominant community intra- and inter-correlations.

Control BB (Intra)			Control BM1 (Intra)			Control BB-BM1 (Inter)			ADHD BB (Intra)		
**Taxa**	**Edges**	**Mean Correlation**	**Taxa**	**Edges**	**Mean Correlation**	**Taxa**	**Edges**	**Mean Correlation**	**Taxa**	**Edges**	**Mean Correlation**
3	2	0.61 ± 0.15	4	4	0.62 ± 0.1	4	3	0.44 ± 0.02	7	11	0.56 ± 0.12

Intra-correlations between cluster *BB* members, and *BB*-*BM1* inter-correlations in Control.

Table 10 also shows cluster *BB* members that differ between the MCNs. Cluster *BB* gains a different Actinobacteria–species *B*. *longum* (ADHD) and genus *Adlercruetzia* (Control, eventually joining *BM1*). The presence of Firmicutes (yellow) is exclusive to ADHD, including family *Streptococcaceae* and member genus *Streptococcus*, plus genus *Clostridium*. Family *Odoribacteriaceae* (reported by LEfSe as ADHD-elevated) joins cluster *BB* only in ADHD, and the sole *Clostridium* connection to cluster *BB* is with genus *Butyricimonas* (Fig 9(D)), a taxon reported as ADHD differentially abundant by two methods (LEfSe and ALDEx2).

Table 10 also indicates *BB*/*BM1* centroids, which we see across the board for ADHD are genus *Bacteroides* and its family *Bacteroidaceae*. In Control this belongs to *Porphyromonadaceae* (family) and descendant *Parabacteroides* (genus), until the *BB*-*BM1* “split” where genus *Bacteroides* becomes centroid of *BB* and species *P*. *distasonis* of *BM1*. Table 12 shows connectivity of each of these taxa within their corresponding cluster. Percentagewise, in ADHD *Bacteroidaceae*/*Bacteroides* is a much stronger centroid; in fact over all levels only one cluster *BB* taxon was not positively correlated (*Clostridium*, genus level). Particularly given the ADHD cluster *BB* size increase, this could imply a significant role of *Bacteroidaceae*/*Bacteroides* in stabilizing a large ADHD Bacteroidetes-dominant community (would require additional experiments to verify).

**Table 12:** Cluster *BB* centroid connectivity.

Level	Family		Genus		Lowest	
MCN	Control	ADHD	Control	ADHD	Control	ADHD
*Bacteroidaceae*/*Bacteroides*	2/3 (66%)	5/5 (100%)	3/5 (60%)	5/6 (84%)	2/2 (100%)	6/6 (100%)
*Porphyromonadaceae/Parabacteroides/P*. distasonis*	2/3 (66%)	2/5 (40%)	4/5 (80%)	3/6 (50%)	3/3* (100%)	3/6 (50%)

Connectivity between the centroid of cluster *BB* (* = *BM1*) and the rest of its cluster members.

Interestingly ATria (Table 13) shows *Bacteroidaceae/Bacteroides* and lineages to nearly always have higher importance in Control, supporting a more “global” importance to overall gut ecology as opposed to a more local importance (cluster *BB*) in ADHD. MCNs agree, as in ADHD *Bacteroidaceae/Bacteroides* have few connections outside cluster *BB* (Fig 10(B), 10(D) and 10(F)). In Control (Fig 10(A), 10(C) and 10(E)) *Bacteroidaceae/Bacteroides* have many external connections, mostly negative (est. competition).

**Table 13:** Bacteroidetes rankings.

Phylum	Class	Order	Family	Genus	Lowest Possible
Bacteroidetes (NR/#T2)	**Bacteroidia (NR/#1)**	**Bacteroidales (#1/#1)**	Bacteroidaceae (#T9/#T12)	Bacteroides (#10/#T23)	B. uniformis (#12/#7)
					B. ovatus (#2/#T20)
					Bacteroides (#T23/#T28)
			Odoribacteriaceae (NR/#2)	Odoribacter (#9/#T21)	Odoribacter (#3/#T20)
				Butyricimonas (NR/#4)	Butyricimonas (NR/#17)
			**Porphyromonadaceae (#T7/#1)**	Parabacteroides (#T12/NR)	Parabacteroides (#T19/#T24)
					P. distasonis (NR/#T24)
			Prevotellaceae (NR/#5)	Prevotella (NR/#18)	
			Rikenellaceae (#T7/#3)	Rikenellaceae (#T12/#8)	Rikenellaceae (#T19/#8)

ATria rankings of Bacteroidetes taxa. Dark orange = only ranked in Control, dark purple = only ranked in ADHD, light orange = higher ranked in Control, light purple = higher ranked in ADHD, grey = evenly ranked. **Bold** taxa are ranked #1.

Control MCNs (Fig 10(A), 10(C) and 10(E) and heatmaps) (Fig 11(A), 11(C) and 11(E), magenta rectangle) show negative correlations (red) to be fairly evenly distributed among cluster *BB* taxa. By contrast in ADHD (Figs 10–11(B)), nearly all cluster *BB* negative correlations are localized to family *Porphyromonadaceae* (ranked #1 by ATria). Fig 9(B) shows *Porphyromonadaceae* to be the sole cluster *BB* member negatively correlated with the Firmicutes-dominant portion (Fig 9(B), upper right, collectively more than 70% of the population).

Table 14 shows that for all MCNs, in Control more than two-thirds of cluster *BB* had negative correlations with members of other clusters, compared to less than half in ADHD. Negative edge count was also almost always higher for Control, despite a smaller cluster *BB*. Collectively these results show that in Control cluster *BB* is smaller, and more connected to other clusters, primarily through negative correlations (est. competition). In ADHD cluster *BB* is larger, and more isolated.

**Table 14:** Negative correlations between cluster *BB* and other clusters.

Level	Family		Genus		Lowest	
MCN	Control	ADHD	Control	ADHD	Control	ADHD
Cluster BB (-) Edges with Other Clusters (Participation Rate)	7 (100%)	5 (33%)	10 (88%)	4 (43%)	5 (67%)	5 (43%)

Amount of negative correlations between members of *Bacteroidaceae*-dominant cluster *BB* and other clusters. Notation: *Number (participation rate)*.

Table 15 provides a few final interesting observations for various Bacteroidetes taxa.

**Table 15:** Additional Bacteroidetes observations.

Taxa	Observation
Family *Odoribacteriaceae*	ADHD-elevated, ADHD cluster *BB* member, only ranked in ADHD.
Family *Prevotellaceae* and member genus *Prevotella*	ADHD negative correlation with *Bacteroidaceae*/*Bacteroides* is the only negative correlation between two Bacteroidetes taxa in any MCN. Only ranked in ADHD,
Family *Rikenellaceae*	Ranked in every MCN at every level, and always higher for ADHD.

Additional observations we make for some other Bacteroidetes taxa.

*(B) In Control*, *taxa in (A) have more negative edges with Actinobacteria (est*. *competition)*, *especially Bifidobacteriales*. We now know taxa from (A) to correspond to cluster *BB*, which in both MCNs contained one core Proteobacteria (family *Alcaligenaceae*/genus *Sutterella*) and otherwise primarily Bacteroidetes. We also observed cluster *BB* taxa to have far more negative correlations (est. competition) with other clusters in Control. We now see if this is also true with order Bifidobacteriales lineages, including genus *Bifidobacterium*. Our analysis in fact reveals that **negative correlations between *Bifidobacterium* or** **any** **parent/descendant with** **any** **Bacteroidetes or Proteobacteria are exclusive to Control and absent in ADHD.**

Table 16 shows all correlations involving *Bifidobacterium* and its lineages, grouped and colored as in previous tables. Not only are negative Bacteroidetes correlations exclusive to Control (orange), but these taxa include the most abundant Bacteroidetes family/genus *Bacteroidaceae*/*Bacteroides* (ADHD cluster *BB* centroid), as well as family/genus *Porphyromonadaceae*/*Parabacteroides* (Control cluster *BB* centroid). Another appears at the lowest level between soecues *B*. *adolescentis* and *B*. *ovatus*. With Proteobacteria, negative *Bifidobacterium* correlations are also observed with genus *Sutterella* (core cluster *BB* member) and family *Enterobacteriaceae*, also only in Control. Heatmaps confirm family/genus *Bifidobacteriaceae*/*Bifidobacterium* to be negatively correlated with cluster *BB* taxa only in Control (Fig 11(A)–11(D), intersection of brown and magenta rectangles). By contrast, the only ADHD correlation is positive and within cluster *BB* (species *B*. *longum* with species *B*. *uniformis*).

**Table 16:** *Bifidobacterium* correlations.

Phylum		Class		Order		Family		Genus		Lowest Possible	
Actinobacteria-Bacteroidetes	-	Actinobacteria- Bacteroidia	-	Bifidobacteriales-Bacteroidales	-	Bifidobacteriaceae–Bacteroidaceae	-	Bifidobactrium -Bacteroides	-	B. adolescentis–B. ovatus	-
										B. longum–B.uniformis	+
						Bifidobacteriaceae–Porphyromonadaceae	-	Bifidobacterium—Parabacteroides	-		
Actinobacteria Proteobacteria	-	Actinobacteria -Betaproteobacteria	-	*Bifidobacteriales-Burkholderiales*		*Bifidobacteriaceae-Alcaligenaceae*		Bifidobacterium– Sutterella	-		
		*Actinobacteria-Gammaproteobacteria*		*Bifidobacteriales- Enterobacteriales*		*Bifidobacteriaceae-Enterobacteriaceae*		*Bifidobacterium-Enterobacteriaceae*		Bifidobacterium 2 –Enterobacteriaceae	-
*Actinobacteria- Firmicutes*		*Actinobacteria- Bacilli*		*Bifidobacteriales-Turicibacteriales*		*Bifidobacteriaceae-Turicibacteriaceae*		*Bifidobacterium-Turicibacter*		Bifidobacterium 1 –Turicibacter	+
		*Actinobacteria-Clostridia*		Bifidobacteriales -Clostridiales	+	Bifidobacteriaceae- Lachnospiraceae	+	Bifidobacterium -Lachnospiraceae 2	+	Bifidobacterium 1 –Blautia 2	-
										Bifidobacterium 1 –Ruminococcus [L]	+
										B. longum–Blautia 1	+
						*Bifidobacteriales–Clostridiales*		*Bifidobacterium-Clostridiales*		B. adolescentis–Clostridiales 1	+
								*Bifidobacterium-Ruminococcaceae*		Bifidobacterium 2 –Ruminococcaceae	-
										B. longum–Oscillospira	-
						*Bifidobacteriaceae–Veillonellaceae*		*Bifidobacterium-Veillonella*		Bifidobacterium 2 –Dialister	-
*Actinobacteria-Actinobacteria*		*Actinobacteria-Actinobacteria*		*Bifidobacteriales–Bifidobacteriales*		*Bifidobacteriaceae–Bifidobacteriaceae*		*Bifidobacterium-Bifidobacterium*		Bifidobacterium 2 –B. adolescentis	-
		*Actinobacteria-Coriobacteria*		Bifidobacteriales-Coriobacteriales	+	Bifidobacteriaceae-Coriobacteriaceae	+	*Bifidobacterium-Adlercruetzia*		Bifidobacterium 1 -Adlercruetzia	+
								Bifidobacterium-Collinsella	+		
								*Bifidobacterium-Eggerthella*		B. longum–E.lenta	-

Correlations involving *Bifidobacterium* and its lineages. Orange = only found in Control, purple = only found in ADHD, grey = found in both. +(green) = positive correlation, -(red) = negative correlation.

Table 16 also shows *Bifidobacterium* to even have far more Firmicutes connections (positive and negative) in Control. Collectively 24 correlations were observed in Control, compared to 9 in ADHD, supporting an overall increase in *Bifidobacterium* participation in Control. ATria (Table 17) also almost uniformly ranks *Bifidobacterium* and its lineages higher in Control. Again, this is despite *Bifidobacterium* average abundances being relatively the same (slightly higher in ADHD in fact, 3.6% to 3.2%).

**Table 17:** *Bifidobacterium* rankings.

Phylum	Class	Order	Family	Genus	Lowest Possible
**Actinobacteria (#1/#1)**	Actinobacteria (#2/NR)	Bifidobacteriales (#T2/ #T5)	Bifidobacteriaceae (#5/NR)	Bifidobacterium (NR/#T19)	Bifidobacterium 1 (#10/NR)
					**Bifidobacterium 2 (#1/11)**
					B. longum (#15/NR)

ATria rankings of *Bifidobacterium* and lineages. Dark orange = only ranked in Control, dark purple = only ranked in ADHD, light orange = higher ranked in Control, light purple = higher ranked in ADHD, grey = evenly ranked. **Bold** taxa are ranked #1.

*(C) A Shift in Firmicutes-Proteobacteria dynamics*. Only two Proteobacteria families/genera were consistently present. One was genus *Sutterella* (family *Alcaligenaceae*), already noted as a core cluster *BB* member. The other is family ***Enterobacteriaceae*, which our analysis supports being mostly responsible for this shift**.

Table 18 shows all Proteobacteria-Firmicutes correlations. A couple of negative correlations can be seen involving family/genus *Alcaligenaceae*/*Sutterella*, with Firmicutes families *Ruminococcaceae* (Control) and *Clostridiaceae* (both). Far more significant are the differences involving family *Enterobacteriaceae*. One is its negative correlation with genus *Oscillospira* in ADHD (genus level), that becomes a positive correlation with *Oscillospira* in Control (lowest level). This is the only time, over all twelve MCNs, where a correlation sign changed between the same two taxa in Control vs. ADHD.

**Table 18:** Proteobacteria-Firmicutes correlations.

Phylum		Class		Order		Family		Genus		Lowest Possible	
Proteobacteria- Firmicutes	-	*Betaproteobacteria—Clostridia*		*Burkholderiales-Clostridiales*		Alcaligenaceae–Ruminococcaceae	-				
						Alcaligenaceae—Clostridiaceae-	-	Sutterella–Clostridiaceae 2	-	Sutterella–Clostridiaceae 2	-
		*Gammaproteobacteria—Bacilli*		Enterobacteriales—Turicibacteriales	-						
		Gammaproteobacteria–Clostridia	-	*Enterobacteriales-Clostridiales*		Enterobacteriaceae–Mogibacteriaceae	+				
						*Enterobacteriaceae-Lachnospiraceae*		*Enterobacteriaceae–Anaerostipes*		Enterobacteriaceae-Anaerostipes	+
						*Ruminococcaceae-Enterobacteriaceae*		Enterobacteriaceae–Oscillospira	-	Enterobacteriaceae–Oscillospira	+
		*Gammaproteobacteria—Erysipelotrichi*		*Enterobacteriales-Erysipelotrichiales*		*Enterobacteriaceae-Erysipelotrichiaceae*		Enterobacteriaceae–Erysipelotrichiaceae 2	+	Enterobacteriaceae–Erysipelotrichiaceae 2	+
		Deltaproteobacteria—Clostridia	-	Desulfovibrionales–Clostridiales	-						

Correlations between Proteobacteria and Firmicutes taxa. Orange = only found in Control, purple = only found in ADHD, grey = found in both. +(green) = positive correlation, -(red) = negative correlation.

Interesting shifts involving family *Enterobacteriaceae* and various Firmicutes occur even at the family level, however. A small mixed-family, Firmicutes-dominant cluster *FM* forms (Fig 10(A), upper left), consisting of families *Mogibacteriaceae*, *Christensenellaceae*, and *Erysipelotrichiaceae* (Table 19). In ADHD, *Enterobacteriaceae* instead joins family *Mogibacteriaceae* to form a small two-taxon mixed cluster *M* (Fig 10(B), upper left, and Table 19).

**Table 19:** Mixed-family clusters.

		Control			ADHD		
Community	Cluster Type	Cluster	Taxon	Phy	Cluster	Taxon	Phy
**Firmicutes-dominant (F)**	**Mixed (FM)**	***FM***	*Mogibacteriaceae*				
		***FM***	Christensellaceae*				
		***FM***	*Erysipelotrichiaceae*				
**Mixed (M)**	**N/A**				***M***	*Mogibacteriaceae*	
					***M***	*Enterobacteriaceae*	

Control and ADHD clusters consisting of taxa from multiple families. Phylum: purple = Bacteroidetes, yellow = Firmicutes, brown-Actinobacteria, blue = Proteobacteria.

Dynamics of *FM* and *M* taxa change between the MCNs. Fig 10(A) and 10(B) shows a distinguishing core *FM*/*M* feature is the negative correlation with family *Rikenellaceae* of cluster *BB*, but the taxon involved changes from family *Erysipelotrichiaceae* in Control to family *Mogibacteriaceae* in ADHD. Table 20 (ATria) shows the two taxa from Control cluster *FM* “replaced” by family *Enterobacteriaceae* in ADHD cluster *M*, families *Christensenellaceae* and *Erysipelotrichiaceae*, are only ranked in Control, and family *Mogibacteriaceae* only ranked in ADHD. This applied across all descendants, with the one notable exception being genus *Coprobacillus* (family *Erysipelotrichiaceae*), ranked #1 for ADHD at the genus and lowest levels (the only taxon to be ranked #1 in two MCNs). Recall *Coprobacillus* was also reported by DESeq2 as elevated in ADHD compared to Control. Other members of the *Erysipelotrichaceae* family (including the family itself) were only ranked by ATria in the case of Control. We label it in Fig 10(D) and 10(F), noting its negative correlations with multiple Firmicutes-dominant clusters.

**Table 20:** Mixed-family cluster member rankings.

Phylum	Class	Order	Family	Genus	Lowest Possible
Firmicutes (#2/#T2)	Clostridia (#T3/#T3)	Clostridiales (#T2/#3)	Christensenellaceae (#2/NR)	*Christensenellaceae (NR/NR)*	Christensenellaceae (#18/NR)
			Mogibacteriaceae (NR/#T6)	Mogibacteriaceae (NR/#6)	
	*Erysipelotrichia (NR/NR)*	Erysipelotrichales (NR/#T3)	Erysipelotrichaceae (#3/NR)	**Coprobacillus (NR/#1)**	**Coprobacillus (#T31/#1)**
				Erysipelotrichaceae 1 (#6/NR)	Erysipelotrichaceae 1 (#16/NR)
				Erysipelotrichaceae 2 (#11/NR)	
				Eubacterium (#T14/NR)	E. dolicum (#T29/NR)
*Proteobacteria (NR/NR)*	Gammaproteobacteria (#T3/NR)	Enterobacteriales (#T3/NR)	Enterobacteriaceae (NR/#T6)	Enterobacteriaceae (NR/#7)	Enterobacteriaceae (NR/#T26)

Members of mixed family clusters from Table 19, their lineages, and ATria rankings. Dark orange = only ranked in Control, dark purple = only ranked in ADHD, light orange = higher ranked in Control, light purple = higher ranked in ADHD, grey = evenly ranked. **Bold** taxa are ranked #1.

Family *Enterobacteriaceae* was also only ranked in ADHD, across all three lower levels. Its *Oscillospira* positive correlation (Table 18) is the only Control correlation involving *Enterobacteriaceae*, and *Enterobacteriaceae* actually joins genus *Oscillospira*’s cluster (*FR*, Fig 10(E)*)* in Control. The sign change takes place at the genus level in ADHD (Fig 9(D)), where *Oscillospira* and *Enterobacteriaceae* are negatively correlated. Although this correlation did not persist to the lowest level (Fig 9(F)), *Enterobacteriaceae* is still positively correlated with genus *Anaerostipes*, a taxon negatively correlated with *Oscillospira* across the board. We therefore observe *Enterobacteriaceae* dynamics to shift from a state that favors *Oscillospira* cooperation in Control, to *Oscillospira* competition in ADHD. The role of *Enterobaceriaceae* in gut ecology has historically been controversial [145], with both beneficial [146] and pathogenic [147] properties emerging. Gut dysbiosis has actually been shown to trigger horizontal gene transfer between the two types [148].

#### New observations

We make the following new observations at the lower levels.

*(D) LEfSe reported taxa*: *Genera Turicibacter and Odoribacter*. Earlier we noted ADHD-elevated family *Odoribacteriaceae* as an ADHD cluster *BB* member (Table 15). We now observe two remaining LEfSe-reported genera: *Turicibacter* (Control) and *Odoribacter* (ADHD).

Cluster *FT* (Fig 10, orange) was the only Firmicutes-dominant cluster with members positively correlated with any Bacteroidetes-dominant cluster (*BB* in ADHD, *BM1* in Control). We named this cluster *FT* because of core member genus *Turicibacter*. *Turicibacter* (Firmicutes, Control-elevated), which joins genus *Phascolarctobacterium* (Firmicutes, reduced in Inattention, [63]) to form *FT* at the genus level in ADHD (Fig 10(D)), where it is not present in Control. At the lowest level, *FT* is slightly larger (by one taxon) in ADHD. Supplementing the earlier trend of less cluster *BB* negative correlations (est. competition) in ADHD, this also supports the presence of a larger cluster with positive correlations (est. cooperation) as well, with *Turicibacter* as its centroid (Table 21).

**Table 21:** *FT* members.

			Control		ADHD	
Level	Community	Cluster Type	Taxon	Phy	Taxon	Phy
**Family**	**Firmicutes-dominant (F)**	**Turicibacter-core (FT)**	Not present.		*Turicibacter*	
					*Phascolarctobacterium*	
**Level**	**Community**	**Sub-Community**	**Taxon**	**Phy**	**Taxon**	**Phy**
**Genus**	**Firmicutes-dominant (F)**	**Turicibacter-core (FT)**	**Turicibacter**		Turicibacter*	
			*Ruminococcus [L]*		*Phascolarctobacterium*	
			Bifidobacterium 2*		*Clostridiales 2*	
					*Parabacteroides*	

Cluster FT (Firmicutes-dominant, *Turicibacter*-core) members. Phylum: purple = Bacteroidetes, yellow = Firmicutes, brown-Actinobacteria, blue = Proteobacteria.

In ADHD *Turicibacter* provides the sole genus-level (Fig 10(D)) *FT*-*BB* positive correlation, with genus *Parabacteroides* (Bacteroidetes, previously reported elevated in Hyperactivity, [59]). At the lowest level (Fig 10(F)) *Parabacteroides* joins *FT*, and along with *Turicibacter* forms *FT*-*BB* positive correlations, with member species *P*. *distasonis*. Interestingly in Control (Fig 10(E)), the *FT*-*BB* positive correlation does not involve Firmicutes or Bacteroidetes taxa at all, but rather two Actinobacteria–*Bifidobacterium 1 (FT* centroid*)*, and *Adlercruetzia* (*BB*). This continues our observed increases in Actinobacteria and particularly *Bifidobacterium* involvement in Control gut ecology.

Cluster *FC* forms in Control (Fig 10(C) and 10(E), aqua) and contains two family *Clostridiaceae* taxa. In both MCNs these taxa negatively correlate with multiple cluster *BB* members, and in ADHD (Fig 10(F)) family *Clostridiaceae 1* has negative correlations with *BB* centroid genus *Bacteroides* plus taxa involved in *FT*-*BB* cooperation: species *P*. *distasonis*, and *FT* centroid genus *Turicibacter*. In both MCNs, they participate in correlations that favor cluster *BB* competition (especially the more abundant *Clostridiaceae 1*).

Exclusive to ADHD is a negative correlation (est. competition) between these family *Clostridiaceae* taxa and ADHD-elevated genus *Odoribacter*–both at the genus level (Fig 10(D)), and family *Clostridiaceae 1* at the lowest level (Fig 10(F)). Genus *Odoribacter* was reported by LEfSe as elevated in ADHD, and this negative correlation implies that an increase in *Odoribacter* abundance will decrease family *Clostridiaceae 1*. ALDEx2 reported *Clostridiaceae 1* as differentially abundant in Control, and upon further inspection *Clostridiaceae 1* average relative abundance is indeed reduced by a factor of two in ADHD vs. Control. Cooperation between families *Clostridiaceae 1* and *2* in Control (forming *FC*) is also absent in ADHD.

*(E) Changes in the role of genus Adlercruetzia (Actinobacteria)*. In contrast to genus *Bifidobacterium* (*Bifidobacteriaceae*), *Adlercruetzia* is a member of the other consistently present Actinobacteria family, *Coriobacteriaceae*. While the distinguishing feature of family/genus *Bifidobacteriaceae/Bifidobacterium* was increased Control participation, the distinguishing feature of family *Coriobacteriaceae* appears to be changes in cluster membership. In fact over all *Coriobacteriaceae* descendants, only once (*Collinsella*, genus level, cluster AM, Fig 10(C) and 10(D)) were any in the same Control and ADHD cluster. Table 22 also shows ATria results to be more mixed for family *Coriobacteriaceae*, compared to family *Bifidobacteriaceae* (Table 19).

**Table 22:** *Coriobacteriaceae* rankings.

Phylum	Class	Order	Family	Genus	Lowest Possible
Actinobacteria (#1/#1)	**Coriobacteria (#1/NR)**	Coriobacteriales (NR/#T5)	Coriobacteriaceae (NR/#4)	Adlercruetzia (NR/#17)	Adlercruetzia (#14/NR)
				Collinsella (NR/#T19)	C. aerofaciens (NR/#15)
				Coriobacteriaceae (#18/NR)	
				Eggerthella (#4/NR)	E. lenta (#T25/NR)

ATria rankings of *Coriobacteriaceae* and its lineages. Dark orange = only ranked in Control, dark purple = only ranked in ADHD, light orange = higher ranked in Control, light purple = higher ranked in ADHD, grey = evenly ranked. **Bold** taxa are ranked #1.

We earlier noted genus *Adlercruetzia* as the Actinobacteria member of cluster *BB*/*BM1* in Control, and (along with genus *Bifidobacterium 1*) connecting clusters *FT* and *BB*. Table 23 shows that outside of *Bifidobacterium 1*, its positive correlations in Control were entirely with Bacteroidetes taxa (all *BB/BM1* members). By contrast in ADHD, *Adlercruetzia* relationships mostly occur with Firmicutes, including a cluster membership with genus/species *Eubacterium/E*. *dolicum*. Several negative correlations are seen between genus *Adlercruetzia* and different Firmicutes, with no overlap between Control and ADHD. This suggests genus *Adlercruetzia* may play a significantly different role in Control and ADHD gut ecologies.

**Table 23:** *Adlercruetzia* correlations.

Phylum		Class		Order		Family		Genus		Lowest Possible	
Actinobacteria-Bacteroidetes	-	Coriobacteria-Bacteroidia	-	Coriobacteriales -Bacteroidales	-	Coriobacteriaceae–Bacteroidaceae	-	Adlercreutzia- Bacteroides	+	Adlercreutzia- B.uniformis	+
								Adlercreutiza-Parabacteroides	+	Adlercreutzia- P.distasonis	+
								Adlercreutzia- Odoribacter	+		
*Actinobacteria-Firmicutes*		*Actinobacteria-Clostridia*		*Coriobacteriales- Costridiales*		*Coriobacteriaceae- Erysipelotrichaceae-*		Adlercreutzia- Eubacterium	*+*	Adlercreutzia- E. dolicum	+
						*Coriobacteriaceae- Lachnospiraceae*		Adlercreutzia -Blautia	-		
								Adlercreutzia–Lachnospira	-	Adlercreutzia—Lachnospira	-
								Adlercreutzia—Lachnospiraceae 1	-		
								Adlercreutzia—Lachnospiraceae 2		Adlercreutzia—Lachnospiraceae 2	-
						*Coriobacteriaceae- Ruminococcaceae*		Adlercreutzia–Ruminococcus	-	Adlercreutzia–Ruminococcus	-
*Actinobacteria-Actinobacteria*		*Actinobacteria-Coriobacteria*		Bifidobacteriales-Coriobacteriales	+	Bifidobacteriaceae-Coriobacteriaceae	+	*Bifidobacterium-Adlercruetzia*		Bifidobacterium 1 -Adlercruetzia	+
		*Coriobacteria—Coriobacteria*		*Coriobacteriales—Coriobacteriales*		*Coriobacteriaceae-Coriobacteriaceae*		Adlercruetzia-Coriobacteriaceae	+		

Correlations involving genus *Adlercreutzia* and its lineages. Orange = only found in Control, purple = only found in ADHD, grey = found in both. +(green) = positive correlation, -(red) = negative correlation.

*(F) Bacteroidetes-Firmicutes positive correlations (est*. *cooperation) are entirely exclusive to ADHD*, *and absent in Control*. Table 24 shows all Bacteroidetes-Firmicutes positive correlations. They are entirely limited to ADHD, and with one exception (genus *Clostridium*) involve class Bacilli descendants.

**Table 24:** Bacteroidetes-Firmicutes positive correlations.

Phylum		Class	Order		Family		Genus		Lowest Possible	
Bacteroidetes-Firmicutes	-	*Bacteroidia -Bacilli*	*Bacteroidales Lactobacillales*		Bacteroidaceae Streptococcaceae	+	Bacteroides -Streptococcus	+		
					*Porphyromonadaceae Turicibacteriaceae-*		Parabacteroides -Turicibacter	+	Parabacteroides-Turicibacter	+
							Parabacteroides -Turicibacter	+	P.distasonis -Turicibacter	+
			Bacteroidales–Clostridiales	-	*Odoribacteriaceae–Clostridiaceae*		Butyricimonas -Clostridium	+		

Bacteroidetes-Firmicutes positive correlations, over all MCNs. Orange = only found in Control, purple = only found in ADHD, grey = found in both. +(green) = positive correlation, -(red) = negative correlation.

We have already seen most of these, including genera *Clostridium* and *Butyricimonas*, and the ADHD *FT*-*BB* connections involving genera *Turicibacter* and *Parabacteroides*, and species *P*.*distasonis*. We now analyze the remaining top row, between genera *Bacteroides* (family *Bacteroidaceae*) and *Streptococcus* (family *Streptococcaceae*).

Firmicutes taxa were only ever present in cluster *BB* in ADHD, and we earlier noted family *Streptococcaceae* and its genus *Streptococcus* as two of those taxa. Their cluster *BB* positive correlation was with centroid family/genus *Bacteroidaceae*/*Bacteroides*. Additionally cluster *BB* had almost no negative correlations (est. competition) with *FL*/*FR* (collectively 70% of the population) in ADHD, compared to a significant amount in Control.

What makes genus *Streptococcus* interesting for ADHD is that across all MCNs, it forms the only positive correlation between cluster *BB* and *FL/FR* (Fig 10(D)). In other words, in addition to estimating significantly less *BB*-(*FL*/*FR*) competition in ADHD, our MCNs also estimate cooperation only in ADHD, between genera *Streptococcus* (*BB*) and *Blautia* (*FL1*).

Fig 10(D) and 10(F) also show *Streptococcus* to be negatively correlated with genus *Oscillospira* in ADHD, a taxon we noted earlier its correlation sign change with family *Enterobacteriaceae*. ATria (Table 25) also only ranks family/genus *Streptococcaceae*/*Streptococcus* as important in ADHD.

**Table 25:** *Streptococcaceae*/*Streptococcus* rankings.

Phylum	Class	Order	Family	Genus	Lowest Possible
Firmicutes (#2/#T2)	*Bacilli (NR/NR)*	*Lactobacillales (NR/NR)*	Streptococcaceae (NR/#T12)	Streptococcus (NR/#T23)	

ATria rankings of family/genus *Streptococcaceae*/*Streptococcus*. Dark orange = only ranked in Control, dark purple = only ranked in ADHD, light orange = higher ranked in Control, light purple = higher ranked in ADHD, grey = evenly ranked. **Bold** taxa are ranked #1.

*(G) A shift in genera Blautia-Oscillospira dynamics*, *and their respective clusters*. Thus far genus *Oscillospira* has been noted for two ADHD-exclusive negative correlations, with taxa only ranked in ADHD: family *Enterobacteriaceae* and genus *Streptococcus*. *Enterobacteriaceae*-*Oscillospira* was the only correlation to ever change sign from Control (positive) to ADHD (negative). *Streptococcus* was noted for its correlation with genus *Blautia*, the sole positive correlation between the largest Bacteroidetes-dominant cluster (*BB*) and Firmicutes-dominant clusters (*FL/FR*) in any MCN.

Previous studies have indicated butyrate-producing *Oscillospira* as a healthy gut taxon [149], specifically associated with leanness [150]. *Blautia* is actually a taxon that has been associated with obesity [151]. And interestingly in the Control MCN (Fig 10(C) and 10(E)) *Blautia* and *Oscillospira* are negatively correlated, but not in ADHD (Fig 10(D) and 10(F)).

Since obesity has been associated with ADHD [152], the shift in *Enterobacteriaceae* (*Oscillospira* cooperation in Control, competition in ADHD) and *Streptococcus* (*Blautia* cooperation and *Oscillospira* competition in ADHD) correlations become interesting, favoring *Blautia* cooperation and *Oscillospira* competition. Indeed correlation can never imply causation and further experimental verification is required. But ATria results (Table 26) also support this, ranking *Blautia* higher in ADHD and *Oscillospira* in Control.

**Table 26:** *Blautia* and *Oscillospira* rankings.

Phylum	Class	Order	Family	Genus	Lowest Possible
Firmicutes (#2/#T2)	Clostridia (#T3/#T3)	Clostridiales (#T2/#3)	Lachnospiraceae (NR/NR)	Blautia (#T16/#13)	Blautia 1* (NR/#19)
			Ruminococcaceae (#4/#T8)	Oscillospira (#3/#10)	Oscillospira (#5/#9)

*Blautia* and *Oscillospira* ATria rankings (plus lineages). Dark orange = only ranked in Control, dark purple = only ranked in ADHD, light orange = higher ranked in Control, light purple = higher ranked in ADHD, grey = evenly ranked. **Bold** taxa are ranked #1. * = The lowest level had two *Blautia* taxa; we assumed the more abundant (*Blautia* 1, overall 9.3% relative abundance vs 0.6%, composing 93% of the *Blautia* population).

In fact our heatmap (Fig 11(C)–11(F)) shows by intersecting *Oscillospira*’s row (small green rectangle) with the columns of *Blautia*’s cluster (gold rectangles, Control *FL3*, ADHD *FL1*) that *Oscillospira* is negatively correlated with *Blautia*’s entire cluster in Control, and these correlations are completely absent in ADHD.

The lowest level MCNs (Fig 10(E) and 10(F)) also show *Blautia*’s cluster as larger in ADHD, and *Oscillospira*’s cluster as larger in Control. Table 27 contains members of these clusters. *Blautia* and *Oscillospira* each belong to a cluster dominated by its respective family: *Lachnospiraceae* (*FL*), and *Ruminococcaceae* (*FR*). *Oscillospira* is a core *FR* member and at the lowest level, we see the Control FR cluster (with family *Enterobacteriaceae* now a member). *Blautia* is consistently a member of the same cluster as both family *Lachnospiraceae* taxa in ADHD, comparably larger than its *FL3* Control cluster.

**Table 27:** *Blautia* and *Oscillospira* clusters.

			Control			ADHD		
Level	Community	Cluster Type	Cluster	Taxon	Phy	Cluster	Taxon	Phy
**Lowest**	**Firmicutes-dominant,**	**Lachnospiraceae-dominant (FL)**	**FL3**	Blautia 1*		**FL1**	Lachnospiraceae 1	
			**FL3**	Dorea 2		**FL1**	Lachnospiraceae 2*	
			**FL3**	*Bifidobacterium longum*		**FL1**	Coprococcus	
						**FL1**	*Ruminococcus [L]*	
						**FL1**	Blautia 1	
						**FL1**	Dorea 2	
						**FL1**	*Faecalibacterium prausnitzii*	
		**Ruminococcaceae-dominant (FR)**	**FR**	Ruminococcaceae*		**FR**	**Ruminococcaceae**	
			**FR**	**Ruminococcus [R]**		**FR**	**Ruminococcus [R]**	
			**FR**	**Oscillospira**		**FR**	**Oscillospira**	
			**FR**	**Clostridiales 1**		**FR**	Clostridiales 1*	
			**FR**	*Coprobacillus*		**FR**	*Bifidobacterium adolescentis*	
			**FR**	*Enterobacteriaceae*				
			**FR**	*Lachnospira*				

Clusters involving *Blautia* and *Oscillospira*. Phylum: purple = Bacteroidetes, yellow = Firmicutes, brown = Actinobacteria, blue = Proteobacteria.

Heatmaps also indicate increased participation of *Oscillospira*’s cluster (*FR*) in Control (large green rectangle, Fig 11(C)–11(F)), including negative correlations with cluster *BB* that are absent in ADHD, yet another example of reduced ADHD cluster *BB* competition. In the MCNs, Fruchterman-Reingold places cluster *FR* (green) in a much more central position in Control (Fig 10(C) vs 10(D), and 10(E) vs. 10(F)). The negative correlations between *Oscillospira* and *Blautia*’s entire cluster *FL3* (Figs 10(C) and 11(E)) are also evident, almost separating FL3 from the MCN. In ADHD *Blautia*’s cluster *FL1* (gold) occupies a much more central position (Fig 10(D) and 10(F)), with increased ADHD size particularly noticeable at the lowest level (Fig 10(F)).

ATria (Table 28) indicates a general increased importance of *Blautia’s* family (*Lachnospiraceae*) in ADHD, and *Oscillospira’s* family (*Ruminococcaceae*) in Control. A couple of noteworthy taxa follow this trend. Species *Faecalibacterium prausnitzii (Ruminococcaceae)*, an anti-inflammatory bacterium [153] touted as a next-generation probiotic [154], is only ranked in Control. Species *Ruminococcus gnavus* (*Lachnospiraceae*), known to produce an inflammatory polysaccharide [155], is only ranked in ADHD.

**Table 28:** *Lachnospiraceae* and *Ruminococcaceae* rankings.

Phylum	Class	Order	Family	Genus	Lowest Possible
Firmicutes (#2/#T2)	Clostridia (#T3/#T3)	Clostridales (#T2/#3)	*Lachnospiraceae (NR/NR)*	Anaerostipes (#T19/NR)	Anaerostipes (#6/#T26)
				Blautia (#T16/#13)	Blautia 1 (NR/#19)
					Blautia 2 (#9/#T22)
				Coprococcus (NR/#5)	Coprococcus (#T21/#6)
				Dorea (#T14/#12)	Dorea 2 (#13/NR)
				Lachnospira (NR/#16)	Lachnospira (#T27/#5)
				Lachnospiraceae 1 (#19/#15)	Lachnospiraceae 1 (#17/#2)
				Lachnospiraceae 2 (#7/NR)	Lachnospiraceae 2 (#8/#14)
				*Roseburia (NR/NR)*	Roseburia 1 (NR/#T28)
					Roseburia 2(#T25/#T22)
				**Ruminococcus (#1/#11)**	R. gnavus (NR/#12)
			Ruminococcaceae (#4/#T8)	Faecalibacterium (#T16/#2)	F. prausnitzii (#11/NR)
				Oscillospira (#3/#10)	Oscillospira (#5/#9)
				Ruminococcaceae (#8/#9)	Ruminococcaceae (#4/#18)
				Ruminococcus (#2/NR)	Ruminococcus (#T21/NR)

ATria rankings of *Lachnospiraceae* and *Ruminococcaceae* taxa. Dark orange = only ranked in Control, dark purple = only ranked in ADHD, light orange = higher ranked in Control, light purple = higher ranked in ADHD, grey = evenly ranked. **Bold** taxa are ranked #1.

#### Summary

Four clusters were consistently present in both Control and ADHD MCNs. Three are Firmicutes-dominant (*FL*, *FR*, *FT*) and one is Bacteroidetes-dominant (*BB*). Table 29 shows their attributes, and summarizes observations we made about each.

**Table 29:** Largest, consistently present clusters.

Cluster	Attribute	Observation
*BB*	Largest Bacteroidetes-dominant cluster	Larger in ADHD, with more internal cooperation and less external competition.
*FL(1*,*2*,*…)*	Multiple Firmicutes, family *Lachnospiraceae*-dominant clusters	One large, centrally located cluster emerges in ADHD (*FL1*). Others are small, about the same size, and more disconnected (all are this way in Control).
*FR*	Firmicutes, family *Ruminococcaceae*-dominant	Smaller and less centrally located in ADHD.
*FT*	Firmicutes, core member genus *Turicibacter*	Slightly larger in ADHD.

Clusters that we note as being consistently present in Control and ADHD MCNs, and observations.

Table 30 summarizes correlations between members of these clusters. Other than the one exception in ADHD involving genera *Streptococcus* and *Blautia*: *FT* is the only Firmicutes-dominant cluster with taxa positively correlated with Bacteroidetes-dominant cluster (*BB)* members, and all correlations involving *FL*/*FR* (largest Firmicute-dominant clusters) and *BB* taxa are negative. *FT* is completely disconnected from *FL*/*FR* except some ADHD competition. *FL*-*FR* competition only happens in Control.

**Table 30:** Cluster member interactions.

Cluster 1	Cluster 2	Observation
*BB*	*FL*	Always (-), with one exception in ADHD (*Streptococcus*-*Blautia*)
*BB*	*FR*	Always (-)
BB*	*FT*	Always (+)
*FL*	*FR*	Generally (+). Some (-) in Control (all involve either *Ruminococcus* (*FL*) or *Oscillospira* (*FR*))
*FL*	*FT*	Generally disconnected. Some (-) in ADHD (all involve *Phascolarcobacterium* (*FT*))
*FR*	*FT*	Always disconnected.

Interactions between taxa from Table 29 clusters (* = In Control, this took place with *BM1* after the *BB* “split”).

Finally, we summarize taxa (Table 31) and relationships (Table 32) that we noted throughout our analyses.

**Table 31:** Taxa noted throughout our analysis.

Level	Taxon	Observation
*Genus*	*Adlercruetzia*	Role change from Control (Bacteroidetes cooperation) to ADHD (*E*. *dolichum* cooperation). Competition with different Firmicutes (in ADHD,).
*Genus*	*Bacteroides*	Centroid (with nearly 100% connectivity) of cluster *BB* in ADHD.
*Genus*	*Bifidobacterium*	Higher participation in Control (mostly competition). Competition with all Bacteroidetes or Proteobacteria taxa is entirely exclusive to Control, including multiple members and centroid of cluster *BB*. Cooperation (small amount) is entirely exclusive to ADHD.Ranked higher in Control than ADHD nearly 100% of the time, including a #1 ranking at the lowest taxonomic level.
*Genus*	*Coprobacillus*	DESeq2-elevated ADHD taxon, and ranked #1 for ADHD in two MCNs (genus and lowest possible). Competes with multiple *Lachnospiraceae* taxa, including the most abundant.
*Family*	*Enterobacteriaceae*	Involved with Firmicutes-Proteobacteria shifts. Only ranked in ADHD.
*Species*	*F*. *prausnitzii*	Probiotic species only ranked in Control
*Family*	*Lachnospiraceae*	Most abundant family, generally ranked higher in ADHD
*Genus*	*Phascolarctobacterium*	Only *FT* member connected to another Firmicute-dominant cluster (competition). Only ranked in ADHD.
*Family*	*Porphyromonadaceae*	#1 ADHD family, only cluster *BB* member to compete with *FL/FR*.
*Family*	*Rikenellaceae*	Previously reported as ADHD-elevated. Ranked by ATria as important in all six lower level MCNs, and always higher in ADHD.
*Genus*	*Ruminococcus [L]*	#1 Control genus, involved in *FL*-*FR* competition (only observed in Control).
*Species*	*R*. *gnavus*	Produces inflammatory biosaccharide, only ranked in ADHD
*Family*	*Ruminococcaceae*	Second-most abundant family, generally ranked higher in Control
*Genus*	*Turicibacter*	Reported as differentially abundant (Control) by three methods (LEfSe, DESeq and ALDEx2), core member (centroid in ADHD) of *FT*.

Summary of notable taxa throughout our ecological analyses, and observations.

**Table 32:** Relationships noted throughout our analyses.

Taxonomic Level	Relationship(s)	Reason
*Genus*	*Bacteroides*-*Sutterella* (+, both)	Only core correlation consistent across both sample sets at all levels (12 MCNs).
*Genus*	*Bacteroides*-*Prevotella* (-, ADHD)	Only competition involving two Bacteroidetes taxa.
*Genus*	*Bacteroides*-*Streptococcus* (+, ADHD)	*Streptococcus* is one of only two Firmicutes genera to join cluster *BB*, through this connection.
*Genus*	*Butyricimonas*-*Clostridium* (+, ADHD)	*Butyricimonas* was only ranked by ATria in ADHD, and reported as ADHD differentially abundant by two methods (LEfSe and ALDEx2). *Clostridium* is one of only two Firmicutes to join cluster *BB*, through this connection.
*Family-Genus*	*Clostridiaceae*-*Odoricibacter* (-, ADHD)	*Odoribacter* was reported as ADHD-elevated by LEfSe. In ADHD competes with *Clostridiaceae* taxa that compete with multiple cluster *BB* members (including its centroid). *Clostridiaceae* was reported as ADHD-reduced (Control-elevated) by ALDEx2.
*Family-Genus*	*Enterobacteriaceae*-*Oscillospira* (+, Control; -, ADHD)	Only correlation ever to change sign from Control to ADHD. Taxa involved are in the same cluster in Control.
*Genus*	*Blautia*-*Oscillospira* (-, Control) *Streptococcus*-*Blauta* (+, ADHD) *Streptococcus*-*Oscillospira* (-, ADHD)	*Blautia* is associated with obesity and *Oscillospira* with leanness. *Oscillospira* (*FR*) competes with every member of *Blautia*’s cluster (*FL3*) in Control. *FL*-*FR* competition only happens in Control. In ADHD *Streptococcus* cooperates with *Blautia* (obesity) and competes with *Oscillospira* (leanness) *Streptococcus*-*Blautia* is the only time a cluster *BB* member (largest Bacteroidetes-dominant) ever cooperates with taxa from *FL* or *FR* (largest Firmicutes-dominant, collectively over 70% of the population). *Streptococcus* is only ranked in ADHD, *Blautia* is ranked higher in ADHD, *Oscillospira* is ranked higher in Control. *Blautia’*s cluster (*FL1*) is larger and more central in ADHD.). *Oscillospira’s* (*FR*) is larger and more central in Control.

Summary of notable relationships throughout our ecological analyses, and observations.

## Discussion

Traditional analysis methods (i.e. diversity and composition) prevalent in current ADHD gut microbiome literature provide a macroscale representation of a complex ecosystem. Conducting some of these approaches on equal-sized, gender-balanced undergraduate Control and ADHD gut microbiome datasets produced many results that corresponded with this literature, plus a potentially new Control-elevated genus, *Turicibacter* (reported by LEfSe, DESeq2 and ALDEx2). Current literature, as well as our results, suggest this macroscale perspective leaves a largely incomplete picture due to its neglect of underlying complexity. Our goal was to complete more of this picture by venturing deeper, by analyzing two-way ecological relationships (cooperation and competition), plus community detection, and centrality.

Our results provide a deeper meaning to those from the macroscale. Previous differential abundance studies have reported collectively inconclusive results with respect to *Bifidobacterium* [65, 66] and *Bacteroides* [59, 65, 70]. We experienced similar issues with our taxa plots, where anomalous results involving elevated genus *Bifidobacterium* and reduced genera *Bacteroides* and *Sutterella* at ASRS extremes (high and low) imposed significant challenges when interpreting results. Our MCNs estimate that a Bacteroidetes-dominant community (cluster *BB*) forms in both microbiomes, with *Bacteroides* and *Sutterella* both core members, that in ADHD is larger and resides in conditions that favor its cooperation, as opposed to competition in Control. Several cluster BB members have previously been reported as ADHD-elevated, including *Butyricimonas* (reported by two of our differential abundance methods), *Parabacteroides* (for hyperactivity, [59]), *Bacteroides uniformis* [60], *Rikenellaceae* [66], and *Odoribacteriaceae* [68], plus two member descendants (*Bacteroides ovatus* and *Sutterella stercoricanis*, [60]), making its increased size, tightness, and more favorable environment observed in our ADHD MCN interesting. Moreover our MCNs estimate a shift in roles played by *Bacteroides* and *Bifidobacterium* between Control and ADHD microbiomes. In ADHD, *Bacteroides* was estimated as the centroid (driver) taxon for cluster *BB*, having cooperative with nearly every member. And *Bifidobacterium* shifted from exclusively competitive relationships with cluster *BB* members (including its most abundant and centroid) in Control, to exclusively a cooperative relationship in ADHD—with *Bacteroides uniformis*, one of the taxa previously reported as ADHD-elevated [60]. In addition to these relationships with cluster *BB* our MCNs estimated *Bifidobacterium* and its lineages to be much more active in Control gut ecology (24 relationships) compared to ADHD (9 relationships), with ATria ranking *Bifidobacterium* higher in Control more than 85% of the time. Overall, these results suggest potentially significant differential roles played by both *Bifidobacterium* and *Bacteroides* in the Control and ADHD gut ecosystems.

Potential roles played by taxa reported by our differential abundance analysis also became observable. Our MCNs estimated genus *Odoribacter*, reported by our analysis and another [68] as ADHD-elevated, to also compete with two family *Clostridiaceae* taxa, one of which (*Clostridiaceae 1*) was reported by ALDEx2 as ADHD-reduced (subsequently we found, by a factor of two). *Clostridiaceae 1*, in turn, was estimated to compete with multiple taxa from cluster *BB* (much larger in ADHD) in both of our MCNs. Exploring candidate metabolic reactions involved within this triangle may therefore also be important for future experimentation. *Butyricimonas*, reported by two of our differential analysis methods (LEfSe and ALDEx2) as ADHD-elevated, joined cluster BB in ADHD and provided the sole cooperative relationship with *Clostridium*, a taxon otherwise completely disconnected from both MCNs. *Coprobacillus*, reported by DESeq2 as ADHD-elevated, was ranked #1 twice by ATria in the ADHD MCN, which estimated competitive relationships with abundant *Lachnospiraceae* taxa, reported by another study as Control-elevated [68].

New interesting taxa and communities also emerged. Cluster *FT* (cooperative with cluster *BB*) was larger in ADHD. Cluster *FR* (Family *Ruminococcaceae*-dominant, competitive with cluster *BB*) was smaller in ADHD. *Ruminococcaceae* taxa (previously reported as ADHD-reduced [68]) were also almost universally less central in ADHD, including the probiotic *Faecalibacterium prausnitzii* (only ranked by ATria in Control), which was also reported as ADHD-reduced in two other studies [61, 68]. Our MCNs also showed inflammatory polysaccharide *Ruminococcus gnavus* (only ranked by ATria in ADHD), to have ADHD-exclusive competition with *F*. *prausnitzii*. *Ruminococcaceae* member genus *Oscillospira* was also estimated to have ADHD-exclusive competition, with Proteobacteria family *Enterobacteriaceae* (cooperative and fellow *FR* member in Control), and cluster *BB* member Firmicute *Streptococcus*. In particular, the shift in relationship between *Enterobacteriaceae* and *Oscillospira* from cooperation (Control) to competition (ADHD), as well as the dynamic involved in the triangle composed of genera *Streptococcus*, *Blautia*, and *Oscillospira* (Table 32, last row), emerged as noteworthy potential targets for further exploration.

This exploration can add deeper meaning to these results through pursuit of additional studies targeting some of these taxa and relationships, including multi-omics [156] and/or physical laboratory experiments. Fundamentally, ecological relationships manifest through internal interplay within the underlying web of interactions [157]. Cooperation could take place for example if two taxa produce a nutrient that the other consumes; competition could take place if two taxa consume a nutrient that neither produces. Coupling taxa to metabolites they produce and consume and analyzing pathways can help elucidate underlying mechanisms behind these ecological relationships. These pathways can then be searched for neurotransmitters to establish ADHD connections. Clustering could also be performed by functional pathway, enabling a more direct association between microbial genes and specialized biological pathways involving neurotransmitters that influence ADHD.

While results from our analysis could be used as guidance for more in-depth analysis of any ADHD dataset (present or future), it is of course possible (and likely) that changes within attributes of the target population can impact ecological relationships. We are merely laying the groundwork, and with very few studies even attempting this level of analysis [66], an enormous breadth of knowledge remains and many future improvements to our analyses are possible. Published metadata for this dataset was somewhat limited. Future studies involving ADHD and the gut microbiome should account for factors such as BMI [158], demographics [159], ethnicity [160], use of medication/probiotics [61], use of antibiotics [161], diet [162] and gastrointestinal issues [163], age [164], and official diagnosis (beyond ASRS). Additionally although we did strenghten the threshold of our *p*-value for correlations in our MCNs, future work could incorporate multiple hypothesis corrections and sensitivity analysis for ATria for more thorough statistical relevance validation. More meaning to relationships in our MCNs can also be uncovered, through *causality* studies. Causality would give direction to edges, enabling detection of both two- and one-way (i.e. commensalism [139], amensalism [140]) relationships. This can be achieved through for example Bayesian Networks [165], which detect relationships where a taxon is conditionally dependent on another. Conditional dependence also eliminates spurious edges that can occur with correlations; for example, two entities that co-occur with a mutual entity will naturally tend to co-occur [116] (this was also a dependency removed by ATria after finding a central node). Sazal *et al*. [166] have already verified such networks as a predictor for oral microbiome colonization order. Time can also factor into ecological relationships because while sometimes these relationships are constant in microbiomes [167], they can also be transitive [168] or even time-varying [169]. DBNs that account for time have already been used to predict long-term infant gut behavior [170]. Higher-level network metrics such as modularity [171] and vulnerability [172] would provide another potential avenue for comparing and contrasting Control and ADHD MCNs. Amplicon Sequence Variants (ASVs, [173]) can be used in place of the current Operational Taxonomic Units (OTUs) that are generated by similarity-based clustering. ASVs exhibit more reliability at lower levels of the taxonomic tree and can improve the granularity of our MCNs, achieving more species- and sometimes even strain-level classifications.

Also given that ASRS does not offer an official diagnosis and this metadata did provide ASRS scores, an immediate future goal should be a migration from case-control to continuous data analysis with respect to ASRS. Our taxa plots, which observed abundances of core taxa with respect to ASRS score, have already started this process. Some of our other analysis protocols (i.e. alpha- and beta-diversity) will trivially propagate. While differential abundance analysis will ultimately be difficult without explicit sample classifications, networks have potential given some of the strategies mentioned above. For example, multi-omic network techniques such as marginal correlation analysis have already proven useful for correlating heterogeneous datasets [174], making them potentially applicable to estimating correlation between microbial abundance and ASRS scores. Applying Bayesian techniques to such networks could assist in estimating the conditional presence or absence of a microbe-microbe correlation on another dependence (i.e., high or low ASRS score).

A more complete understanding of ADHD and the gut microbiome will best equip the community to make the right decisions when administering treatment(s). Our results, coupled with those in the literature, suggest that the gut microbiota cannot afford to be ignored when it comes to ADHD, and treatments directly targeting the gut microbiome have potential. Encouraging results have been uncovered for gluten and casein-free diets [53], Microbiota Transfer Therapy (MTT, [175, 176]), and probiotics [177] with ASD. Our results also indicate that the gut microbiome is an ecosystem, and any changes to one single element will likely impact other members. Additionally since the human gut microbiome is widely varied across individuals [178], personalized medicine should be used when developing such treatments.

## Supporting information

S1 Fig Class-level taxa plot. Bar plot of taxa relative abundance, at the class level. Samples are ordered on the *x*-axis by increasing ASRS score.
(PNG)

S2 Fig Order-level taxa plot. Bar plot of taxa relative abundance, at the order level. Samples are ordered on the *x*-axis by increasing ASRS score.
(PNG)

S3 Fig Family-level taxa plot. Bar plot of taxa relative abundance, at the family level. Samples are ordered on the *x*-axis by increasing ASRS score.
(PNG)

S4 Fig Taxa plot: lowest possible classification level. Bar plot of taxa relative abundance, using the lowest possible classification level. Samples are ordered on the *x*-axis by increasing ASRS score.
(PNG)

S5 Fig *Bifidobacterium* abundances. Relative abundance plot of *Bifidobacterium*. Samples are ordered on the x-axis by increasing ASRS score.
(PNG)

S6 Fig *Bacteroides* abundances. Relative abundance plot of *Bacteroides*. Samples are ordered on the x-axis by increasing ASRS score.
(PNG)

S7 Fig *Sutterella* abundances. Relative abundance plot of *Sutterella*. Samples are ordered on the x-axis by increasing ASRS score.
(PNG)

S1 Table Experimental and lab methods. Data obtained from the openly available BioProject (PRJNA656791), along with cited protocols [90–92].
(DOCX)

S2 Table Data quality table. Average number of sequences retained after every preprocessing step.
(DOCX)

S3 Table All correlations, all MCNs. Every correlation in all MCNs, grouped by taxonomy. Orange = only found in Control, purple = only found in ADHD, grey = found in both. +(green) = positive correlation, -(red) = negative correlation.
(DOCX)

S4 Table All rankings, all MCNs. ATria rankings of all taxa over all MCNs, grouped by taxonomy. Dark orange = only ranked in Control, dark purple = only ranked in ADHD, light orange = higher ranked in Control, light purple = higher ranked in ADHD, grey = evenly ranked. **Bold** taxa are ranked #1.
(DOCX)

S5 Table All family-level clusters. Communities within Control and ADHD family-level MCNs. Phylum: purple = Bacteroidetes, yellow = Firmicutes, brown = Actinobacteria, blue = Proteobacteria.
(DOCX)

S6 Table All genus-level clusters. Communities within Control and ADHD genus-level MCNs. Phylum: purple = Bacteroidetes, yellow = Firmicutes, brown = Actinobacteria, blue = Proteobacteria.
(DOCX)

S7 Table All clusters, lowest possible taxonomic classification. Communities within Control and ADHD MCNs using the lowest possible level of taxonomic classification. Phylum: purple = Bacteroidetes, yellow = Firmicutes, brown-Actinobacteria, blue = Proteobacteria.
(DOCX)

## References

[1] Polanczyk GV, Salum GA, Sugaya LS, Caye A, Rohde LA. Annual Research Review: A meta-analysis of the worldwide prevalence of mental disorders in children and adolescents. J Child Psychol Psychiatry. 2015 Mar;56(3):345–65. doi: 10.1111/jcpp.12381 25649325

[2] Centers for Disease Control and Prevention (CDC) [Internet]. Available from: http://www.cdc.gov/ncbddd/adhd/data.html

[3] Biederman J, Faraone SV. Attention-deficit hyperactivity disorder. The Lancet. 2005 Jul;366(9481):237–48. 10.1016/S0140-6736(05)66915-2 16023516

[4] Fletcher J, Wolfe B. Long-term consequences of childhood ADHD on criminal activities. J Ment Health Policy Econ. 2009 Sep;12(3):119–38. doi: 10.1186/1478.7547–3–5 19996475 PMC3398051

[5] American Psychiatric Association. Diagnostic and Statistical Manual of Mental Disorders [Internet]. Fifth Edition. American Psychiatric Association; 2013 [cited 2020 Mar 27]. Available from: https://psychiatryonline.org/doi/book/10.1176/appi.books.9780890425596

[6] Woods KE, Danko CM, Chronis-Tuscano A. ADHD in Children and Adolescents. In: Oxford Research Encyclopedia of Psychology [Internet]. Oxford University Press; 2016 [cited 2020 Mar 27]. Available from: https://oxfordre.com/psychology/view/10.1093/acrefore/9780190236557.001.0001/acrefore-9780190236557-e-75

[7] Oliveira CR de, Pedron AC, Gurgel LG, Reppold CT, Fonseca RP. Executive functions and sustained attention:Comparison between age groups of 19–39 and 40–59 years old. Dement neuropsychol. 2012 Mar;6(1):29–34. doi: 10.1590/S1980-57642012DN06010005 29213769 PMC5619104

[8] Castellanos FX, Proal E. Large-scale brain systems in ADHD: beyond the prefrontal–striatal model. Trends in Cognitive Sciences. 2012 Jan;16(1):17–26. doi: 10.1016/j.tics.2011.11.007 22169776 PMC3272832

[9] Rubia K. Cognitive Neuroscience of Attention Deficit Hyperactivity Disorder (ADHD) and Its Clinical Translation. Front Hum Neurosci. 2018 Mar 29;12:100. doi: 10.3389/fnhum.2018.00100 29651240 PMC5884954

[10] Arnsten AF, Dudley AG. Methylphenidate improves prefrontal cortical cognitive function through Alpha-2 adrenoceptor and Dopamine D1 receptor actions: Relevance to therapeutic effects in Attention Deficit Hyperactivity Disorder. Behav Brain Funct. 2005;1(1):2. doi: 10.1186/1744-9081-1-2 15916700 PMC1143775

[11] Larsson H, Chang Z, D’Onofrio BM, Lichtenstein P. The heritability of clinically diagnosed attention deficit hyperactivity disorder across the lifespan. Psychol Med. 2014 Jul;44(10):2223–9. doi: 10.1017/S0033291713002493 24107258 PMC4071160

[12] Luckey TD. Introduction to intestinal microecology. The American Journal of Clinical Nutrition. 1972 Dec 1;25(12):1292–4. doi: 10.1093/ajcn/25.12.1292 4639749

[13] Sender R, Fuchs S, Milo R. Revised estimates for the number of human and bacteria cells in the body. PLoS Biol. 2016 Aug 19;14(8):e1002533. doi: 10.1371/journal.pbio.1002533 27541692 PMC4991899

[14] Manor O, Levy R, Borenstein E. Mapping the inner workings of the microbiome: Genomic- and metagenomic-based study of metabolism and metabolic interactions in the human microbiome. Cell Metabolism. 2014 Nov;20(5):742–52. doi: 10.1016/j.cmet.2014.07.021 25176148 PMC4252837

[15] Martin CR, Osadchiy V, Kalani A, Mayer EA. The Brain-gut-microbiome Axis. Cellular and Molecular Gastroenterology and Hepatology. 2018;6(2):133–48. doi: 10.1016/j.jcmgh.2018.04.003 30023410 PMC6047317

[16] Romijn JA, Corssmit EP, Havekes LM, Pijl H. Gut-brain axis. Current Opinion in Clinical Nutrition and Metabolic Care. 2008 Jul;11(4):518–21. doi: 10.1097/MCO.0b013e328302c9b0 18542016

[17] Rhee SH, Pothoulakis C, Mayer EA. Principles and clinical implications of the brain–gut–enteric microbiota axis. Nat Rev Gastroenterol Hepatol. 2009 May;6(5):306–14. doi: 10.1038/nrgastro.2009.35 19404271 PMC3817714

[18] Bravo JA, Forsythe P, Chew MV, Escaravage E, Savignac HM, Dinan TG, et al. Ingestion of *Lactobacillus* strain regulates emotional behavior and central GABA receptor expression in a mouse via the vagus nerve. Proc Natl Acad Sci USA. 2011 Sep 20;108(38):16050–5. 21876150 10.1073/pnas.1102999108 PMC3179073

[19] Dinan TG, Stilling RM, Stanton C, Cryan JF. Collective unconscious: How gut microbes shape human behavior. J Psychiatr Res. 2015 Apr;63:1–9. doi: 10.1016/j.jpsychires.2015.02.021 25772005

[20] Rea K, Dinan TG, Cryan JF. The microbiome: A key regulator of stress and neuroinflammation. Neurobiology of Stress. 2016 Oct;4:23–33. doi: 10.1016/j.ynstr.2016.03.001 27981187 PMC5146205

[21] Ming X, Chen N, Ray C, Brewer G, Kornitzer J, Steer RA. A gut feeling: A hypothesis of the role of the microbiome in Attention-Deficit/Hyperactivity Disorders. Child Neurology Open. 2018 Jan;5:2329048X1878679. doi: 10.1177/2329048X18786799 30023407 PMC6047248

[22] Duel BP, Steinberg-Epstein R, Hill M, Lerner M. A survey of voiding dysfunction in children with Attention Deficit-Hyperactivity Disorder. Journal of Urology. 2003 Oct;170(4 Part 2):1521–4. doi: 10.1097/01.ju.0000091219.46560.7b 14501650

[23] McKeown C, Hisle-Gorman E, Eide M, Gorman GH, Nylund CM. Association of constipation and fecal incontinence with Attention-Deficit/Hyperactivity Disorder. PEDIATRICS. 2013 Nov 1;132(5):e1210–5. doi: 10.1542/peds.2013-1580 24144702 PMC4530301

[24] Wang LJ, Li SC, Li SW, Kuo HC, Lee SY, Huang LH, et al. Gut microbiota and plasma cytokine levels in patients with attention-deficit/hyperactivity disorder. Transl Psychiatry. 2022 Feb 23;12(1):76. doi: 10.1038/s41398-022-01844-x 35197458 PMC8866486

[25] Yang LL, Stiernborg M, Skott E, Gillberg T, Landberg R, Giacobini M, et al. Lower plasma concentrations of short-chain fatty acids (SCFAs) in patients with ADHD. Journal of Psychiatric Research. 2022 Dec;156:36–43. doi: 10.1016/j.jpsychires.2022.09.042 36228390

[26] Silva YP, Bernardi A, Frozza RL. The Role of Short-Chain Fatty Acids From Gut Microbiota in Gut-Brain Communication. Front Endocrinol. 2020 Jan 31;11:25. doi: 10.3389/fendo.2020.00025 32082260 PMC7005631

[27] Dalile B, Van Oudenhove L, Vervliet B, Verbeke K. The role of short-chain fatty acids in microbiota–gut–brain communication. Nat Rev Gastroenterol Hepatol. 2019 Aug;16(8):461–78. doi: 10.1038/s41575-019-0157-3 31123355

[28] Galland L. The gut microbiome and the brain. Journal of Medicinal Food. 2014 Dec;17(12):1261–72. doi: 10.1089/jmf.2014.7000 25402818 PMC4259177

[29] Pettersson G. The neural control of the serotonin content in mammalian enterochromaffin cells. Acta Physiol Scand Suppl. 1979;470:1–30. 229694

[30] Luczynski P, Whelan SO, O’Sullivan C, Clarke G, Shanahan F, Dinan TG, et al. Adult microbiota-deficient mice have distinct dendritic morphological changes: Differential effects in the amygdala and hippocampus. Gaspar P, editor. Eur J Neurosci. 2016 Nov;44(9):2654–66. doi: 10.1111/ejn.13291 27256072 PMC5113767

[31] Clarke G, Grenham S, Scully P, Fitzgerald P, Moloney RD, Shanahan F, et al. The microbiome-gut-brain axis during early life regulates the hippocampal serotonergic system in a sex-dependent manner. Mol Psychiatry. 2013 Jun;18(6):666–73. doi: 10.1038/mp.2012.77 22688187

[32] Jiang H, Ling Z, Zhang Y, Mao H, Ma Z, Yin Y, et al. Altered fecal microbiota composition in patients with major depressive disorder. Brain, Behavior, and Immunity. 2015 Aug;48:186–94. doi: 10.1016/j.bbi.2015.03.016 25882912

[33] Roman P, Rueda-Ruzafa L, Cardona D, Cortes-Rodríguez A. Gut–brain axis in the executive function of autism spectrum disorder: Behavioural Pharmacology. 2018 Oct;29(7):654–63. 10.1097/FBP.0000000000000428 30179883

[34] Floresco SB, Magyar O. Mesocortical dopamine modulation of executive functions: Beyond working memory. Psychopharmacology. 2006 Oct 6;188(4):567–85. doi: 10.1007/s00213-006-0404-5 16670842

[35] Heijtz RD, Wang S, Anuar F, Qian Y, Bjorkholm B, Samuelsson A, et al. Normal gut microbiota modulates brain development and behavior. Proceedings of the National Academy of Sciences. 2011 Feb 15;108(7):3047–52. 10.1073/pnas.1010529108 PMC3041077 21282636

[36] Needham BD, Tang W, Wu WL. Searching for the gut microbial contributing factors to social behavior in rodent models of autism spectrum disorder: Gut microbiota and social behavior. Devel Neurobio. 2018 May;78(5):474–99. 10.1002/dneu.22581 29411548

[37] Li Q, Han Y, Dy ABC, Hagerman RJ. The gut microbiota and autism spectrum disorders. Front Cell Neurosci. 2017 Apr 28;11:120. doi: 10.3389/fncel.2017.00120 28503135 PMC5408485

[38] Russell J, editor. Autism as an executive disorder. Oxford; New York: Oxford University Press; 1997. 313 p.

[39] Parracho HM, Bingham MO, Gibson GR, McCartney AL. Differences between the gut microflora of children with autistic spectrum disorders and that of healthy children. Journal of Medical Microbiology. 2005 Oct 1;54(10):987–91. doi: 10.1099/jmm.0.46101-0 16157555

[40] Park AJ, Collins J, Blennerhassett PA, Ghia JE, Verdu EF, Bercik P, et al. Altered colonic function and microbiota profile in a mouse model of chronic depression. Neurogastroenterol Motil. 2013 Sep;25(9):733–e575. doi: 10.1111/nmo.12153 23773726 PMC3912902

[41] Desbonnet L, Clarke G, Traplin A, O’Sullivan O, Crispie F, Moloney RD, et al. Gut microbiota depletion from early adolescence in mice: Implications for brain and behaviour. Brain, Behavior, and Immunity. 2015 Aug;48:165–73. doi: 10.1016/j.bbi.2015.04.004 25866195

[42] Wong ML, Inserra A, Lewis MD, Mastronardi CA, Leong L, Choo J, et al. Inflammasome signaling affects anxiety- and depressive-like behavior and gut microbiome composition. Mol Psychiatry. 2016 Jun;21(6):797–805. doi: 10.1038/mp.2016.46 27090302 PMC4879188

[43] Inserra A, Rogers GB, Licinio J, Wong ML. The microbiota-inflammasome hypothesis of major depression. BioEssays. 2018 Sep;40(9):1800027. doi: 10.1002/bies.201800027 30004130 PMC12180310

[44] Aizawa E, Tsuji H, Asahara T, Takahashi T, Teraishi T, Yoshida S, et al. Possible association of *Bifidobacterium* and *Lactobacillus* in the gut microbiota of patients with major depressive disorder. Journal of Affective Disorders. 2016 Sep;202:254–7. 27288567 10.1016/j.jad.2016.05.038

[45] Chen J jun, Zheng P, Liu Y yun, Zhong X gang, Wang H yang, Guo Y jie, et al. Sex differences in gut microbiota in patients with major depressive disorder. NDT. 2018 Feb;Volume 14:647–55. 10.2147/NDT.S159322 PMC5833751 29520144

[46] Meyer C, Vassar M. The fragility of probiotic *Bifidobacterium longum* NCC3001 use for depression in patients with Irritable Bowel Syndrome. Gastroenterology. 2018 Feb;154(3):764. 10.1053/j.gastro.2017.09.055 29352957

[47] Messaoudi M, Lalonde R, Violle N, Javelot H, Desor D, Nejdi A, et al. Assessment of psychotropic-like properties of a probiotic formulation (*Lactobacillus helveticus* R0052 and *Bifidobacterium longum* R0175) in rats and human subjects. Br J Nutr. 2011 Mar 14;105(5):755–64. 20974015 10.1017/S0007114510004319

[48] Goldstein S, Schwebach AJ. The comorbidity of Pervasive Developmental Disorder and Attention Deficit Hyperactivity Disorder: results of a retrospective chart review. J Autism Dev Disord. 2004 Jun;34(3):329–39. doi: 10.1023/b:jadd.0000029554.46570.68 15264500

[49] Reiersen AM, Todd RD. Co-occurrence of ADHD and autism spectrum disorders: phenomenology and treatment. Expert Rev Neurother. 2008 Apr;8(4):657–69. doi: 10.1586/14737175.8.4.657 18416666

[50] Gadow KD, DeVincent CJ, Pomeroy J. ADHD symptom subtypes in children with pervasive developmental disorder. J Autism Dev Disord. 2006 Feb;36(2):271–83. doi: 10.1007/s10803-005-0060-3 16477513

[51] Mayes SD, Calhoun SL, Mayes RD, Molitoris S. Autism and ADHD: Overlapping and discriminating symptoms. Research in Autism Spectrum Disorders. 2012 Jan;6(1):277–85.

[52] Doherty JL, Owen MJ. Genomic insights into the overlap between psychiatric disorders: Implications for research and clinical practice. Genome Med. 2014;6(4):29. doi: 10.1186/gm546 24944580 PMC4062063

[53] Grimaldi R, Gibson GR, Vulevic J, Giallourou N, Castro-Mejía JL, Hansen LH, et al. A prebiotic intervention study in children with autism spectrum disorders (ASDs). Microbiome. 2018 Dec;6(1):133. doi: 10.1186/s40168-018-0523-3 30071894 PMC6091020

[54] Mathee K, Cickovski T, Deoraj A, Stollstorff M, Narasimhan G. The gut microbiome and neuropsychiatric disorders: implications for attention deficit hyperactivity disorder (ADHD). Journal of Medical Microbiology. 2020 Jan 1;69(1):14–24. doi: 10.1099/jmm.0.001112 31821133 PMC7440676

[55] Checa-Ros A, Jeréz-Calero A, Molina-Carballo A, Campoy C, Muñoz-Hoyos A. Current Evidence on the Role of the Gut Microbiome in ADHD Pathophysiology and Therapeutic Implications. Nutrients. 2021 Jan 16;13(1):249. doi: 10.3390/nu13010249 33467150 PMC7830868

[56] Kalenik A, Kardaś K, Rahnama A, Sirojć K, Wolańczyk T. Gut microbiota and probiotic therapy in ADHD: A review of current knowledge. Progress in Neuro-Psychopharmacology and Biological Psychiatry. 2021 Aug;110:110277. doi: 10.1016/j.pnpbp.2021.110277 33561522

[57] Sukmajaya AC, Lusida MI, Soetjipto, Setiawati Y. Systematic review of gut microbiota and attention-deficit hyperactivity disorder (ADHD). Ann Gen Psychiatry. 2021 Dec;20(1):12. doi: 10.1186/s12991-021-00330-w 33593384 PMC7888126

[58] Shannon CE. A Mathematical Theory of Communication. Bell System Technical Journal. 1948 Jul;27(3):379–423.

[59] Prehn-Kristensen A, Zimmermann A, Tittmann L, Lieb W, Schreiber S, Baving L, et al. Reduced microbiome alpha diversity in young patients with ADHD. Yao YG, editor. PLoS ONE. 2018 Jul 12;13(7):e0200728. doi: 10.1371/journal.pone.0200728 30001426 PMC6042771

[60] Wang LJ, Yang CY, Chou WJ, Lee MJ, Chou MC, Kuo HC, et al. Gut microbiota and dietary patterns in children with attention-deficit/hyperactivity disorder. Eur Child Adolesc Psychiatry. 2020 Mar;29(3):287–97. doi: 10.1007/s00787-019-01352-2 31119393

[61] Jiang HY, Zhou YY, Zhou GL, Li YC, Yuan J, Li XH, et al. Gut microbiota profiles in treatment-naïve children with attention deficit hyperactivity disorder. Behav Brain Res. 2018 16;347:408–13. 29580894 10.1016/j.bbr.2018.03.036

[62] Casas L, Karvonen AM, Kirjavainen PV, Täubel M, Hyytiäinen H, Jayaprakash B, et al. Early life home microbiome and hyperactivity/inattention in school-age children. Sci Rep. 2019 Dec;9(1):17355. doi: 10.1038/s41598-019-53527-1 31758010 PMC6874766

[63] Szopinska-Tokov J, Dam S, Naaijen J, Konstanti P, Rommelse N, Belzer C, et al. Investigating the gut microbiota composition of individuals with Attention-Deficit/Hyperactivity Disorder and association with symptoms. Microorganisms. 2020 Mar 13;8(3). doi: 10.3390/microorganisms8030406 32183143 PMC7143990

[64] Kruskal JB. Multidimensional scaling by optimizing goodness of fit to a nonmetric hypothesis. Psychometrika. 1964 Mar;29(1):1–27.

[65] Pärtty A, Kalliomäki M, Wacklin P, Salminen S, Isolauri E. A possible link between early probiotic intervention and the risk of neuropsychiatric disorders later in childhood: A randomized trial. Pediatr Res. 2015 Jun;77(6):823–8. doi: 10.1038/pr.2015.51 25760553

[66] Aarts E, Ederveen THA, Naaijen J, Zwiers MP, Boekhorst J, Timmerman HM, et al. Gut microbiome in ADHD and its relation to neural reward anticipation. Hashimoto K, editor. PLoS ONE. 2017 Sep 1;12(9):e0183509. doi: 10.1371/journal.pone.0183509 28863139 PMC5581161

[67] Cheng S, Han B, Ding M, Wen Y, Ma M, Zhang L, et al. Identifying psychiatric disorder-associated gut microbiota using microbiota-related gene set enrichment analysis. Briefings in Bioinformatics. 2020 May 21;21(3):1016–22. doi: 10.1093/bib/bbz034 30953055

[68] Wan L, Ge WR, Zhang S, Sun YL, Wang B, Yang G. Case-Control Study of the Effects of Gut Microbiota Composition on Neurotransmitter Metabolic Pathways in Children With Attention Deficit Hyperactivity Disorder. Front Neurosci. 2020 Feb 18;14:127. doi: 10.3389/fnins.2020.00127 32132899 PMC7040164

[69] Uffelmann E, Huang QQ, Munung NS, de Vries J, Okada Y, Martin AR, et al. Genome-wide association studies. Nat Rev Methods Primers. 2021 Aug 26;1(1):59.

[70] Stevens AJ, Purcell RV, Darling KA, Eggleston MJF, Kennedy MA, Rucklidge JJ. Human gut microbiome changes during a 10 week Randomised Control Trial for micronutrient supplementation in children with attention deficit hyperactivity disorder. Sci Rep. 2019 Dec;9(1):10128. doi: 10.1038/s41598-019-46146-3 31300667 PMC6625977

[71] DuPaul GJ, Power TJ, Anastopoulos AD, Reid R. ADHD Rating Scale—IV: Checklists, norms, and clinical interpretation. New York, NY, US: Guilford Press; 1998. viii, 79 p. (ADHD Rating Scale—IV: Checklists, norms, and clinical interpretation.).

[72] Coyte KZ, Rao C, Rakoff-Nahoum S, Foster KR. Ecological rules for the assembly of microbiome communities. Gordo I, editor. PLoS Biol. 2021 Feb 19;19(2):e3001116. doi: 10.1371/journal.pbio.3001116 33606675 PMC7946185

[73] Bassler BL, Greenberg EP, Stevens AM. Cross-species induction of luminescence in the quorum-sensing bacterium *Vibrio harveyi*. Journal of bacteriology. 1997;179(12):4043–5. 9190823 10.1128/jb.179.12.4043-4045.1997 PMC179216

[74] Xavier KB, Bassler BL. Regulation of uptake and processing of the quorum-sensing autoinducer AI-2 in *Escherichia coli*. JB. 2005 Jan 1;187(1):238–48. 10.1128/JB.187.1.238-248.2005 PMC538819 15601708

[75] Engelmoer DJP, Behm JE, Toby Kiers E. Intense competition between arbuscular mycorrhizal mutualists in an *in vitro* root microbiome negatively affects total fungal abundance. Mol Ecol. 2014 Mar;23(6):1584–93. 24050702 10.1111/mec.12451

[76] Lê Cao KA, Boitard S, Besse P. Sparse PLS discriminant analysis: Biologically relevant feature selection and graphical displays for multiclass problems. BMC Bioinformatics. 2011 Dec;12(1):253. doi: 10.1186/1471-2105-12-253 21693065 PMC3133555

[77] Segata N, Izard J, Waldron L, Gevers D, Miropolsky L, Garrett WS, et al. Metagenomic biomarker discovery and explanation. Genome Biol. 2011;12(6):R60. doi: 10.1186/gb-2011-12-6-r60 21702898 PMC3218848

[78] Love MI, Huber W, Anders S. Moderated estimation of fold change and dispersion for RNA-seq data with DESeq2. Genome Biol. 2014 Dec;15(12):550. doi: 10.1186/s13059-014-0550-8 25516281 PMC4302049

[79] Fernandes AD, Macklaim JM, Linn TG, Reid G, Gloor GB. ANOVA-Like Differential Expression (ALDEx) Analysis for Mixed Population RNA-Seq. Parkinson J, editor. PLoS ONE. 2013 Jul 2;8(7):e67019. doi: 10.1371/journal.pone.0067019 23843979 PMC3699591

[80] Lin H, Peddada SD. Analysis of compositions of microbiomes with bias correction. Nat Commun. 2020 Dec;11(1):3514. doi: 10.1038/s41467-020-17041-7 32665548 PMC7360769

[81] Caporaso JG, Kuczynski J, Stombaugh J, Bittinger K, Bushman FD, Costello EK, et al. QIIME allows analysis of high-throughput community sequencing data. Nat Methods. 2010 May;7(5):335–6. doi: 10.1038/nmeth.f.303 20383131 PMC3156573

[82] Tasnim N, Abulizi N, Pither J, Hart MM, Gibson DL. Linking the Gut Microbial Ecosystem with the Environment: Does Gut Health Depend on Where We Live? Front Microbiol. 2017 Oct 6;8:1935. doi: 10.3389/fmicb.2017.01935 29056933 PMC5635058

[83] Fernandez M, Riveros JD, Campos M, Mathee K, Narasimhan G. Microbial “social networks.” BMC Genomics. 2015;16(Suppl 11):S6. 10.1186/1471-2164-16-S11-S6 PMC4652466 26576770

[84] Faust K, Sathirapongsasuti JF, Izard J, Segata N, Gevers D, Raes J, et al. Microbial co-occurrence relationships in the human microbiome. Ouzounis CA, editor. PLoS Comput Biol. 2012 Jul 12;8(7):e1002606. doi: 10.1371/journal.pcbi.1002606 22807668 PMC3395616

[85] Frey BJ, Dueck D. Clustering by passing messages between data points. Science. 2007 Feb 16;315(5814):972–6. doi: 10.1126/science.1136800 17218491

[86] Cickovski T, Peake E, Aguiar-Pulido V, Narasimhan G. ATria: A novel centrality algorithm applied to biological networks. BMC Bioinformatics. 2017 Jun;18(S8):239. 28617231 10.1186/s12859-017-1659-z PMC5471957

[87] Cickovski T, Narasimhan G. Constructing lightweight and flexible pipelines using Plugin-Based Microbiome Analysis (PluMA). Bioinformatics. 2018 Sep 1;34(17):2881–8. doi: 10.1093/bioinformatics/bty198 29618009 PMC6129309

[88] Rösler M, Casas M, Konofal E, Buitelaar J. Attention deficit hyperactivity disorder in adults. The World Journal of Biological Psychiatry. 2010 Aug;11(5):684–98. doi: 10.3109/15622975.2010.483249 20521876

[89] Goodrich JK, Di Rienzi SC, Poole AC, Koren O, Walters WA, Caporaso JG, et al. Conducting a microbiome study. Cell. 2014 Jul;158(2):250–62. doi: 10.1016/j.cell.2014.06.037 25036628 PMC5074386

[90] Kozich JJ, Westcott SL, Baxter NT, Highlander SK, Schloss PD. Development of a dual-index sequencing strategy and curation pipeline for analyzing amplicon sequence data on the MiSeq Illumina sequencing platform. Appl Environ Microbiol. 2013 Sep 1;79(17):5112–20. doi: 10.1128/AEM.01043-13 23793624 PMC3753973

[91] 16S Metagenomic Sequencing Library Preparation: Preparing 16S Ribosomal RNA Gene Amplicons for the Illumina MiSeq System [Internet]. 2021 [cited 2023 Apr 18]. Available from: https://support.illumina.com/content/dam/illumina-support/documents/documentation/chemistry_documentation/16s/16s-metagenomic-library-prep-guide-15044223-b.pdf

[92] Edgar RC, Haas BJ, Clemente JC, Quince C, Knight R. UCHIME improves sensitivity and speed of chimera detection. Bioinformatics. 2011 Aug 15;27(16):2194–200. doi: 10.1093/bioinformatics/btr381 21700674 PMC3150044

[93] Mirzayi C, Renson A, Genomic Standards Consortium, Massive Analysis and Quality Control Society, Furlanello C, Sansone SA, et al. Reporting guidelines for human microbiome research: the STORMS checklist. Nat Med. 2021 Nov;27(11):1885–92. doi: 10.1038/s41591-021-01552-x 34789871 PMC9105086

[94] Cureton EE. Tetrachoric Correlation by the Camp Approximation. Educational and Psychological Measurement. 1968 Jul;28(2):239–44.

[95] Kessler RC, Adler LA, Gruber MJ, Sarawate CA, Spencer T, Van Brunt DL. Validity of the World Health Organization Adult ADHD Self-Report Scale (ASRS) Screener in a representative sample of health plan members. Int J Methods Psychiatr Res. 2007 Jun;16(2):52–65. doi: 10.1002/mpr.208 17623385 PMC2044504

[96] Brevik EJ, Lundervold AJ, Haavik J, Posserud M. Validity and accuracy of the Adult Attention‐Deficit/Hyperactivity Disorder (ADHD) Self‐Report Scale (ASRS) and the Wender Utah Rating Scale (WURS) symptom checklists in discriminating between adults with and without ADHD. Brain Behav [Internet]. 2020 Jun [cited 2023 Apr 9];10(6). Available from: doi: 10.1002/brb3.1605 32285644 PMC7303368

[97] Bolger AM, Lohse M, Usadel B. Trimmomatic: A flexible trimmer for Illumina sequence data. Bioinformatics. 2014 Aug 1;30(15):2114–20. doi: 10.1093/bioinformatics/btu170 24695404 PMC4103590

[98] John J. SeqPrep [Internet]. 2019. Available from: https://github.com/jstjohn/SeqPrep

[99] Edgar RC. Search and clustering orders of magnitude faster than BLAST. Bioinformatics. 2010 Oct 1;26(19):2460–1. doi: 10.1093/bioinformatics/btq461 20709691

[100] Cole JR, Wang Q, Fish JA, Chai B, McGarrell DM, Sun Y, et al. Ribosomal Database Project: data and tools for high throughput rRNA analysis. Nucl Acids Res. 2014 Jan;42(D1):D633–42. doi: 10.1093/nar/gkt1244 24288368 PMC3965039

[101] DeSantis TZ, Hugenholtz P, Larsen N, Rojas M, Brodie EL, Keller K, et al. Greengenes, a chimera-checked 16S rRNA gene database and workbench compatible with ARB. Applied and Environmental Microbiology. 2006 Jul 1;72(7):5069–72. doi: 10.1128/AEM.03006-05 16820507 PMC1489311

[102] Chao A. Species Estimation and Applications. In: Kotz S, Read CB, Balakrishnan N, Vidakovic B, Johnson NL, editors. Encyclopedia of Statistical Sciences [Internet]. Hoboken, NJ, USA: John Wiley & Sons, Inc.; 2006 [cited 2021 Jan 6]. p. ess5051. Available from: http://doi.wiley.com/10.1002/0471667196.ess5051

[103] Bonferroni C. Teoria statistica delle classi e calcolo delle probabilità. Pubblicazioni del R Istituto Superiore di Scienze Economiche e Commerciali di Firenze 8: 3–62.—Open Access Library [Internet]. 2020 [cited 2020 Jan 24]. Google Scholar. 1936;

[104] Lozupone C, Lladser ME, Knights D, Stombaugh J, Knight R. UniFrac: an effective distance metric for microbial community comparison. ISME J. 2011 Feb;5(2):169–72. doi: 10.1038/ismej.2010.133 20827291 PMC3105689

[105] Excoffier L, Smouse PE, Quattro JM. Analysis of molecular variance inferred from metric distances among DNA haplotypes: application to human mitochondrial DNA restriction data. Genetics. 1992 Jun 1;131(2):479–91. doi: 10.1093/genetics/131.2.479 1644282 PMC1205020

[106] Barker M, Rayens W. Partial least squares for discrimination. J Chemometrics. 2003 Mar 24;17(3):166–73.

[107] Ruiz-Perez D, Guan H, Madhivanan P, Mathee K, Narasimhan G. So you think you can PLS-DA? In: 2018 IEEE 8th International Conference on Computational Advances in Bio and Medical Sciences (ICCABS) [Internet]. Las Vegas, NV: IEEE; 2018 [cited 2020 Apr 3]. p. 1–1. Available from: https://ieeexplore.ieee.org/document/8542038/

[108] Quinn TP, Erb I, Gloor G, Notredame C, Richardson MF, Crowley TM. A field guide for the compositional analysis of any-omics data. GigaScience. 2019 Sep 1;8(9):giz107. doi: 10.1093/gigascience/giz107 31544212 PMC6755255

[109] Nearing JT, Douglas GM, Hayes MG, MacDonald J, Desai DK, Allward N, et al. Microbiome differential abundance methods produce different results across 38 datasets. Nat Commun. 2022 Jan 17;13(1):342. doi: 10.1038/s41467-022-28034-z 35039521 PMC8763921

[110] Benjamini Y, Hochberg Y. Controlling the False Discovery Rate: A Practical and Powerful Approach to Multiple Testing. Journal of the Royal Statistical Society: Series B (Methodological). 1995 Jan;57(1):289–300.

[111] Holm S. A Simple Sequentially Rejective Multiple Test Procedure. Scandinavian Journal of Statistics. 1979;6:65–70.

[112] Friedman J, Alm EJ. Inferring Correlation Networks from Genomic Survey Data. von Mering C, editor. PLoS Comput Biol. 2012 Sep 20;8(9):e1002687. doi: 10.1371/journal.pcbi.1002687 23028285 PMC3447976

[113] Shannon P. Cytoscape: A software environment for integrated models of biomolecular interaction networks. Genome Research. 2003 Nov 1;13(11):2498–504. doi: 10.1101/gr.1239303 14597658 PMC403769

[114] Fruchterman TMJ, Reingold EM. Graph drawing by force-directed placement. Softw: Pract Exper. 1991 Nov;21(11):1129–64.

[115] Jackson MO, Wolinsky A. A strategic model of social and economic networks. Journal of Economic Theory. 1996 Oct;71(1):44–74.

[116] Easley D, Kleinberg J. Networks, crowds, and markets: Reasoning about a highly connected world. New York: Cambridge University Press; 2010. 727 p.

[117] Toro‐Valdivieso C, Toro F, Stubbs S, Castro‐Nallar E, Blacklaws B. Patterns of the fecal microbiota in the Juan Fernández fur seal (*Arctocephalus philippii*). MicrobiologyOpen [Internet]. 2021 Aug [cited 2023 Jul 25];10(4). Available from: https://onlinelibrary.wiley.com/doi/10.1002/mbo3.1215 10.1002/mbo3.1215 PMC8302013 34459554

[118] Salonen A, Salojärvi J, Lahti L, De Vos WM. The adult intestinal core microbiota is determined by analysis depth and health status. Clinical Microbiology and Infection. 2012 Jul;18:16–20. doi: 10.1111/j.1469-0691.2012.03855.x 22647042

[119] Lam TJ, Ye Y. Meta-analysis of microbiome association networks reveal patterns of dysbiosis in diseased microbiomes. Sci Rep. 2022 Oct 19;12(1):17482. doi: 10.1038/s41598-022-22541-1 36261472 PMC9581956

[120] Weiss S, Van Treuren W, Lozupone C, Faust K, Friedman J, Deng Y, et al. Correlation detection strategies in microbial data sets vary widely in sensitivity and precision. ISME J. 2016 Jul;10(7):1669–81. doi: 10.1038/ismej.2015.235 26905627 PMC4918442

[121] Klecka WR. Discriminant analysis. Beverly Hills, Calif: Sage Publications; 1980. 71 p. (Quantitative applications in the social sciences).

[122] Fisher RA. The use of multiple measurements in taxonomic problems. Annals of Eugenics. 1936 Sep;7(2):179–88.

[123] Fung TC, Vuong HE, Luna CDG, Pronovost GN, Aleksandrova AA, Riley NG, et al. Intestinal serotonin and fluoxetine exposure modulate bacterial colonization in the gut. Nat Microbiol. 2019 Dec;4(12):2064–73. doi: 10.1038/s41564-019-0540-4 31477894 PMC6879823

[124] Nikolas M, Friderici K, Waldman I, Jernigan K, Nigg JT. Gene × environment interactions for ADHD: Synergistic effect of 5HTTLPR genotype and youth appraisals of inter-parental conflict. Behav Brain Funct. 2010;6(1):23. 20398347 10.1186/1744-9081-6-23 PMC2865439

[125] Xue R, Zhang H, Pan J, Du Z, Zhou W, Zhang Z, et al. Peripheral dopamine controlled by gut microbes inhibits invariant natural killer T cell-mediated Hepatitis. Front Immunol. 2018 Oct 17;9:2398. doi: 10.3389/fimmu.2018.02398 30386344 PMC6199378

[126] Aitchison J. The statistical analysis of compositional data. Caldwell, N.J: Blackburn Press; 2003. 1 p.

[127] D’Argenio V, Salvatore F. The role of the gut microbiome in the healthy adult status. Clin Chim Acta. 2015 Dec 7;451(Pt A):97–102. doi: 10.1016/j.cca.2015.01.003 25584460

[128] Bukin YuS, Galachyants YuP, Morozov IV, Bukin SV, Zakharenko AS, Zemskaya TI. The effect of 16S rRNA region choice on bacterial community metabarcoding results. Sci Data. 2019 Mar;6(1):190007. doi: 10.1038/sdata.2019.7 30720800 PMC6362892

[129] Picard C, Fioramonti J, Francois A, Robinson T, Neant F, Matuchansky C. Review article: bifidobacteria as probiotic agents—physiological effects and clinical benefits. Aliment Pharmacol Ther. 2005 Sep;22(6):495–512. doi: 10.1111/j.1365-2036.2005.02615.x 16167966

[130] Russell DA, Ross RP, Fitzgerald GF, Stanton C. Metabolic activities and probiotic potential of bifidobacteria. International Journal of Food Microbiology. 2011 Sep;149(1):88–105. doi: 10.1016/j.ijfoodmicro.2011.06.003 21763022

[131] Sharma M, Wasan A, Sharma RK. Recent developments in probiotics: An emphasis on Bifidobacterium. Food Bioscience. 2021 Jun;41:100993.

[132] Zavaglia AG, Kociubinski G, Pérez P, De Antoni G. Isolation and Characterization of Bifidobacterium Strains for Probiotic Formulation. Journal of Food Protection. 1998 Jul 1;61(7):865–73. doi: 10.4315/0362-028x-61.7.865 9678171

[133] Invernici MM, Salvador SL, Silva PHF, Soares MSM, Casarin R, Palioto DB, et al. Effects of *Bifidobacterium* probiotic on the treatment of chronic periodontitis: A randomized clinical trial. J Clin Periodontol. 2018 Oct;45(10):1198–210. 30076613 10.1111/jcpe.12995 PMC6221043

[134] Usta-Gorgun B, Yilmaz-Ersan L. Short-chain fatty acids production by Bifidobacterium species in the presence of salep. Electronic Journal of Biotechnology. 2020 Sep;47:29–35.

[135] Wexler AG, Goodman AL. An insider’s perspective: *Bacteroides* as a window into the microbiome. Nat Microbiol. 2017 May;2(5):17026. 28440278 10.1038/nmicrobiol.2017.26 PMC5679392

[136] Mason BL, Li Q, Minhajuddin A, Czysz AH, Coughlin LA, Hussain SK, et al. Reduced anti-inflammatory gut microbiota are associated with depression and anhedonia. Journal of Affective Disorders. 2020 Apr;266:394–401. doi: 10.1016/j.jad.2020.01.137 32056905

[137] Wang L, Christophersen CT, Sorich MJ, Gerber JP, Angley MT, Conlon MA. Increased abundance of *Sutterella* spp. and *Ruminococcus torques* in feces of children with autism spectrum disorder. Mol Autism. 2013;4(1):42. 24188502 10.1186/2040-2392-4-42 PMC3828002

[138] Craig F, Lamanna AL, Margari F, Matera E, Simone M, Margari L. Overlap between Autism Spectrum Disorders and Attention Deficit Hyperactivity Disorder: Searching for distinctive/common clinical features: Overlap between ASD and ADHD. Autism Research. 2015 Jun;8(3):328–37. 25604000 10.1002/aur.1449 PMC4654237

[139] Telesford K, Ochoa-Repáraz J, Kasper LH. Gut commensalism, cytokines, and central nervous system demyelination. Journal of Interferon & Cytokine Research. 2014 Aug;34(8):605–14. doi: 10.1089/jir.2013.0134 25084177 PMC4118718

[140] Sahney S, Benton MJ, Ferry PA. Links between global taxonomic diversity, ecological diversity and the expansion of vertebrates on land. Biol Lett. 2010 Aug 23;6(4):544–7. doi: 10.1098/rsbl.2009.1024 20106856 PMC2936204

[141] Jafari M, Ansari-Pour N. Why, When and How to Adjust Your P Values? Cell J [Internet]. 2018 Aug [cited 2023 Apr 12];20(4). Available from: doi: 10.22074/cellj.2019.5992 30124010 PMC6099145

[142] Di Leo G, Sardanelli F. Statistical significance: p value, 0.05 threshold, and applications to radiomics—reasons for a conservative approach. Eur Radiol Exp. 2020 Dec;4(1):18. doi: 10.1186/s41747-020-0145-y 32157489 PMC7064671

[143] Neumann NM, Plastino A, Pinto Junior JA, Freitas AA. Is *p* -value 0.05 enough? *A study on the statistical evaluation of classifiers*. The Knowledge Engineering Review. 2020;36:e1.

[144] Dancey CP, Reidy J. Statistics without maths for psychology. Pearson Education; 2007.

[145] Martinson JNV, Pinkham NV, Peters GW, Cho H, Heng J, Rauch M, et al. Rethinking gut microbiome residency and the Enterobacteriaceae in healthy human adults. ISME J. 2019 Sep;13(9):2306–18. doi: 10.1038/s41396-019-0435-7 31089259 PMC6776003

[146] Garrett WS, Gallini CA, Yatsunenko T, Michaud M, DuBois A, Delaney ML, et al. Enterobacteriaceae Act in Concert with the Gut Microbiota to Induce Spontaneous and Maternally Transmitted Colitis. Cell Host & Microbe. 2010 Sep;8(3):292–300. doi: 10.1016/j.chom.2010.08.004 20833380 PMC2952357

[147] Liu J, Yan Q, Luo F, Shang D, Wu D, Zhang H, et al. Acute cholecystitis associated with infection of Enterobacteriaceae from gut microbiota. Clinical Microbiology and Infection. 2015 Sep;21(9):851.e1–851.e9. doi: 10.1016/j.cmi.2015.05.017 26025761

[148] Stecher B, Denzler R, Maier L, Bernet F, Sanders MJ, Pickard DJ, et al. Gut inflammation can boost horizontal gene transfer between pathogenic and commensal Enterobacteriaceae. Proceedings of the National Academy of Sciences. 2012 Jan 24;109(4):1269–74. doi: 10.1073/pnas.1113246109 22232693 PMC3268327

[149] Gophna U, Konikoff T, Nielsen HB. *Oscillospira* and related bacteria—From metagenomic species to metabolic features: Metabolism of Oscillospira. Environ Microbiol. 2017 Mar;19(3):835–41. 28028921 10.1111/1462-2920.13658

[150] Konikoff T, Gophna U. Oscillospira: a Central, Enigmatic Component of the Human Gut Microbiota. Trends in Microbiology. 2016 Jul;24(7):523–4. doi: 10.1016/j.tim.2016.02.015 26996766

[151] Ozato N, Saito S, Yamaguchi T, Katashima M, Tokuda I, Sawada K, et al. Blautia genus associated with visceral fat accumulation in adults 20–76 years of age. npj Biofilms Microbiomes. 2019 Dec;5(1):28. doi: 10.1038/s41522-019-0101-x 31602309 PMC6778088

[152] Cortese S. The association between ADHD and obesity: Intriguing, progressively more investigated, but still puzzling. Brain Sciences. 2019 Sep 27;9(10):256. doi: 10.3390/brainsci9100256 31569608 PMC6826981

[153] Sokol H, Pigneur B, Watterlot L, Lakhdari O, Bermudez-Humaran LG, Gratadoux JJ, et al. Faecalibacterium prausnitzii is an anti-inflammatory commensal bacterium identified by gut microbiota analysis of Crohn disease patients. Proceedings of the National Academy of Sciences. 2008 Oct 28;105(43):16731–6. doi: 10.1073/pnas.0804812105 18936492 PMC2575488

[154] He X, Zhao S, Li Y. Faecalibacterium prausnitzii: A Next-Generation Probiotic in Gut Disease Improvement. Chen T, editor. Canadian Journal of Infectious Diseases and Medical Microbiology. 2021 Mar 5;2021:1–10.

[155] Henke MT, Kenny DJ, Cassilly CD, Vlamakis H, Xavier RJ, Clardy J. *Ruminococcus gnavus*, a member of the human gut microbiome associated with Crohn’s disease, produces an inflammatory polysaccharide. Proc Natl Acad Sci USA. 2019 Jun 25;116(26):12672–7. 31182571 10.1073/pnas.1904099116 PMC6601261

[156] Aguiar-Pulido V, Huang W, Suarez-Ulloa V, Cickovski T, Mathee K, Narasimhan G. Metagenomics, metatranscriptomics, and metabolomics approaches for microbiome analysis: Supplementary issue: Bioinformatics methods and applications for big metagenomics data. Evolutionary Bioinformatics. 2016 Jan;12s1:EBO.S36436. 10.4137/EBO.S36436 PMC4869604 27199545

[157] Ponomarova O, Patil KR. Metabolic interactions in microbial communities: Untangling the Gordian knot. Current Opinion in Microbiology. 2015 Oct;27:37–44. doi: 10.1016/j.mib.2015.06.014 26207681

[158] Aoun A, Darwish F, Hamod N. The Influence of the Gut Microbiome on Obesity in Adults and the Role of Probiotics, Prebiotics, and Synbiotics for Weight Loss. pnf. 2020 Jun 30;25(2):113–23. doi: 10.3746/pnf.2020.25.2.113 32676461 PMC7333005

[159] The Milieu Intérieur Consortium, Scepanovic P, Hodel F, Mondot S, Partula V, Byrd A, et al. A comprehensive assessment of demographic, environmental, and host genetic associations with gut microbiome diversity in healthy individuals. Microbiome. 2019 Dec;7(1):130. doi: 10.1186/s40168-019-0747-x 31519223 PMC6744716

[160] Gaulke CA, Sharpton TJ. The influence of ethnicity and geography on human gut microbiome composition. Nat Med. 2018 Oct;24(10):1495–6. doi: 10.1038/s41591-018-0210-8 30275567

[161] Iizumi T, Battaglia T, Ruiz V, Perez Perez GI. Gut Microbiome and Antibiotics. Archives of Medical Research. 2017 Nov;48(8):727–34. doi: 10.1016/j.arcmed.2017.11.004 29221800

[162] David LA, Maurice CF, Carmody RN, Gootenberg DB, Button JE, Wolfe BE, et al. Diet rapidly and reproducibly alters the human gut microbiome. Nature. 2014 Jan;505(7484):559–63. doi: 10.1038/nature12820 24336217 PMC3957428

[163] Verlaet AAJ, Noriega DB, Hermans N, Savelkoul HFJ. Nutrition, immunological mechanisms and dietary immunomodulation in ADHD. Eur Child Adolesc Psychiatry. 2014 Jul;23(7):519–29. doi: 10.1007/s00787-014-0522-2 24493267

[164] Pallikkuth S, Mendez R, Russell K, Sirupangi T, Kvistad D, Pahwa R, et al. Age Associated Microbiome and Microbial Metabolites Modulation and Its Association With Systemic Inflammation in a Rhesus Macaque Model. Front Immunol. 2021 Oct 19;12:748397. doi: 10.3389/fimmu.2021.748397 34737748 PMC8560971

[165] Pearl J. Causality: models, reasoning, and inference. Cambridge, U.K.; New York: Cambridge University Press; 2000. 384 p.

[166] Sazal MR, Ruiz-Perez D, Cickovski T, Narasimhan G. Inferring relationships in microbiomes from Signed Bayesian Networks. In: 2018 IEEE 8th International Conference on Computational Advances in Bio and Medical Sciences (ICCABS) [Internet]. Las Vegas, NV: IEEE; 2018 [cited 2020 Mar 27]. p. 1–1. Available from: https://ieeexplore.ieee.org/document/8542086/

[167] Bäckhed F, Fraser CM, Ringel Y, Sanders ME, Sartor RB, Sherman PM, et al. Defining a healthy human gut microbiome: Current concepts, future directions, and clinical applications. Cell Host & Microbe. 2012 Nov;12(5):611–22. doi: 10.1016/j.chom.2012.10.012 23159051

[168] Brown K, DeCoffe D, Molcan E, Gibson DL. Diet-Induced Dysbiosis of the Intestinal Microbiota and the Effects on Immunity and Disease. Nutrients. 2012 Aug 21;4(8):1095–119. doi: 10.3390/nu4081095 23016134 PMC3448089

[169] Cho I, Blaser MJ. The human microbiome: At the interface of health and disease. Nat Rev Genet. 2012 Apr;13(4):260–70. doi: 10.1038/nrg3182 22411464 PMC3418802

[170] McGeachie MJ, Sordillo JE, Gibson T, Weinstock GM, Liu YY, Gold DR, et al. Longitudinal prediction of the infant gut microbiome with dynamic Bayesian networks. Sci Rep. 2016 Apr;6(1):20359. doi: 10.1038/srep20359 26853461 PMC4745046

[171] Newman MEJ, Girvan M. Finding and evaluating community structure in networks. Phys Rev E. 2004 Feb 26;69(2):026113. doi: 10.1103/PhysRevE.69.026113 14995526

[172] Bozzo E, Franceschet M, Rinaldi F. Vulnerability and power on networks. Net Sci. 2015 Jun;3(2):196–226.

[173] Callahan BJ, McMurdie PJ, Holmes SP. Exact sequence variants should replace operational taxonomic units in marker-gene data analysis. ISME J. 2017 Dec;11(12):2639–43. doi: 10.1038/ismej.2017.119 28731476 PMC5702726

[174] Heintz-Buschart A, May P, Laczny CC, Lebrun LA, Bellora C, Krishna A, et al. Integrated multi-omics of the human gut microbiome in a case study of familial type 1 diabetes. Nat Microbiol. 2017 Jan;2(1):16180. 10.1038/nmicrobiol.2016.180 27723761

[175] Kang DW, Adams JB, Gregory AC, Borody T, Chittick L, Fasano A, et al. Microbiota Transfer Therapy alters gut ecosystem and improves gastrointestinal and autism symptoms: An open-label study. Microbiome. 2017 Dec;5(1):10. doi: 10.1186/s40168-016-0225-7 28122648 PMC5264285

[176] Adams J, Krajmalnik-Brown R, Kang D, Sadowski M, Khoruts A, Borody T. Method for treating Autism Spectrum Disorder and associated symptoms. 20190134144, 2019.

[177] Shaaban SY, El Gendy YG, Mehanna NS, El-Senousy WM, El-Feki HSA, Saad K, et al. The role of probiotics in children with autism spectrum disorder: A prospective, open-label study. Nutritional Neuroscience. 2018 Oct 21;21(9):676–81. doi: 10.1080/1028415X.2017.1347746 28686541

[178] Flint HJ. Variability and Stability of the Human Gut Microbiome. In: Why Gut Microbes Matter [Internet]. Cham: Springer International Publishing; 2020 [cited 2021 Jun 13]. p. 63–79. (Fascinating Life Sciences). Available from: http://link.springer.com/10.1007/978-3-030-43246-1_6

