# Supplementary figures and images for: Attention Deficit Hyperactivity Disorder (ADHD) and the gut microbiome: An ecological perspective
Source: PLoS One. 2023 Aug 18;18(8):e0273890. doi: 10.1371/journal.pone.0273890 (PMC10437823; doi:10.1371/journal.pone.0273890)

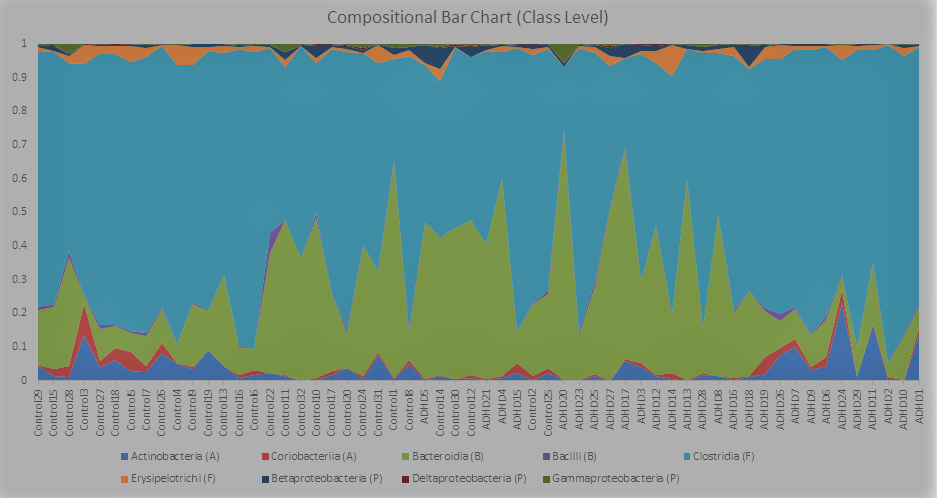

Supplement: S1 Fig — Bar plot of taxa relative abundance, at the class level. Samples are ordered on the x-axis by increasing ASRS score. (PNG) [file pone.0273890.s001.png]

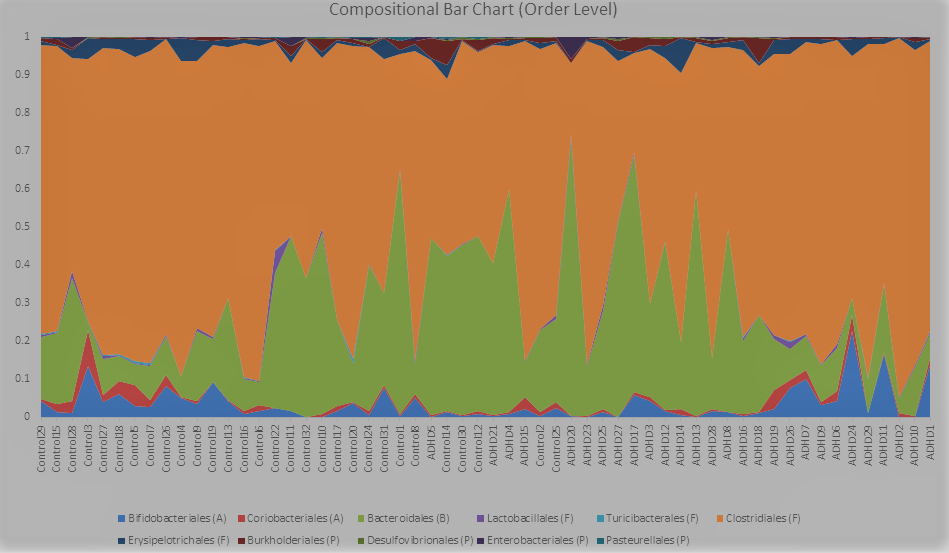

Supplement: S2 Fig — Bar plot of taxa relative abundance, at the order level. Samples are ordered on the x-axis by increasing ASRS score. (PNG) [file pone.0273890.s002.png]

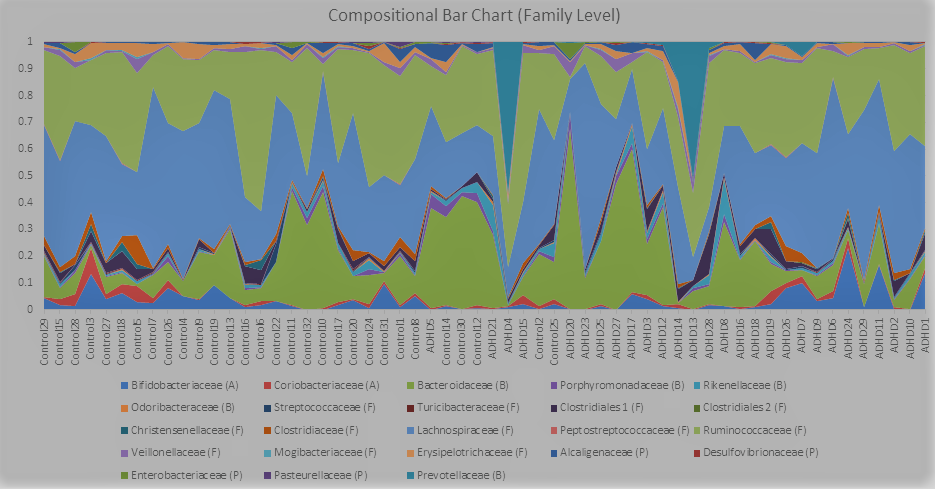

Supplement: S3 Fig — Bar plot of taxa relative abundance, at the family level. Samples are ordered on the x-axis by increasing ASRS score. (PNG) [file pone.0273890.s003.png]

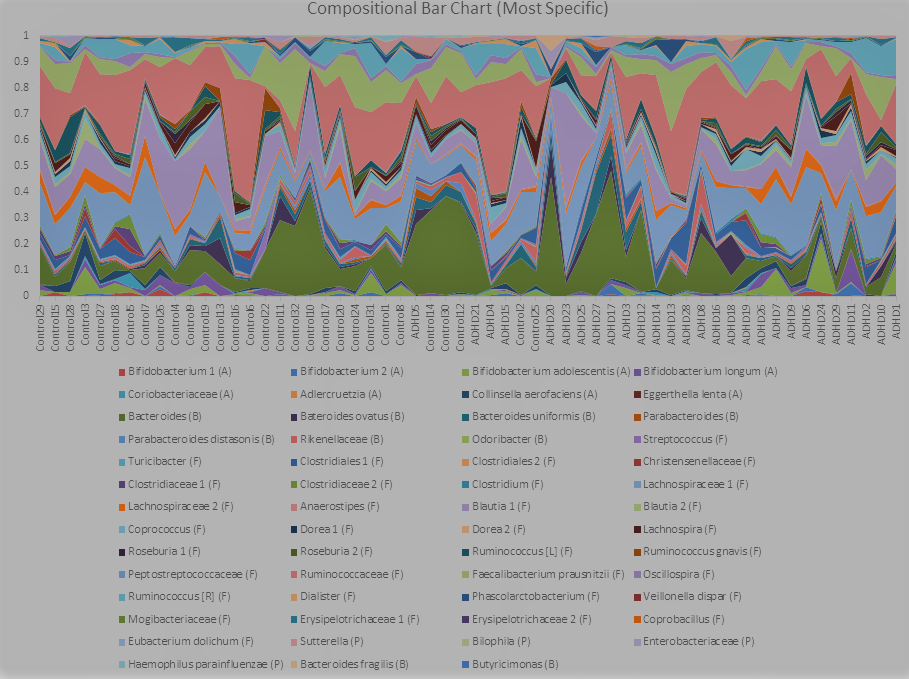

Supplement: S4 Fig — Bar plot of taxa relative abundance, using the lowest possible classification level. Samples are ordered on the x-axis by increasing ASRS score. (PNG) [file pone.0273890.s004.png]

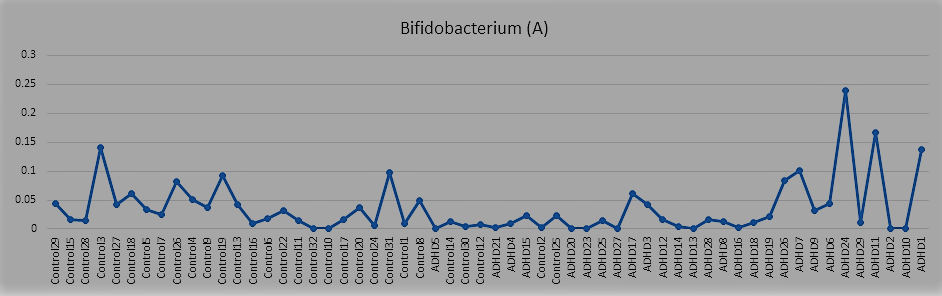

Supplement: S5 Fig — Relative abundance plot of Bifidobacterium. Samples are ordered on the x-axis by increasing ASRS score. (PNG) [file pone.0273890.s005.png]

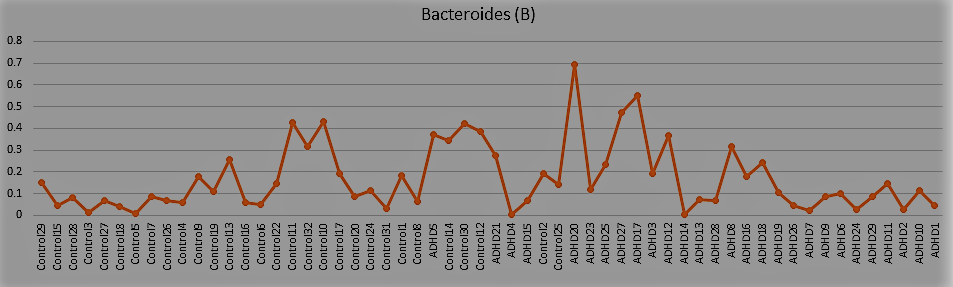

Supplement: S6 Fig — Relative abundance plot of Bacteroides. Samples are ordered on the x-axis by increasing ASRS score. (PNG) [file pone.0273890.s006.png]

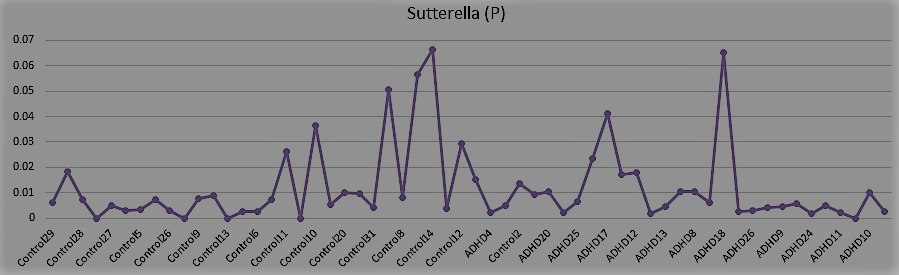

Supplement: S7 Fig — Relative abundance plot of Sutterella. Samples are ordered on the x-axis by increasing ASRS score. (PNG) [file pone.0273890.s007.png]
