# Supplementary material for: Attention Deficit Hyperactivity Disorder (ADHD) and the gut microbiome: An ecological perspective
Source: PLoS One. 2023 Aug 18;18(8):e0273890. doi: 10.1371/journal.pone.0273890 (PMC10437823; doi:10.1371/journal.pone.0273890)
Supplement: S1 Table — Data obtained from the openly available BioProject (PRJNA656791), along with cited protocols [90–92]. (DOCX) [file pone.0273890.s008.docx]

| **Item** | **Comment(s)** |
| --- | --- |
| Study Design | Observational, Case-Control (No Blinding) |
| Participants | Undergraduate students  Total of 577 volunteered to complete ASRS  Volunteers split into Control and Case (ADHD) based on ASRS score. ADHD defined as having ASRS score of 17 or above on at least one of the subscales (hyperactivity or inattention)  Gut microbiome sampling performed on a randomly selected group from both sets (32 control, 29 case) |
| Geographic Location | Miami, FL USA |
| Relevant Dates | Non-longitudinal, provided one date (submission to NCBI, 12-08-2020) |
| Eligiblity Criteria | No inclusion/exclusion data provided, we assume none |
| Antibiotics Usage | No antibiotics exclusion data provided, we assume none |
| Analytic Sample Size | Released 61 samples, recommended excluding 3 based on their own outlier analysis (we followed this recommendation), producing 58 samples.  Power Size (Confidence Interval 95%, computed using EpiInfo version 7.2.5, STATCALC package): 88% |
| Longitudinal Studies | N/A, not longitudinal |
| Matching | N/A, not matched |
| Ethics | FIU Office Of Research Integrity verbally waived IRB consent, dataset was publicly available and in fully deidentified form, no master key provided |
| Laboratory Methods | Lab: GeneWiz, Inc. following protocols of (Kozich *et al*, 2013) and Illumina 16S Metagenomics Sequencing Library Preparation (<https://support.illumina.com/content/dam/illumina-support/documents/documentation/chemistry_documentation/16s/16s-metagenomic-library-prep-guide-15044223-b.pdf>). Made a slight modification that controlled PCR cycles to prevent chimera formation. |
| Specimen Collection | Host Body Site: Feces (UBERON:0001988)  Collection Device: Omnigene-Gut OMR-200 stool sample collection kit |
| Shipping | Length of time (collection-to-receipt): 3 days  No temperature control specified but OMR-200 is designed to avoid this cost and maintains integrity at -20-50 degrees C (-4 to 112 degrees F) |
| Storage | Turnaround time (collection to sequencing): 1 business day  No refrigeration explicitly mentioned, but OMR-200 maintains integrity at room temperature for up to 60 days (<https://www.dnagenotek.com/row/pdf/PD-PR-01036.pdf>)  Reagent Kit v2 (500 cycles), reagant cartridge and HT1 (hybridization buffer) stored at -15 to -25 degrees C, incorporation sbuffer and single-use flow cell stored at 2 to 8 degrees C.  Cartridge thawed in room temperature deionized water |
| DNA Extraction | Kit: Zymo Research DNA MiniPrep Kit  Technology: Zymo-Spin Column/Plate  Method: Bead-beat, spin-column purification, PCR inhibitor filter |
| Human DNA sequence depletion or microbial DNA enrichment | Microbial DNA enrichment |
| Primer selection | Primers:  313f (CCTACGGGNGGCWGCAG) and 806r (GACTACHVGGGTATCTAATCC)  Variable Region: 16S (v3-4)  Amplification Method: PCR |
| Positive controls | No mock communities mentioned  10% PhiX mix used (denaturation with 0.1 N NaOH, dilution with 10 mM Tris-Cl, pH 8.5 with 0.1% Tween 20, 1 minute centrifuge at 280 xg, subsequent dilution with HT1) |
| Negative controls | Two no-template controls, 200 microliters pure H_2_O |
| Contaminant Mitigation/Identification | Segregated pre- and post-PCR areas with pass-through air locks, separate equipment/supplies including Personal Protective Equipment (PPE), dressing areas, refrigerator/freezer, sink, and water purification systems  Areas regularly cleaned daily with fresh cleaner (10% bleach), thoroughly cleaned weekly  MiSeq clearance requirements met (> 61cm above, > 61cm each side, > 10.2 cm behind), cleaned with laboratory-grade water  Flow cell glass cleaned with alcohol (70%) wipes, stage with low-lint lab tissue, assembly with laboratory-grade water  Unidirectional pre-to-post PCR workflow  Pre- and post-PCR reagants sorted in pre-PCR area before any transfer to post-PCR  Aerosol/liquid resistant pippetting tips, changed between samples  Post-run washes performed after every sequencing run  Negative control had Ct > 37  Chimeric sequences removed with USearch v. 6.1 (Edgar *et al*, 2011) |
| Replication | No biological or technical replicates mentioned, we assume none |
| Sequencing Strategy | Amplicon Sequencing  Variable Region: 16S (v3-4)  Sequencer: Illumina MiSeq |
| Sequencing Methods | Experimental Quantification (QMP)  Minimum read length=302bp (Average not specified)  Minimum sequencing depth = 2000 (Average not specified)  Paired reads |
| Batch Effects | Randomly sampled 50,000 sequences for each sample |
| Metatranscriptomics | N/A |
| Metaproteomics | N/A |
| Metabolomics | N/A |
