# Supplementary material for: Attention Deficit Hyperactivity Disorder (ADHD) and the gut microbiome: An ecological perspective
Source: PLoS One. 2023 Aug 18;18(8):e0273890. doi: 10.1371/journal.pone.0273890 (PMC10437823; doi:10.1371/journal.pone.0273890)
Supplement: S2 Table — Average number of sequences retained after every preprocessing step. (DOCX) [file pone.0273890.s009.docx]

| **Pairwise Raw** | **Chimera** | **Size Filter** | **Subsample** | **Singleton** | **Prevalence** |
| --- | --- | --- | --- | --- | --- |
| 44209 | 43867 | 37011 | 35506 | 34095 | 30898 |
