# Supplementary material for: Attention Deficit Hyperactivity Disorder (ADHD) and the gut microbiome: An ecological perspective
Source: PLoS One. 2023 Aug 18;18(8):e0273890. doi: 10.1371/journal.pone.0273890 (PMC10437823; doi:10.1371/journal.pone.0273890)
Supplement: S3 Table — Every correlation in all MCNs, grouped by taxonomy. Orange = only found in Control, purple = only found in ADHD, grey = found in both. +(green) = positive correlation, -(red) = negative correlation. (DOCX) [file pone.0273890.s010.docx]

| **Phylum** | | | **Class** | | **Order** | | **Family** | | **Genus** | | **Lowest Possible** | |  |
| --- | --- | --- | --- | --- | --- | --- | --- | --- | --- | --- | --- | --- | --- |
| Actinobacteria-Bacteroidetes | - | | Actinobacteria- Bacteroidia | - | Bifidobacteriales-Bacteroidales | - | Bifidobacteriaceae -Bacteroidaceae | - | Bifidobactrium -Bacteroides | - | B. adolescentis –  B. ovatus | - |  |
|  |  |  |  |  |  |  |  |  |  |  | B. longum – B.uniformis | + |  |
|  |  |  |  |  |  |  | Bifidobacteriaceae -Porphyromonadaceae | - | Bifidobacterium - Parabacteroides | - |  | |  |
|  |  |  | Coriobacteria-Bacteroidia | - | Coriobacteriales -Bacteroidales | - | Coriobacteriaceae -Bacteroidaceae | - | Adlercreutzia- Bacteroides | + | Adlercreutzia- B.uniformis | + |  |
|  |  |  |  |  |  |  |  |  | Adlercreutiza-Parabacteroides | + | Adlercreutzia- P.distasonis | + |  |
|  |  |  |  |  |  |  |  |  | Adlercreutzia- Odoribacter | + |  | |  |
|  |  |  |  |  |  |  |  |  | Collinsella- Bacteroides | - | C. aerofaciens – B. uniformis | - |  |
|  |  |  |  |  |  |  |  |  | *Collinsella - Butyricimonas* | | C. aerofaciens – Butyricimonas | - |  |
|  |  |  |  |  |  |  |  |  | *Eggtherella-Bacteroides* | | E. lenta – B. ovatus | - |  |
|  |  |  |  |  |  |  |  |  | Eggerthella- Odoribacter | - | E. lenta- Odoribacter | - |  |
|  | | | | | | | | | | | | |  |
| Actinobacteria Proteobacteria | - | | Actinobacteria -Betaproteobacteria | - | *Bifidobacteriales-Burkholderiales* | | *Bifidobacteriaceae-Alcaligenaceae* | | Bifidobacterium –  Sutterella | - |  | |  |
|  |  |  | *Actinobacteria-Gammaproteobacteria* | | *Bifidobacteriales- Enterobacteriales* | | *Bifidobacteriaceae-Enterobacteriaceae* | | *Bifidobacterium-Enterobacteriaceae* | | Bifidobacterium 2 – Enterobacteriaceae | - |  |
|  |  |  | Coriobacteria -Deltaproteobacteria | - |  | | | | | | | |  |
|  | | | | | | | | | | | | |  |
| *Actinobacteria- Firmicutes* | | | *Actinobacteria- Bacilli* | | *Bifidobacteriales-Turicibacteriales* | | *Bifidobacteriaceae-Turicibacteriaceae* | | *Bifidobacterium-Turicibacter* | | Bifidobacterium 1 – Turicibacter | + |  |
|  |  |  | *Actinobacteria-Clostridia* | | Bifidobacteriales -Clostridiales | + | Bifidobacteriaceae- Lachnospiraceae | + | Bifidobacterium -Lachnospiraceae 2 | + | Bifidobacterium 1 – Blautia 2 | - |  |
|  |  |  |  |  |  |  |  |  |  |  | Bifidobacterium 1 – Ruminococcus [L] | + |  |
|  |  |  |  |  |  |  |  |  |  |  | B. longum –  Blautia 1 | + |  |
|  |  |  |  |  |  |  | *Bifidobacteriales – Clostridiales* | | *Bifidobacterium-Clostridiales* | | B. adolescentis – Clostridiales 1 | + |  |
|  |  |  |  |  |  |  |  |  | *Bifidobacterium-Ruminococcaceae* | | Bifidobacterium 2 – Ruminococcaceae | - |  |
|  |  |  |  |  |  |  |  |  |  |  | B. longum – Oscillospira | - |  |
|  |  |  |  |  |  |  | *Bifidobacteriaceae – Veillonellaceae* | | *Bifidobacterium-Veillonella* | | Bifidobacterium 2 – Dialister | - |  |
|  |  |  |  |  | *Coriobacteriales- Costridiales* | | Coriobacteriaceae – Clostridiales 1 | + | Coriobacteriaceae - Christinellaceae | + | Coriobacteriaceae - Christinellaceae | + |  |
|  |  |  |  |  |  |  | Coriobacteriaceae- Clostridiaceae | + | *Coriobacteriaceae-Clostridiaceae* | | Coriobacteriaceae- Clostridiaceae 2 | + |  |
|  |  |  |  |  |  |  | *Coriobacteriaceae- Erysipelotrichaceae-* | | Adlercreutzia- Eubacterium | *+* | Adlercreutzia-  E. dolicum | + |  |
|  |  |  |  |  |  |  | *Coriobacteriaceae- Lachnospiraceae* | | Adlercreutzia -Blautia | - |  | |  |
|  |  |  |  |  |  |  |  |  | Adlercreutzia - Lachnospira | - | Adlercreutzia - Lachnospira | - |  |
|  |  |  |  |  |  |  |  |  | Adlercreutzia - Lachnospiraceae 1 | - |  | |  |
|  |  |  |  |  |  |  |  |  | Adlercreutzia - Lachnospiraceae 2 |  | Adlercreutzia - Lachnospiraceae 2 |  |  |
|  |  |  |  |  |  |  |  |  | Coriobacteriaceae -Lachnospiraceae 1 | - | Coriobacteriaceae -Lachnospiraceae 1 | - |  |
|  |  |  |  |  |  |  |  |  | Eggerthella-Dorea | - |  | |  |
|  |  |  |  |  |  |  |  |  | Eggerthella- Lachnospiraceae 1 | - | E. lenta –  Roseburia 2 | + |  |
|  |  |  |  |  |  |  | *Coriobacteriaceae- Ruminococcaceae* | | Adlercreutzia – Ruminococcus [R] | - | Adlercreutzia – Ruminococcus [R] | - |  |
|  | | | | | | | | | | | | |  |
| *Actinobacteria-Actinobacteria* | | | *Actinobacteria-Actinobacteria* | | *Bifidobacteriales - Bifidobacteriales* | | *Bifidobacteriaceae - Bifidobacteriaceae* | | *Bifidobacterium-Bifidobacterium* | | Bifidobacterium 2 – B. adolescentis | - |  |
|  |  |  | *Actinobacteria-Coriobacteria* | | Bifidobacteriales-Coriobacteriales | + | Bifidobacteriaceae-Coriobacteriaceae | + | *Bifidobacterium-Adlercruetzia* | | Bifidobacterium 1 -Adlercruetzia | + |  |
|  |  |  |  |  |  |  |  |  | Bifidobacterium-Collinsella | + |  | |  |
|  |  |  |  |  |  |  |  |  | *Bifidobacterium-Eggerthella* | | B. longum – E.lenta | - |  |
|  |  |  | *Coriobacteria - Coriobacteria* | | *Coriobacteriales - Coriobacteriales* | | *Coriobacteriaceae-Coriobacteriaceae* | | Adlercruetzia-Coriobacteriaceae | + |  | |  |
|  | | | | | | | | | | | | |  |
| Bacteroidetes-Firmicutes | - | | *Bacteroidia -Bacilli* | | *Bacteroidales Lactobacillales* | | Bacteroidaceae Streptococcaceae | + | Bacteroides -Streptococcus | + |  | |  |
|  |  |  |  |  |  |  | *Porphyromonadaceae Turicibacteriaceae-* | | Parabacteroides -Turicibacter | + | Parabacteroides-Turicibacter | + |  |
|  |  |  |  |  |  |  |  |  | Parabacteroides -Turicibacter | + | P.distasonis -Turicibacter | + |  |
|  |  |  | *Bacteroidia - Clostridia* | | Bacteroidales - Clostridiales | - | Bacteroidaceae – Clostridiaceae | - | Bacteroides – Clostridiaceae 1 | - | Bacteroides -Clostridiaceae 1 | - |  |
|  |  |  |  |  |  |  |  |  |  |  | B. ovatus – Clostridiales 1 | - |  |
|  |  |  |  |  |  |  |  |  |  |  | B. uniformis - Clostridiaceae 1 | - |  |
|  |  |  |  |  |  |  |  |  | Bacteroides – Clostridiaceae 2 | - | B. uniformis – Clostridiaceae 2 | - |  |
|  |  |  |  |  |  |  | *Bacteroidaceae -Lachnospiraceae* | | *Bacteroides-Lachnospiraceae* | | B. uniformis -Lachnospiraceae 2 | - |  |
|  |  |  |  |  |  |  |  |  |  |  | Bacteroides -Roseburia 1 | - |  |
|  |  |  |  |  |  |  | *Bacteroidaceae -Ruminococcaceae* | | *Bacteroides-Ruminococcaceae* | | B. ovatus- Ruminococcus [R] | - |  |
|  |  |  |  |  |  |  |  |  |  |  | Bacteroides- Ruminococcaceae | - |  |
|  |  |  |  |  |  |  | *Odoribacteriaceae -Clostridiaceae* | | Butyricimonas -Clostridium | + |  | |  |
|  |  |  |  |  |  |  |  |  | Odoribacter – Clostridiaceae 1 | - | Odoribacter - Clostridiaceae 1 | - |  |
|  |  |  |  |  |  |  |  |  | Odoribacter – Clostridiaceae 2 | - |  | |  |
|  |  |  |  |  |  |  | *Odoribacteriaceae -Ruminococcaceae* | | Odoribacter -Ruminococcaceae | - | Odoribacter -Ruminococcaceae | - |  |
|  |  |  |  |  |  |  |  |  |  |  | Odoribacter – Ruminococcus [R] | - |  |
|  |  |  |  |  |  |  | Porphyromonadaceae- Clostridiaceae | - |  | | P. distasonis – Clostridiaceae 1 | - |  |
|  |  |  |  |  |  |  | Porphyromonadaceae - Lachnospiraceae | - | Parabacteroides -Coprococcus | - | P. distasonis-Coprococcus | - |  |
|  |  |  |  |  |  |  |  |  | Parabacteroides -Dorea | - |  | |  |
|  |  |  |  |  |  |  |  |  | Parabacteroides -Lachnospiraceae 2 | - | Parabacteroides – Lachnospiraceae 2 | - |  |
|  |  |  |  |  |  |  |  |  |  |  | P. distasonis - Lachnospiraceae 2 | - |  |
|  |  |  |  |  |  |  | Porphyromonadaceae - Ruminococcaceae | - |  | |  | |  |
|  |  |  |  |  |  |  | Rikenellaceae -Mogibacteriaceae | - | Rikenellaceae -Mogibacteriaceae | - | Rikenellaceae -Mogibacteriaceae | - |  |
|  |  |  | *Bacteroidia -Erysipelotrichia* | | *Bacteroidales - Erysipelotrichales* | | Porphyromonadaceae Erysipelotrichaceae | - |  | |  | |  |
|  |  |  |  |  |  |  | Rikenellaceae -Erysipelotrichaceae | - | Rikenellaceae – Eryispelotrichaceae 2 | - | Rikenellaceae -Erysipelotrichiaceae | - |  |
|  | | | | | | | | | | | | |  |
| *Bacteroidetes - Bacteroidetes* | | | *Bacteroidia - Bacteroidia* | | *Bacteroidales-Bacteroidales* | | *Bacteroidaceae - Bacteroidaceae* | | *Bacteroides - Bacteroides* | | Bacteroides –  B. uniformis | + |  |
|  |  |  |  |  |  |  |  |  |  |  | B. ovatus –  B. uniformis | + |  |
|  |  |  |  |  |  |  | Bacteroidaceae – Odoribacteriaceae | + | Bacteroides – Butyricimonas | + | Bacteroides – Butyricimonas | + |  |
|  |  |  |  |  |  |  |  |  | Bacteroides - Odoribacter | + | B. ovatus – Odoribacter | + |  |
|  |  |  |  |  |  |  |  |  |  |  | B. uniformis – Odoribacter | + |  |
|  |  |  |  |  |  |  | Bacteroidaceae – Porphyromonadaceae | + | Bacteroides – Parabacteroides | + | Bacteroides –  P. distasonis | + |  |
|  |  |  |  |  |  |  |  |  |  |  | B. ovatus –  P. distasonis | + |  |
|  |  |  |  |  |  |  |  |  |  |  | B. uniformis –  P. distasonis | + |  |
|  |  |  |  |  |  |  | Bacteroidaceae – Prevotellaceae | - | Bacteroides – Prevotella | - |  | |  |
|  |  |  |  |  |  |  | Bacteroidaceae - Rikenellaceae | + | Bacteroides - Rikenellaceae | + | Bacteroides – Rikenellaceae | + |  |
|  |  |  |  |  |  |  | *Odoribactericaeae - Porphyromondaceae* | | Odoribacter - Parabacteroides | + | Odoribacter –  P. distasonis | + |  |
|  |  |  |  |  |  |  | *Porphyromonadaceae - Porphyromonadaceae* | | *Parabacteroides - Parabacteroides* | | Parabacteroides –  P. distasonis | + |  |
|  |  |  |  |  |  |  | Porphyromonadaceae – Rikenellaceae | + | Parabacteroides - Rikenellaceae | + | Parabacteroides – Rikenellaceae | + |  |
|  | | | | | | | | | | | | | |
| Proteobacteria- Firmicutes | | - | *Betaproteobacteria - Clostridia* | | *Burkholderiales-Clostridiales* | | Alcaligenaceae – Ruminococcaceae | - |  | | | | |
|  |  |  |  |  |  |  | Alcaligenaceae - Clostridiaceae- | - | Sutterella –Clostridiaceae 2 | - | Sutterella – Clostridiaceae 2 | - | |
|  |  |  | *Gammaproteobacteria - Bacilli* | | Enterobacteriales - Turicibacteriales | - |  | | | | | | |
|  |  |  | Gammaproteobacteria – Clostridia | - | *Enterobacteriales-Clostridiales* | | Enterobacteriaceae – Mogibacteriaceae | + |  | | | | |
|  |  |  |  |  |  |  | *Enterobacteriaceae-Lachnospiraceae* | | *Enterobacteriaceae - Anaerostipes* | | Enterobacteriaceae-Anaerostipes | + | |
|  |  |  |  |  |  |  | *Ruminococcaceae-Enterobacteriaceae* | | Enterobacteriaceae – Oscillospira | - | Enterobacteriaceae – Oscillospira | + | |
|  |  |  | *Gammaproteobacteria - Erysipelotrichi* | | *Enterobacteriales-Erysipelotrichiales* | | *Enterobacteriaceae-Erysipelotrichiaceae* | | Enterobacteriaceae – Erysipelotrichiaceae 2 | + | Enterobacteriaceae – Erysipelotrichiaceae 2 | + | |
|  |  |  | Deltaproteobacteria - Clostridia | - | Desulfovibrionales – Clostridiales | - |  | | | | | | |
|  | | | | | | | | | | | | | |
| Proteobacteria-Bacteroidetes | | + | Betaproteobacteria-Bacteroidia | + | Burkholderiales-Bacteroidales | + | Alcaligenaceae-Bacteroidaceae | + | Sutterella-Bacteroides | + | Sutterella-Bacteroides | + | |
|  |  |  |  |  |  |  |  |  |  |  | Sutterella- B.uniformis | + | |
|  |  |  |  |  |  |  | Alcaligenaceae-Porphyromonadaceae | + | *Sutterella-Parabacteroides* | | Sutterella-P. distasonis | + | |
|  |  |  |  |  |  |  | Alcaligenaceae-Odoribacteriaceae | + | Sutterella-Butyricimonas | + | Sutterella-Butyricimonas | + | |
|  |  |  |  |  |  |  | *Alcaligenaceae-Rikenellaceae* | | Sutterella-Rikenellaceae | + |  | | |
|  |  |  | *Gammaproteobacteria-Bacteroidia* | | *Enterobacteriales-Bacteroidales* | | *Enterobacteriaceae-Rikenellaceae* | | Enterobacteriaceae-Rikenellaceae | - | Enterobacteriaceae -Rikenellaceae | - | |
|  | | | | | | | | | | | | | |
| *Firmicutes - Firmicutes* | | | *Bacilli-Clostridia* | | *Lactobacillales - Clostridiales* | | *Streptococcaceae – Lachnospiraceae* | | Streptococcus – Blautia | + |  | | |
|  |  |  |  |  |  |  | *Streptococcaceae – Ruminococcaceae* | | Streptococcus – Oscillospira | - | Streptococcus – Oscillospira | - | |
|  |  |  |  |  | *Turicibacteriales - Clostridiales* | | *Turicibacteriaceae – Clostridiales* | | *Turicibacter - Clostridiales* | | Turicibacter – Clostridiales 2 | + | |
|  |  |  |  |  |  |  | Turicibacteriaceae – Clostridiaceae | - | *Turicibacter - Clostridiaceae* | | Turicibacter – Clostridiaceae 1 | - | |
|  |  |  |  |  |  |  | *Turicibacteriaceae - Veillonellaceae* | | Turicibacter - Phascolarctobacterium | + | Turicibacter – Phascolarctobacterium | + | |
|  |  |  | *Clostridia – Clostridia* | | *Clostridiales - Clostridiales* | | *Clostridiales- Lachnospiraceae* | | Clostridiales 1 - Coprococcus | + |  | | |
|  |  |  |  |  |  |  |  |  | Clostridiales 1 –Ruminococcus[L] | - |  |  |  |
|  |  |  |  |  |  |  |  |  | Clostridiales 2 –Lachnospira | - | Clostridiales 2 – Lachnospira | - | |
|  |  |  |  |  |  |  | Clostridiales 1 – Ruminococcaceae | + | Clostridiales 1 – Ruminococcaceae | + | Clostridiales 1– Oscillospira | + | |
|  |  |  |  |  |  |  |  |  |  |  | Clostridiales 1– Ruminococcaceae | + | |
|  |  |  |  |  |  |  |  |  | Clostridiales 1 – Ruminococcus[R] | + | Clostridiales 1 – Ruminococcus[R] | + | |
|  |  |  |  |  |  |  | *Christensenellaceae - Lachnospiraceae* | | *Christensenellaceae – Ruminococcus [L]* | | Christensenellaceae – R. gnavus | + | |
|  |  |  |  |  |  |  | Christensenellaceae – Mogibacteriaceae | + |  | | | | |
|  |  |  |  |  |  |  | *Clostridiaceae-Clostridiaceae* | | Clostridiaceae 1-Clostridiaceae 2 | + | Clostridiaceae 1 – Clostridiaceae 2 | + | |
|  |  |  |  |  |  |  | Clostridiaceae – Lachnospiraceae | + | *Clostridiaceae - Dorea* | | Clostridiaceae 1 – Dorea 2 | + | |
|  |  |  |  |  |  |  | Clostridiaceae – Ruminococcaceae | + |  | | | | |
|  |  |  |  |  |  |  | *Lachnospiraceae - Lachnospiraceae* | | Lachnospiraceae 1 – Lachnospiraceae 2 | + | Lachnospiraceae 1 – Lachnospiraceae 2 | + | |
|  |  |  |  |  |  |  |  |  | Lachnospiraceae 1 - Anaerostipes | + |  | | |
|  |  |  |  |  |  |  |  |  | Lachnospiraceae 1 – Blautia | + | Lachnospiraceae 1 – Blautia 1 | + | |
|  |  |  |  |  |  |  |  |  | *Lachnospiraceae 1 – Coprococcus* | | Lachnospiraceae 1 – Coprococcus | + | |
|  |  |  |  |  |  |  |  |  | *Lachnospiraceae 2 – Coprococcus* | | Lachnospiraceae 2 – Coprococcus | + | |
|  |  |  |  |  |  |  |  |  | Lachnospiraceae 1 – Ruminococcus [L] | + | Lachnospiraceae 1 – Ruminococcus [L] | + | |
|  |  |  |  |  |  |  |  |  | *Lachnospiraceae 2 – Ruminococcus [L]* | | Lachnospiraceae 2 – Ruminococcus [L] | + | |
|  |  |  |  |  |  |  |  |  | *Anaerostipes – Dorea* | | Anaerostipes – Dorea 2 | + | |
|  |  |  |  |  |  |  |  |  | *Anaerostipes – Lachnospira* | | Anaerostipes – Lachnospira | - | |
|  |  |  |  |  |  |  |  |  | Blautia – Dorea | + | Blautia 1 – Dorea 2 | + | |
|  |  |  |  |  |  |  |  |  | *Blautia - Roseburia* | | Blautia 2 – Roseburia 2 | + | |
|  |  |  |  |  |  |  |  |  | Blautia – Ruminococcus[L] | + | Blautia 1 – Ruminococcus [L] | + | |
|  |  |  |  |  |  |  |  |  | Dorea – Ruminococcus[L] | + |  | | |
|  |  |  |  |  |  |  |  |  | Lachnospira – Ruminococcus[L] | + |  |  |  |
|  |  |  |  |  |  |  | *Lachnospiraceae – Ruminococcaceae* | | Anaerostipes – Oscillospira | - | Anaerostipes – Oscillospira | - | |
|  |  |  |  |  |  |  |  |  | Blautia – Faecalibacterium | - | Blautia 1 – F. prausnitzii | - | |
|  |  |  |  |  |  |  |  |  | Blautia – Oscillospira | - | Blautia 1 – Oscillospira | - | |
|  |  |  |  |  |  |  |  |  | Coprococcus – Oscillospira | + |  | | |
|  |  |  |  |  |  |  |  |  | Coprococcus – Ruminococcus[R] | + | Coprococcus – Ruminococcus [R] | + | |
|  |  |  |  |  |  |  |  |  | *Dorea - Ruminococcaceae* | | Dorea 1 – Ruminococcaceae | + | |
|  |  |  |  |  |  |  |  |  |  |  | Dorea 2 – Ruminococcaceae | - | |
|  |  |  |  |  |  |  |  |  | *Dorea – Oscillospira* | | Dorea 2 – Oscillospira | - | |
|  |  |  |  |  |  |  |  |  | *Lachnospira – Ruminococcaceae* | | Lachnospira – Ruminococcaceae | + | |
|  |  |  |  |  |  |  |  |  | *Lachnospiraceae 1 - Faecalibacterium* | | Lachnospiraceae 1 – F. prausnitzii | + | |
|  |  |  |  |  |  |  |  |  | Lachnospiraceae 2 – Faecalibacterium | + | Lachnospiraceae 2 – F. prausnitzii | + | |
|  |  |  |  |  |  |  |  |  | Roseburia – Ruminococcus[R] | + |  | | |
|  |  |  |  |  |  |  |  |  | Roseburia – Faecalibacterium | + |  |  |  |
|  |  |  |  |  |  |  |  |  | *Ruminococcus [L] - Faecalibacterium* | | R. gnavus –  F. prausnitzii | - | |
|  |  |  |  |  |  |  |  |  | Ruminococcus[L] – Peptostreptococcaceae | - | Ruminococcus[L] – Peptostreptococcaceae | - | |
|  |  |  |  |  |  |  |  |  | Ruminococcus[L] – Ruminococcaceae | - |  | | |
|  |  |  |  |  |  |  |  |  | Ruminococcus[L] – Oscillospira | - |  |  |  |
|  |  |  |  |  |  |  | *Lachnospiraceae – Veillonellaceae* | | Blautia – Phascolarctobacterium | - | Blautia 1 – Phascolarctobacterium | - | |
|  |  |  |  |  |  |  |  |  | Dorea – Phascolarctobacterium | - |  | | |
|  |  |  |  |  |  |  | *Ruminococcaceae – Ruminococcaceae* | | Ruminococcaceae - Faecalibacterium | + |  | | |
|  |  |  |  |  |  |  |  |  | Ruminococcaceae – Oscillospira | + | Ruminococcaceae – Oscillospira | + | |
|  |  |  |  |  |  |  |  |  | Ruminococcaceae – Ruminococcus[R] | + | Ruminococcaceae – Ruminococcus[R] | + | |
|  |  |  |  |  |  |  |  |  | Oscillospira – Ruminococcus[R] | + | Oscillospira – Ruminococcus[R] | + | |
|  |  |  | *Clostridia – Erysipleotrichia* | | Clostridiales – Erysipelotrichiales | + | *Clostridiales - Erysipelotrichiaceae* | | *Clostridiales - Coprobacillus* | | Clostridiales 1– Coprobacillus | + | |
|  |  |  |  |  |  |  | Christensenellaceae - Erysipelotrichiaceae | + |  | | | | |
|  |  |  |  |  |  |  | *Lachnospiraceae – Erysipelotrichiaceae* | | *Lachnospiraceae 1 – Erysipelotrichiaceae* | | Lachnospiraceae 1 – Erysipelotrichiaceae 1 | + | |
|  |  |  |  |  |  |  |  |  | Anaerostipes – Erysipelotrichiaceae 1 | + | Anaerostipes – Erysipelotrichiaceae 1 | + | |
|  |  |  |  |  |  |  |  |  | *Dorea – Erysipelotrichiaceae 1* | | Dorea 1 – Erysipelotrichiaceae 1 | + | |
|  |  |  |  |  |  |  |  |  | Dorea – Eubacterium | + |  | | |
|  |  |  |  |  |  |  |  |  | Lachnospira – Erysipelotrichiaceae 1 | + |  |  |  |
|  |  |  |  |  |  |  |  |  | *Lachnospira – Eubacterium* | | Lachnospira –  E. dolichum | - | |
|  |  |  |  |  |  |  |  |  | Lachnospiraceae 1 – Coprobacillus | - | Lachnospiraceae 1 – Coprobacillus | - | |
|  |  |  |  |  |  |  |  |  | Lachnospiraceae 2 – Coprobacillus | - |  | | |
|  |  |  |  |  |  |  |  |  | Roseburia – Coprobacillus | - | Roseburia 2 – Coprobacillus | - | |
|  |  |  |  |  |  |  | Ruminococcaceae – Erysipelotrichiaceae | + |  | | | | |
