# Supplementary material for: Attention Deficit Hyperactivity Disorder (ADHD) and the gut microbiome: An ecological perspective
Source: PLoS One. 2023 Aug 18;18(8):e0273890. doi: 10.1371/journal.pone.0273890 (PMC10437823; doi:10.1371/journal.pone.0273890)
Supplement: S4 Table — ATria rankings of all taxa over all MCNs, grouped by taxonomy. Dark orange = only ranked in Control, dark purple = only ranked in ADHD, light orange = higher ranked in Control, light purple = higher ranked in ADHD, grey = evenly ranked. Bold taxa are ranked #1. (DOCX) [file pone.0273890.s011.docx]

| **Phylum** | **Class** | **Order** | **Family** | **Genus** | **Lowest Possible** |
| --- | --- | --- | --- | --- | --- |
| **Actinobacteria (#1/#1)** | Actinobacteria (#2/NR) | Bifidobacteriales (#T2/ #T5) | Bifidobacteriaceae (#5/NR) | Bifidobacterium (NR/#T19) | Bifidobacterium 1 (#10/NR) |
|  |  |  |  |  | **Bifidobacterium 2 (#1/11)** |
|  |  |  |  |  | B. longum (#15/NR) |
|  | **Coriobacteria (#1/NR)** | Coriobacteriales (NR/#T5) | Coriobacteriaceae (NR/#4) | Adlercruetzia (NR/#17) | Adlercruetzia (#14/NR) |
|  |  |  |  | Collinsella (NR/#T19) | C. aerofaciens (NR/#15) |
|  |  |  |  | Coriobacteriaceae (#18/NR) |  |
|  |  |  |  | Eggerthella (#4/NR) | E. lenta (#T25/NR) |
|  | | | | | |
| Bacteroidetes (NR/#T2) | **Bacteroidia (NR/#T1)** | **Bacteroidales (#1/#1)** | Bacteroidaceae (#T9/#T12) | Bacteroides (#10/#T23) | B. uniformis (#12/#7) |
|  |  |  |  |  | B. ovatus (#2/#T20) |
|  |  |  |  |  | Bacteroides (#T23/#T28) |
|  |  |  | Odoribacteriaceae (NR/#2) | Odoribacter (#9/#T21) | Odoribacter (#3/#T20) |
|  |  |  |  | Butyricimonas (NR/#4) | Butyricimonas (NR/#17) |
|  |  |  | **Porphyromonadaceae (#T7/#1)** | Parabacteroides (#T12/NR) | Parabacteroides (#T19/#T24) |
|  |  |  |  |  | P. distasonis (NR/#T24) |
|  |  |  | Prevotellaceae (NR/#5) | Prevotella (NR/#18) |  |
|  |  |  | Rikenellaceae (#T7/#3) | Rikenellaceae (#T12/#8) | Rikenellaceae (#T19/#8) |
|  | | | | | |
| Firmicutes (#2/#T2) | *Bacilli (NR/NR)* | *Lactobacillales (NR/NR)* | Streptococcaceae (NR/#T12) | Streptococcus (NR/#T23) |  |
|  |  | Turicibacterales (#T3/NR) | Turicibacteriaceae (NR#T10) | Turicibacter (NR/#14) | Turicibacter (NR/#10) |
|  | Clostridia (#T3/#T3) | Clostridiales (#T2/#3) | Christensenellaceae (#2/NR) | *Christensenellaceae (NR/NR)* | Christensenellaceae (#18/NR) |
|  |  |  | Clostridiaceae (#6/#T10) | Clostridiaceae 1 (NR/#T21) | Clostridiaceae 1 (NR/#3) |
|  |  |  |  | Clostridiaceae 2 (NR/#3) | Clostridiaceae 2 (NR/#T16) |
|  |  |  | **Clostridiales 1 (#1/#T8)** | *Clostridiales 1 (NR/NR)* | Clostridiales 1 (#T31/#4) |
|  |  |  | *Clostridiales 2 (NR/NR)* | Clostridiales 2 (#5/NR) | Clostridiales 2(#T27/NR) |
|  |  |  | *Lachnospiraceae (NR/NR)* | Anaerostipes (#T19/NR) | Anaerostipes (#6/#T26) |
|  |  |  |  | Blautia (#T16/#13) | Blautia 1 (NR/#19) |
|  |  |  |  |  | Blautia 2 (#9/#T22) |
|  |  |  |  | Coprococcus (NR/#5) | Coprococcus (#T21/#6) |
|  |  |  |  | Dorea (#T14/#12) | Dorea 2 (#13/NR) |
|  |  |  |  | Lachnospira (NR/#16) | Lachnospira (#T27/#5) |
|  |  |  |  | Lachnospiraceae 1 (#19/#15) | Lachnospiraceae 1 (#17/#2) |
|  |  |  |  | Lachnospiraceae 2 (#7/NR) | Lachnospiraceae 2 (#8/#14) |
|  |  |  |  | *Roseburia (NR/NR)* | Roseburia 1 (NR/#T28) |
|  |  |  |  |  | Roseburia 2(#T25/#T22) |
|  |  |  |  | **Ruminococcus (#1/#11)** | R. gnavis (NR/#12) |
|  |  |  | Mogibacteriaceae (NR/#T6) | Mogibacteriaceae (NR/#6) |  |
|  |  |  | *Peptostreptococcaceae (NR/NR)* | *Peptostreptococcaceae (NR/NR)* | Peptostreptococcaceae (F) (#7/NR) |
|  |  |  | Ruminococcaceae (#4/#T8) | Faecalibacterium (#T16/#2) | F. prausnitzii (#11/NR) |
|  |  |  |  | Oscillospira (#3/#10) | Oscillospira (#5/#9) |
|  |  |  |  | Ruminococcaceae (#8/#9) | Ruminococcaceae (#4/#18) |
|  |  |  |  | Ruminococcus (#2/NR) | Ruminococcus (#T21/NR) |
|  |  |  | *Veillonellaceae (NR/NR)* | *Phascolarctobacterium (NR/NR)* | Phascolarctobacterium (NR/#13) |
|  |  |  |  | *Veillonella (NR/NR)* | V. dispar (#T29/NR) |
|  | *Erysipelotrichia (NR/NR)* | Erysipelotrichales (NR/#T3) | Erysipelotrichaceae (#3/NR) | **Coprobacillus (NR/#1)** | **Coprobacillus (#T31/#1)** |
|  |  |  |  | Erysipelotrichaceae 1 (#6/NR) | Erysipelotrichaceae 1 (#16/NR) |
|  |  |  |  | Erysipelotrichaceae 2 (#11/NR) |  |
|  |  |  |  | Eubacterium (#T14/NR) | E. dolicum (#T29/NR) |
|  | | | | | |
| *Proteobacteria (NR/NR)* | **Betaproteobacteria (NR/#T1)** | *Burkholderiales*  *(NR/NR)* | Alcaligenaceae (#T9/NR) | *Sutterella (NR/NR)* | Sutterella (#T23/NR) |
|  | Deltaproteobacteria (NR/#T3) | Desulfovibrionales (#2/NR) |  |  |  |
|  | Gammaproteobacteria (#T3/NR) | Enterobacteriales (#T3/NR) | Enterobacteriaceae (NR/#T6) | Enterobacteriaceae (NR/#7) | Enterobacteriaceae (NR/#T26) |
