# Supplementary material for: Attention Deficit Hyperactivity Disorder (ADHD) and the gut microbiome: An ecological perspective
Source: PLoS One. 2023 Aug 18;18(8):e0273890. doi: 10.1371/journal.pone.0273890 (PMC10437823; doi:10.1371/journal.pone.0273890)
Supplement: S5 Table — Communities within Control and ADHD family-level MCNs. Phylum: purple = Bacteroidetes, yellow = Firmicutes, brown = Actinobacteria, blue = Proteobacteria. (DOCX) [file pone.0273890.s012.docx]

|  | | **Control** | | | **ADHD** | | |
| --- | --- | --- | --- | --- | --- | --- | --- |
| **Community** | **Cluster Type** | **Cluster** | **Taxon** | **Phylum** | **Cluster** | **Taxon** | **Phylum** |
| **Bacteroidetes-dominant (B)** | **Bacteroidaceae-dominant (BB)** | **BB** | **Bacteroidaceae** |  | **BB** | **Bacteroidaceae*** |  |
|  |  | **BB** | **Porphyromonadaceae*** |  | **BB** | **Porphyromonadaceae** |  |
|  |  | **BB** | **Alcaligenaceae** |  | **BB** | **Alcaligenaceae** |  |
|  |  | **BB** | **Rikenellaceae** |  | **BB** | **Rikenellaceae** |  |
|  |  |  | | | **BB** | *Odoribacteraceae* |  |
|  |  |  |  |  | **BB** | *Streptococcaceae* |  |
| **Firmicutes-dominant (F)** | **Lachnospiraceae-dominant (FL)** | **FL** | **Lachnospiraceae*** |  | **FL** | **Lachnospiraceae** |  |
|  |  | **FL** | **Clostridiaceae** |  | **FL** | **Clostridiaceae*** |  |
|  |  | **FL** | **Bifidobacteriaceae** |  | **FL** | **Bifidobacteriaceae** |  |
|  |  |  | | | **FL** | *Coriobacteriaceae* |  |
|  | **Ruminococcaceae-dominant (FR)** | **FR** | **Ruminococcaceae** |  | **FR** | **Ruminococcaceae*** |  |
|  |  | **FR** | **Clostridiales 1*** |  | **FR** | **Clostridiales 1** |  |
|  |  | **FR** | *Coriobacteriaceae* |  | **FR** | *Erysipelotrichiaceae* |  |
|  | **Mixed (FM)** | ***FM*** | *Mogibacteriaceae* |  |  | | |
|  |  | ***FM*** | *Christensellaceae** |  |  |  |  |
|  |  | ***FM*** | *Erysipelotrichiaceae* |  |  |  |  |
| **Mixed**  **(M)** | **N/A** |  | | | ***M*** | *Mogibacteriaceae* |  |
|  |  |  |  |  | ***M*** | *Enterobacteriaceae* |  |
