# Supplementary material for: Attention Deficit Hyperactivity Disorder (ADHD) and the gut microbiome: An ecological perspective
Source: PLoS One. 2023 Aug 18;18(8):e0273890. doi: 10.1371/journal.pone.0273890 (PMC10437823; doi:10.1371/journal.pone.0273890)
Supplement: S6 Table — Communities within Control and ADHD genus-level MCNs. Phylum: purple = Bacteroidetes, yellow = Firmicutes, brown = Actinobacteria, blue = Proteobacteria. (DOCX) [file pone.0273890.s013.docx]

|  |  | **Control** | |  | **ADHD** | |  |
| --- | --- | --- | --- | --- | --- | --- | --- |
| **Community** | **Cluster Type** | **Cluster** | **Taxon** | **Phy** | **Cluster** | **Taxon** | **Phy** |
| **Bacteroidetes-dominant (B)** | **Bacteroidaceae-dominant (BB)** | **B** | **Bacteroides** |  | **B** | **Bacteroides*** |  |
|  |  | **B** | **Parabacteroides*** |  | **B** | **Parabacteroides** |  |
|  |  | **B** | **Sutterella** |  | **B** | **Sutterella** |  |
|  |  | **B** | **Rikenellaceae** |  | **B** | **Rikenellaceae** |  |
|  |  | **B** | *Odoribacter* |  | **B** | *Butyricimonas* |  |
|  |  | **B** | *Adlercruetzia* |  | **B** | *Streptococcus* |  |
|  |  |  | | | **B** | *Clostridium* |  |
| **Firmicutes-dominant (F)** | **Lachnospiraceae-dominant (FL)** | **FL1** | **Lachnospiraceae 1*** |  | **FL1** | **Lachnospiraceae 1*** |  |
|  |  | **FL1** | **Lachnospiraceae 2** |  | **FL1** | **Lachnospiraceae 2** |  |
|  |  | **FL1** | *Anaerostipes* |  | **FL1** | Blautia |  |
|  |  | **FL2** | **Lachnospira** |  | **FL2** | **Lachnospira** |  |
|  |  | **FL2** | *Erysipelotrichiaceae 1* |  | **FL2** | Ruminococcus [L]* |  |
|  |  |  | | | **FL2** | *Dorea* |  |
|  |  | ***FL3*** | Blautia |  |  | | |
|  |  | ***FL3*** | Ruminococcus [L] |  |  |  |  |
|  | **Ruminococcaceae-dominant (FR)** | **FR** | **Ruminococcaceae** |  | **FR** | **Ruminococcaceae*** |  |
|  |  | **FR** | **Ruminococcus [R]*** |  | **FR** | **Ruminococcus [R]** |  |
|  |  | **FR** | **Oscillospira** |  | **FR** | **Oscillospira** |  |
|  |  | **FR** | **Clostridiales 1** |  | **FR** | **Clostridiales 1** |  |
|  |  | **FR** | *Coprococcus* |  | **FR** | *Roseburia* |  |
|  |  |  | | | **FR** | *Faecalibacterium* |  |
|  | **Clostridiaceae-dominant (FR)** | ***FC*** | *Clostridiaceae 1* |  |  | | |
|  |  | ***FC*** | *Clostridiaceae 2* |  |  |  |  |
|  | **Turicibacter-core (FT)** |  | | | ***FT*** | *Turicibacter* |  |
|  |  |  |  |  | ***FT*** | *Phascolarctobacterium* |  |
|  | **Firmicutes, Mixed (FT)** | ***FM*** | *Dorea* |  |  | | |
|  |  | ***FM*** | *Eubacterium* |  |  |  |  |
| **Actinobacteria (A)** | **Actinobacteria, Mixed (AM)** | **AM** | **Bifidobacterium** |  | **AM** | **Bifidobacterium** |  |
|  |  | **AM** | **Collinsella** |  | **AM** | **Collinsella** |  |
|  | **Actinobacteria, Coriobacteriaceae-dominant (AC)** |  | | | ***AC*** | *Coriobacteriaceae* |  |
|  |  |  |  |  | ***AC*** | *Eubacterium* |  |
|  |  |  |  |  | ***AC*** | *Adlercruetzia** |  |
| **Mixed (M)** | **N/A** | **M** | *Christenellaceae* |  | **M** | *Erysipelotrichiaceae 2* |  |
|  |  | **M** | *Coriobacteriaceae* |  | **M** | *Enterobacteriaceae* |  |
