# Supplementary material for: Attention Deficit Hyperactivity Disorder (ADHD) and the gut microbiome: An ecological perspective
Source: PLoS One. 2023 Aug 18;18(8):e0273890. doi: 10.1371/journal.pone.0273890 (PMC10437823; doi:10.1371/journal.pone.0273890)
Supplement: S7 Table — Communities within Control and ADHD MCNs using the lowest possible level of taxonomic classification. Phylum: purple = Bacteroidetes, yellow = Firmicutes, brown-Actinobacteria, blue = Proteobacteria. (DOCX) [file pone.0273890.s014.docx]

|  |  | **Control** | |  | **ADHD** | |  |
| --- | --- | --- | --- | --- | --- | --- | --- |
| **Community** | **Cluster Type** | **Cluster** | **Taxon** | **Phy** | **Cluster** | **Taxon** | **Phy** |
| **Bacteroidetes-dominant (B)** | **Bacteroidetes, Bacteroidaceae-dominant (BB)** | **BB** | **Bacteroides*** |  | **BB** | **Bacteroides*** |  |
|  |  | **BB** | **Bacteroides uniformis** |  | **BB** | **Bacteroides uniformis** |  |
|  |  | **BB** | **Sutterella** |  | **BB** | **Sutterella** |  |
|  |  |  | | | **BB** | Parabacteroides distasonis |  |
|  |  |  |  |  | **BB** | Rikenellaceae |  |
|  |  |  |  |  | **BB** | *Butyricimonas* |  |
|  |  |  |  |  | **BB** | *Bifidobacterium longum* |  |
|  | **Bacteroidetes, Mixed (BM)** | **BM1** | **Odoribacter** |  | **BM1** | **Odoribacter** |  |
|  |  | **BM1** | **Bacteroides ovatus** |  | **BM1** | **Bacteroides ovatus** |  |
|  |  | **BM1** | Parabacteroides distasonis* |  |  | | |
|  |  | **BM1** | *Adlercruetzia* |  |  |  |  |
|  |  | ***BM2*** | Rikenellaceae |  |  |  |  |
|  |  | ***BM2*** | *Parabacteroides* |  |  |  |  |
| **Firmicutes-dominant,** | **Firmicutes,**  **Lachnospiraceae-dominant** | **FL1** | **Lachnospiraceae 1** |  | **FL1** | **Lachnospiraceae 1** |  |
|  |  | **FL1** | **Lachnospiraceae 2*** |  | **FL1** | **Lachnospiraceae 2*** |  |
|  |  | **FL1** | **Coprococcus** |  | **FL1** | **Coprococcus** |  |
|  |  |  | | | **FL1** | *Ruminococcus [L]* |  |
|  |  |  |  |  | **FL1** | Blautia 1 |  |
|  |  |  |  |  | **FL1** | Dorea 2 |  |
|  |  |  |  |  | **FL1** | *Faecalibacterium prausnitzii* |  |
|  |  | **FL2** | **Anaerostipes** |  | **FL2** | **Anaerostipes** |  |
|  |  | **FL2** | **Erysipelotrichiaceae*** |  | **FL2** | **Erysipelotrichiaceae** |  |
|  |  | **FL2** | *Dorea 1* |  | **FL2** | *Enterobacteriaceae** |  |
|  |  | **FL3** | Blautia 1* |  | ***FL3*** | *Blautia 2* |  |
|  |  | **FL3** | Dorea 2 |  | ***FL3*** | *Roseburia 2* |  |
|  |  | **FL3** | *Bifidobacterium longum* |  |  | | |
|  | **Firmicutes, Clostridiaceae-dominant** | ***FC*** | *Clostridiaceae 1* |  |  | | |
|  |  | ***FC*** | *Clostridiaceae 2* |  |  |  |  |
|  | **Firmicutes, Ruminococcaceae-dominant** | **FR** | **Ruminococcaceae*** |  | **FR** | **Ruminococcaceae** |  |
|  |  | **FR** | **Ruminococcus [R]** |  | **FR** | **Ruminococcus [R]** |  |
|  |  | **FR** | **Oscillospira** |  | **FR** | **Oscillospira** |  |
|  |  | **FR** | **Clostridiales 1** |  | **FR** | **Clostridiales 1*** |  |
|  |  | **FR** | *Coprobacillus* |  | **FR** | *Bifidobacterium adolescentis* |  |
|  |  | **FR** | *Enterobacteriaceae* |  |  | | |
|  |  | **FR** | *Lachnospira* |  |  |  |  |
|  | **Firmicutes, Mixed** | ***FM*** | *Ruminococcus gnavis* |  |  | | |
|  |  | ***FM*** | *Christensenellaceae** |  |  |  |  |
|  |  | ***FM*** | *Coriobacteriaceae* |  |  |  |  |
|  | **Firmicutes, Turicibacter** | **FT** | **Turicibacter** |  | **FT** | **Turicibacter*** |  |
|  |  | **FT** | *Ruminococcus [L]* |  | **FT** | *Phascolarctobacterium* |  |
|  |  | **FT** | *Bifidobacterium 1** |  | **FT** | *Clostridiales 1* |  |
|  |  |  | | | **FT** | *Parabacteroides* |  |
| **Mixed** | **N/A** | **M** | *Eggerthella lenta* |  | **M** | *Coriobacteriaceae* |  |
|  |  | **M** | *Roseburia 2* |  | **M** | *Clostridiaceae 2* |  |
